# Computational Model Reveals Limited Correlation between Germinal Center B-Cell Subclone Abundancy and Affinity: Implications for Repertoire Sequencing

**DOI:** 10.3389/fimmu.2017.00221

**Published:** 2017-03-06

**Authors:** Polina Reshetova, Barbera D. C. van Schaik, Paul L. Klarenbeek, Marieke E. Doorenspleet, Rebecca E. E. Esveldt, Paul-Peter Tak, Jeroen E. J. Guikema, Niek de Vries, Antoine H. C. van Kampen

**Affiliations:** ^1^Biosystems Data Analysis, Swammerdam Institute for Life Sciences, University of Amsterdam, Amsterdam, Netherlands; ^2^Bioinformatics Laboratory, Academic Medical Center, University of Amsterdam, Amsterdam, Netherlands; ^3^Amsterdam Rheumatology and Immunology Center, Academic Medical Center, Amsterdam, Netherlands; ^4^Department of Clinical Immunology and Rheumatology, Academic Medical Center, University of Amsterdam, Amsterdam, Netherlands; ^5^Department of Pathology, Academic Medical Center, University of Amsterdam, Amsterdam, Netherlands

**Keywords:** computational modeling, repertoire sequencing, germinal center, clonal expansion, BCR affinity

## Abstract

Immunoglobulin repertoire sequencing has successfully been applied to identify expanded antigen-activated B-cell clones that play a role in the pathogenesis of immune disorders. One challenge is the selection of the Ag-specific B cells from the measured repertoire for downstream analyses. A general feature of an immune response is the expansion of specific clones resulting in a set of subclones with common ancestry varying in abundance and in the number of acquired somatic mutations. The expanded subclones are expected to have BCR affinities for the Ag higher than the affinities of the naive B cells in the background population. For these reasons, several groups successfully proceeded or suggested selecting highly abundant subclones from the repertoire to obtain the Ag-specific B cells. Given the nature of affinity maturation one would expect that abundant subclones are of high affinity but since repertoire sequencing only provides information about abundancies, this can only be verified with additional experiments, which are very labor intensive. Moreover, this would also require knowledge of the Ag, which is often not available for clinical samples. Consequently, in general we do not know if the selected highly abundant subclone(s) are also the high(est) affinity subclones. Such knowledge would likely improve the selection of relevant subclones for further characterization and Ag screening. Therefore, to gain insight in the relation between subclone abundancy and affinity, we developed a computational model that simulates affinity maturation in a single GC while tracking individual subclones in terms of abundancy and affinity. We show that the model correctly captures the overall GC dynamics, and that the amount of expansion is qualitatively comparable to expansion observed from B cells isolated from human lymph nodes. Analysis of the fraction of high- and low-affinity subclones among the unexpanded and expanded subclones reveals a limited correlation between abundancy and affinity and shows that the low abundant subclones are of highest affinity. Thus, our model suggests that selecting highly abundant subclones from repertoire sequencing experiments would not always lead to the high(est) affinity B cells. Consequently, additional or alternative selection approaches need to be applied.

## 1 Introduction

The adaptive immune system is a key component of our defense against pathogens and comprises highly specialized cells and processes. Its humoral component is responsible for memory B-cell formation and high-affinity antibody (Ab) production resulting from affinity maturation in germinal centers (GCs) (1, 2). During this process, GC B cells undergo multiple rounds of proliferation, somatic hypermutation (SHM), and selection to improve their affinity for the given antigen (Ag). This results in a dynamic ensemble of low- and high-affinity B-cell subclones comprising variants of a clone within a V(D)J family produced by SHM. Higher-affinity cells have increased chance to be positively selected for further rounds of proliferation and SHM, or for differentiation to memory cells and plasma cells.

Repertoire sequencing using high-throughput sequencing enables the determination of T- and B-cell repertoires in (clinical) samples by sequencing the expressed V, D, and J gene segments (3–6). Immune responses typically involve the initiation and coexistence of up to several hundreds of GCs, which emerge over an extended period of time (7–9). Consequently, B-cell repertoire sequencing of clinical samples typically identifies (sub)clones originating from a multitude of Ag-activated B cells and GCs or even from responses to multiple Ags. Despite this complexity, we and others successfully used repertoire sequencing for the identification of B cells involved in (auto)immune disorders or infection. One challenge is to select the Ag-specific B cells from the measured repertoire. A general feature of an immune response is the expansion of specific clones resulting in a set of subclones with common ancestry varying in abundance and the number of acquired somatic mutations. These expanded subclones will have BCR-binding affinities for the Ag that are expected to be higher than affinities of the naive B cells in the background population. This is a direct consequence of the higher initial affinities of the activated B cells for the Ag and the subsequent affinity maturation process. For this reason, several groups successfully proceeded or suggested selecting highly abundant subclones from the repertoire (10–15). Given the nature of affinity maturation, one would expect that abundant subclones are of high affinity, but since repertoire sequencing only provides information about abundancies, this can only be verified with additional experiments. Experimental determination of BCR affinities for a reasonable number of subclones is feasible as is, for example, demonstrated by vaccination studies but very labor intensive (5, 16). Since B cells are destructed in the sequencing experiment, affinity analysis requires either selective cloning of the individual B cells or expression of single BCRs in cloning systems. Currently, these requirements prohibit a routine analysis of affinity in BCR repertoire studies. Moreover, affinity measurement requires knowledge of the Ag, which is often not available for clinical samples. Consequently, we do not know if highly abundant subclone(s) are generally also of the high(est) affinity. We developed a computational model of a single GC to gain insight in the putative affinity distribution among expanded and unexpanded subclones identified by B-cell repertoire sequencing. Inspired by existing models of affinity maturation [e.g., Ref. (17–20)], we implemented a mathematical model that comprises a large evolving set of ordinary differential equations (ODEs) providing information about the abundancy and affinity of individual subclones emerging during the GCR. We did not use one of the published models, since existing ODE models do not track individual subclones, while agent-based models [e.g., Ref. (20)] are faced with the additional complexity of GC spatial dynamics, which we aimed to avoid. Moreover, most models are not available as a software implementation.

We show that our computational model is in agreement with typical GC dynamics. We also show that the amount of expansion of selected B-cell lineages from repertoire data acquired from a human lymph node is qualitatively comparable to the level of expansion observed in the simulated data. Given this support for our model, we subsequently inspected the affinities and abundancies of the individual subclones from the simulations and found that the expanded and unexpanded B-cell subclone compartments each comprise a mixture of high- and low-affinity cells, i.e., there is only partial correlation between affinity and abundancy of subclones within a clonal family. Moreover, the low abundant subclones were of highest affinity. Although further work is required to experimentally validate these results, our simulations suggest that selection of highly abundant subclones from BCR repertoires will not necessarily lead to the highest affinity subclones. Therefore, additional or alternative selection strategies should be applied.

## 2 Materials and Methods

### 2.1 Sample and Experimental Data

We selected a single sample for analysis and comparison to the simulation results. This sample represents leukocytes isolated from a lymph node from an otherwise healthy human individual, without ongoing infection (represented in biochemical parameters such as C-reactive protein). The sample was taken as described earlier (21). The needle biopsy was stored in liquid nitrogen until use. Total RNA was isolated using polytron tissue homogenizer (Kinematica AG, Littau-Lucerne, Switzerland) and AllPrep DNA/RNA mini kit (#80204, Qiagen, Venlo, The Netherlands) according to manufacturer’s protocol and stored at −80°C until further use. The BCR repertoire was analyzed using dedicated primers. This linear amplification protocol has been extensively described earlier (3, 10, 22). Samples were prepared for sequencing according to the manual for amplicon sequencing (Roche FLX Titanium platform). Study candidates were informed about the background and purpose of the study and the biopsy procedure and possible complications (in particular hematoma). Written informed consent was obtained. Ethics approval was provided by the Ethics Committee Academic Medical Center/University of Amsterdam. Repertoire sequencing resulted in 7,771 reads (6,777 unique reads). Processing of the sequence data was performed as described in Ref. (3). In brief, reads from a multiplexed sequence run were separated by their multiplex identifiers (MID) and aligned against the IMGT database (23) with BLAT (24) to identify the corresponding V and J segments. Subsequently, each read was translated to a peptide sequence, and the CDR3 sequence was determined by identifying conserved motifs in the V and J segment that delineate the CDR3 (25). Consequently, only in-frame reads were used. Sequences with uncalled bases in their CDR3 region were excluded from analysis. This resulted in 4,454 unique subclones (clones within a VJ family defined as a peptide with a unique V and J assignment, and unique CDR3 sequence). This number of sequence reads is sufficient to represent most (expanded) subclones but may miss subclones occurring at very low frequencies. A full analysis and presentation of this and other lymph node samples will be part of future paper.

### 2.2 The Mathematical Model

We developed a mathematical model using ordinary differential equations (ODEs) to describe the dynamics of individual subclones during the GCR. This model is implemented in the R statistical environment version 3.2.2 (26) using R packages deSolve (version 1.12) (27), R6 (version 2.1.2), ggplot 2.0, and beeswarm 0.2.1. The software is available as open source (GPLv3) on request from the author.

#### 2.2.1 Overall Simulation Setup

**Figure 1:**
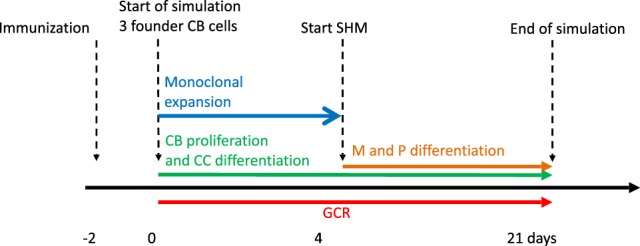
**Simulation time line of the germinal center reaction (GCR)**. The GCR starts with 3 founder B cells (affinities 0.1, 0.2 and 0.3 μM) 2 days after immunization and continues for 21 days.

Our simulation framework represents a simplified but adequate model of the GCR (1, 2) (Figure 1). Briefly, prior to the GCR, B cells and T cells are activated by recognition of their cognate antigen in the primary follicle and T-cell zone, respectively (day −2 in Figure 1). Activated B cells and T cells migrate to the interfollicular region and interact resulting in the full activation of B cells, while the T cells differentiate to T follicular helper cells (Tfh). Two days after immunization, the GCR is initiated (day 0 in our simulation) with the Tfh cells and activated B cells migrating into the follicle, which is characterized by a network of follicular dendritic cells (FDCs). Here, the B cells engage in a rapid monoclonal expansion to over 10,000 cells at day 4 forming the GC. During this expansion, a dark zone comprising centroblasts (CBs) and a light zone comprising centrocytes (CC), FDCs, and Tfh cells are established. The dark zone is the site of B-cell clonal expansion and BCR diversification through SHM, producing novel subclones. The GC light zone is the site of positive B-cell selection through Ag and Tfh binding and signaling. Together, these processes are responsible for B-cell affinity maturation. SHM has been reported to start at day 7 post-immunization in mice (28). Oprea and Perelson (17) assumed that the GC is initiated 3 days after immunization and, correspondingly, start SHM at day 4 of the GCR in their model. Others reported that SHM starts 2 days post-immunization (29) or even prior to GC formation (30). Following Oprea and Perelson, we also start SHM at day 4 in our simulations. Following monoclonal expansion, memory cells and plasma cells are being produced (day 4 in our simulation). Although the precise mechanisms and timing of the output cells are not well understood (31), it has been proposed that initially memory B cells are produced while at later stages the GCR is dedicated toward (higher-affinity) long-lived plasma cells (32, 33). In our model the production of memory and plasma cells starts at the same moment (day 4), but we made the rate of plasma cell differentiation dependent on the absolute affinity of the CCs resulting in a low plasma cell output during early stages of the GCR. Since we were not interested in the production of output cells, these are not further discussed in this paper. Our simulation starts at day 0 with three founder B cells (CBs) with different affinities and terminates after 21 days, the life span of an average GC. Consequently, we do not model GC shutdown since its mechanisms remain to be established. Our model does not explicitly includes the dark/light zones, Ags, FDCs, or Tfh cells since we are neither interested in the spatial dynamics nor in the precise selection mechanisms but rather in modeling subclonal diversity, expansion, and affinity. Therefore, to avoid an overly complex model, we represent the Ag and Tfh survival signals with sigmoidal functions as explained below.

#### 2.2.2 Somatic Hypermutation, Subclones, and Affinity

**Figure 2:**
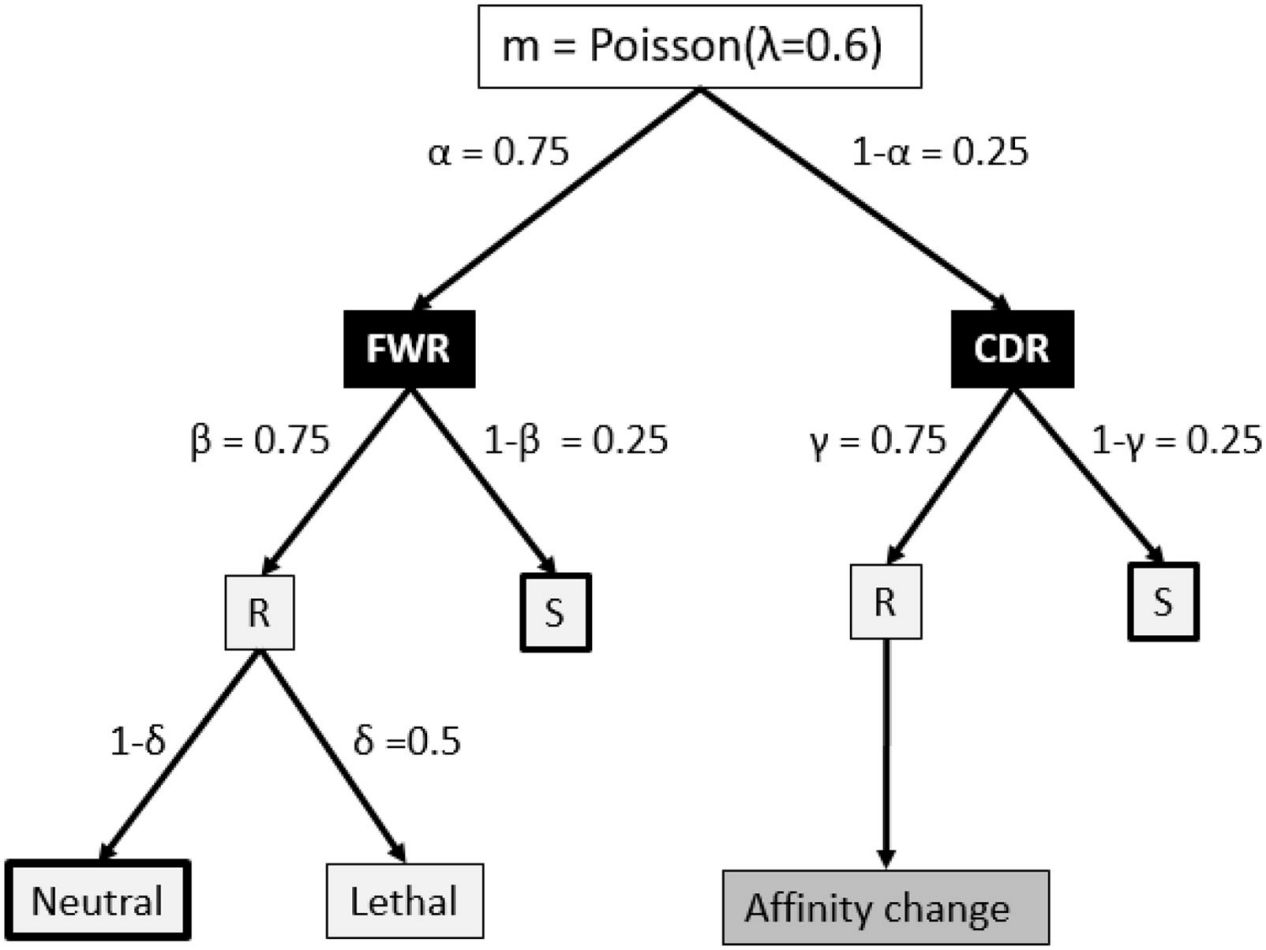
**Fate of each somatic hypermutation**. After each CB division the daughter cells are affected by *m* ≥ 0 mutations that affect the framework region (FWR) with a probability of *α* or the complementary-determining region (CDR). A mutation may replace (R) an amino acid of the Ig FWR or CDR region with probability *β* and *γ*, respectively. A mutation in the FWR is lethal with probability *δ*. A replacement mutation in the CDR is neutral or changes the affinity of the subclone. Part of our simulations neglect mutations indicated by the thick boxes to produce subclones at the peptide level. Probabilities in this tree are according to Ref. (18) and references therein.

The V, D, and J segments that make up the BCR cover four framework regions (FWRs) providing the Ab structural framework and three Ag-binding complementary determining regions (CDRs) (34, 35). Our model considers the FWR and CDR regions without an explicit nucleotide representation of the BCR, but instead, using a decision tree that decides on the fate of each individual SHM (18, 36, 37) (Figure 2). This tree involves probabilities for silent (synonymous mutations), lethal FWR, and affinity-changing CDR mutations. The probabilities for replacement and silent mutations were determined from many mice germline sequences. The probability of the lethal mutations was based on studies that analyzed mutation patterns in real sequences. To determine the number of mutations during each CB cell division, we defined the BCR to have a length of 600 nucleotides (i.e., one light and heavy chain). Given that the SHM rate is 10^−3^ per bp per division this results in 0.6 mutations per division. We model this as a Poisson distribution *m* = *Poisson*(*λ* = 0.6), and consequently, each cell acquires 0, 1, or more mutations after each cell division. The mutation decision tree distinguishes the CDRs and their surrounding structural FWRs but does not differentiate between CDR1, CDR2, and CDR3 (34, 35).

In repertoire sequencing one is usually interested determining the population of (sub)clones in an immune response. Each of these subclones has its own binding affinity for the Ag. Since the CDR3 region is the main determinant in Ag-binding, one generally defines and discriminates these subclones on the basis of their unique CDR3 peptide sequence within a VJ family. Alternatively, we can also define a subclone as having a unique BCR nucleotide sequences (i.e., V-CDR3-J). In the first situation, only non-synonymous SHMs in the CDR3 region produce new subclones, while in the second situation each non-lethal SHM results in a new subclone. The mutation decision tree (Figure 2) is defined at the level of the nucleotide sequence, and consequently, in our simulation we implicitly define and track subclones at the nucleotide level throughout the GCR. Consequently, each SHM generates a new subclone that is initially represented as a single CB that subsequently proliferates and differentiates to coexist as CB, CC, memory cell, and plasma cell at succeeding time points. Alternatively, we may consider only CDR replacement mutations to define and track subclones at the peptide level. In this situation, only non-lethal replacement mutations in the CDR generate new subclones. Since the tree does not specifically distinguish CDR3 from CDR1 and CDR2, our simulations at the peptide level effectively includes all three CDRs, which may give an overestimation of the number of unique clones compared to only considering the CDR3 as is done in repertoire sequencing experiments. However, since all three CDR regions are involved in Ag binding, the simulation might be more realistic. Subclones with CB cell counts less than one (a result from using continuous differential equations; see below) are kept in our simulation but are not further be affected by SHM to avoid the generation of new subclones from these cells.

Each subclone in our model has a unique BCR with an absolute affinity *σ* that specifies the interaction strength with the Ag. The affinities of the three single cell founder CBs are set to arbitrary but different low-affinity values (0.1, 0.3, and 0.5 μM). Three different values were chosen to establish an initial level competition between the founder cells. The magnitude of the initial affinities does not affect the dynamics of our model since this depends on relative affinities (see below). Only plasma cell output depends on absolute affinities. For each affinity changing mutation (Figure 2) the affinity of the affected subclone is updated according to *σ_new subclone_* = *σ_parent_* + *Δσ* where *Δσ* is drawn from a distribution *f*(*σ*) with probability density function:

(1) $$f(\sigma )=g(s,r)-\mu -(\sigma_{parent}*0.1),$$

where *g*(*s, r*) is the inverse gamma distribution with *s* = 3 and *r* = 0.3 representing the shape and rate parameter, respectively. μ is the expected value of *g*(*s, r*) and subtracted from *g*(*s, r*) to center the distribution *g* around zero resulting in about equal chances for decreasing and increasing the affinity of mutated subclones. We used the gamma distribution because it is right skewed and, therefore, allows for a small chance for making larger affinity improvements representing key mutations (36, 38). We do not distinguish between one or multiple affinity changing mutations. To account for the fact that mutations in higher-affinity subclones have less chance to further improve affinity we shift distribution *f* to the left as a function of the parent cell affinity (Figure S2 in Supplementary Material). The distribution shape and rate parameters (3 and 0.3) and the affinity shift (0.1) were chosen by trial and error such as to obtain the dynamics of a typical GC.

#### 2.2.3 Positive and Negative Selection of Subclones

Following cell division and SHM, the CBs differentiate to CCs that are programmed to undergo apoptosis (negative selection) unless they receive survival signals (positive selection) through interactions with the Ag (presented by FDCs) and Tfh cells (1). These selection mechanisms impose competition between the B-cell subclones, which are assumed to be based on their relative BCR affinities *σ_rel_* (1). CCs bind Ag to acquire their first survival signal. Subsequently, the Ag is internalized and presented to Tfh cells. Higher-affinity B cells present more Ag and, therefore, compete favorably for the limited number of Tfh cells to acquire a second survival signal. Positively selected CCs recycle to the dark zone for further rounds of division and SHM, or they differentiate into memory cells or plasma B cells.

**Figure 3:**
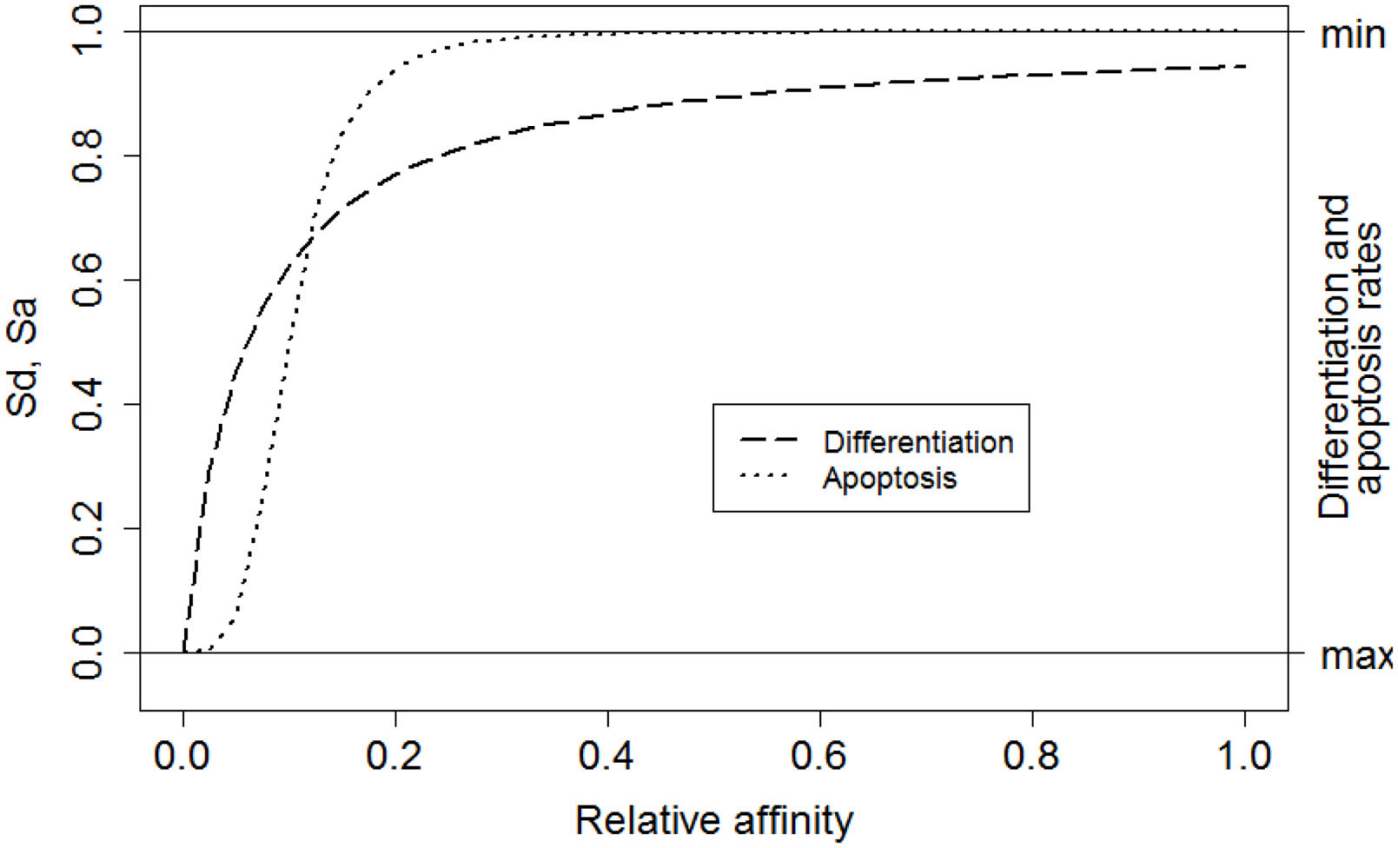
**The relative affinity of each subclone determines the magnitude of the overall survival signal**. Left axis: *S_d_* corresponds to signal affecting the CB to CC differentiation rate (dashed line). *S_a_* corresponds to the signal affecting CC apoptosis (dotted line). Right axis: effect of signal *S* on the differentiation and apoptosis rates. A high signal results in low differentiation and apoptosis rates.

To avoid an overly complex model, Ag and Tfh survival signals are modeled with a sigmoidal function:

(2) $$S(\sigma_{rel,i})=\frac{\sigma_{rel,i}^{n}}{k^{n}+\sigma_{rel,i}^{n}},$$

where *i* denotes a subclone. This function converts relative affinities *σ_rel,i_* to a signal strength between 0 and 1. Relative affinities are obtained by scaling absolute affinities *σ* to values between 0 and 1. Signal *S* affects the CB to CC differentiation rate (*η_CB_*_→_*_CC_*) and the CC apoptosis rate (μ*_CC_*) [equations (3a) and (3b)]. Recently, it was shown that higher-affinity cells stay longer in the dark zone further facilitating their expansion and diversification resulting in less apoptosis (39). This is accommodated in our model by multiplying the CB to CC differentiation rate with (1 − *S*) resulting in a rate between 0 and its maximum value *η_CB_*_→_*_CC_* (Table 1). Similarly, a higher signal reduces the apoptosis rate. We assume that *S* does not affect these rates to the same extent and, therefore, we parameterized *S* separately for differentiation and apoptosis. We set *k* = 0.06 and *n* = 1 for differentiation (*S_d_*), and *k* = 0.1 and *n* = 4 for apoptosis (*S_a_*; Figure 3). The parameters *k* and *n* were chosen to obtain a typical GC response that attains a maximum number of cells during the first phase of the GCR. During our simulation the emergence of new subclones with higher absolute affinity will “push” existing subclones with lower affinities to lower relative affinities as result of the scaling and, hence, to smaller survival signals resulting in the vanishing of these subclones.

**Table 1:** **Model parameters**.

B-cell type	Proliferation rate (day*^−^*^1^)	Differentiation rate (day*^−^*^1^)	Apoptosis rate (day*^−^*^1^)
Centroblast (*CB*)	*ρ_CB_* = 4 (40–42)	*η_CB→CC_* = 6 (1)	

Centrocyte (*CC*)		*η_CC→M_* = 1 (43) *η_CC→P_* = 0.1 (43) *η_CC→CB_* = 1 (1)	μ*_CC_* = 4 (17)	

Plasma cell (*P*)			μ*_P_* = 0.25 (43)

Memory cell (*M*)			μ*_M_* = 0.01 (43)

**Other parameters**			
Capacity A = 8,000		k = 0.06, n = 1 (*S_d_*)	s = 3.0
Number of founder cells: 3		k = 0.1, n = 4 (*S_a_*)	r = 0.3
Initial affinities: 0.1, 0.3, 0.5 μM		h = 20	Affinity shift = 0.1 μM

#### 2.2.4 Ordinary Differential Equations

**Figure 4:**
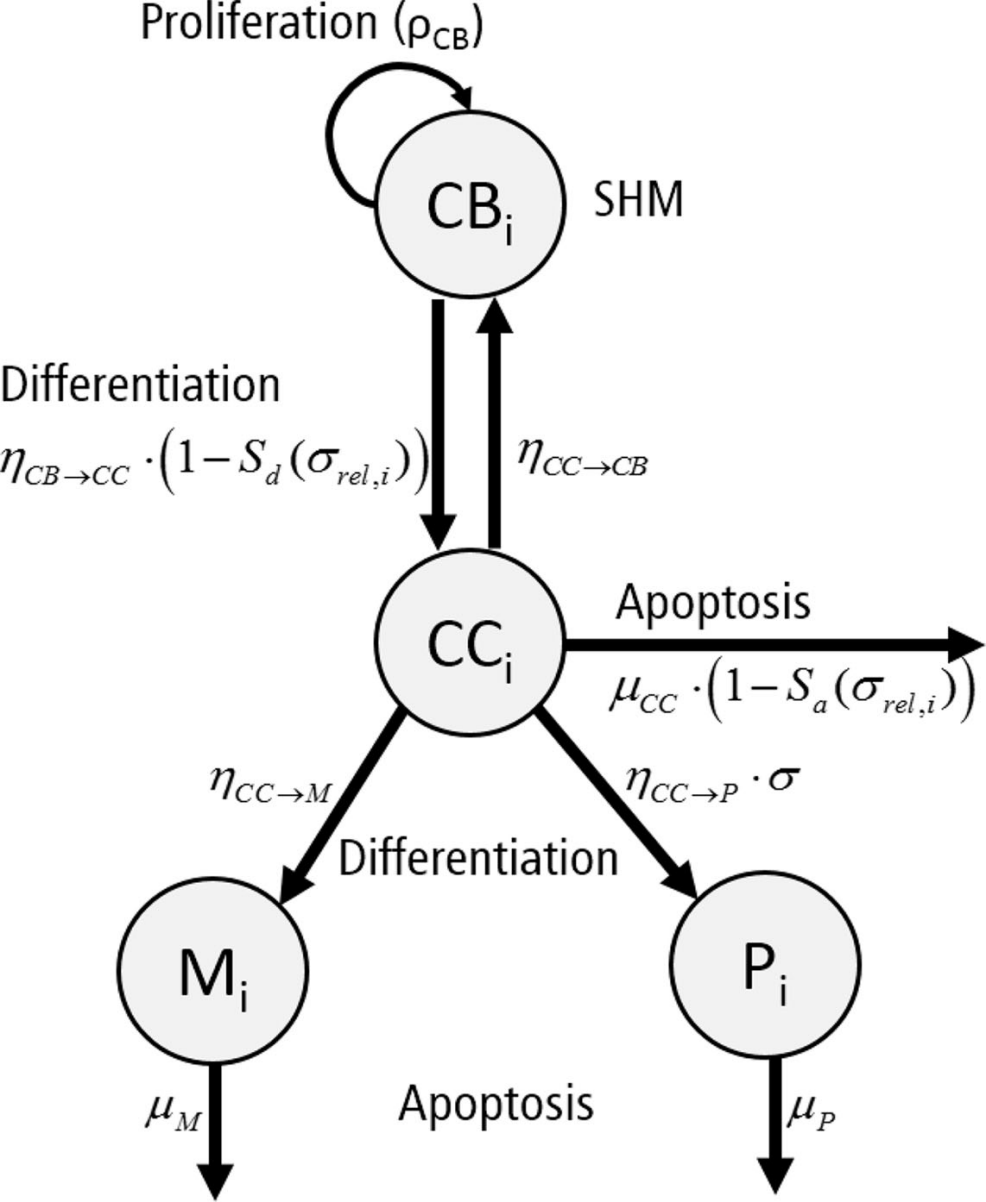
**Graphical representation of the ordinary differential equations representing a single subclone *i***. Each subclone assumes four phenotypes: centrocytes (CC), centroblasts (CB), plasma cells (P), and memory cells (M). Cells proliferate (*ρ_CB_*), differentiate (*η_CB→CC_, η_CC→CB_, η_CC→P_, η_CC→M_*), or go into apoptosis (μ*_CC_*, μ*_P_*, μ*_M_*) with indicated rates. The apoptosis rate of CCs and differentiation rate of CBs depend on signal *S_a_* and *S_d_*, respectively. Differentiation to plasma cells depends on the absolute affinity of the CCs.

Each subclone *i* assumes 4 phenotypes: centrocytes (*CC_i_*), centroblasts (*CB_i_*), memory cells (*M_i_*), and plasma cells (*P_i_*) (Figure 4). The temporal dynamics of each individual subclone is described by a set of ordinary differential equations (ODEs) representing these four phenotypes [equations (3a)–(3d)].

(3a) dCBidt=ρCB⋅(AhCBtotalh+Ah)⋅CBi+ηCC→CB⋅CCi−(1−Sd(σrel,i))⋅ηCB→CC⋅CBi

(3b) dCCidt=(1−Sd(σrel,i))⋅ηCB→CC⋅CBi−ηCC→CB⋅CCi−(1−Sa(σrel,i))⋅μCC⋅CCi−ηCC→M⋅CCi−ηCC→P⋅σi⋅CCi

(3c) dMidt=ηCC→M⋅CCi−μM⋅Mi

(3d) dPidt=ηCC→P⋅σi⋅CCi−μP⋅Pi

To allow the GC to grow to a sufficient number of cells during monoclonal expansion, the signal *S*_{_*_d,a_*_}_ is set to 0.9 for the first 4 days of the simulation to reduce differentiation of CBs to CCs and apoptosis of these initial CCs. The CB equation includes a density-dependent expansion term defining non-specific resource competition between the B cells, reducing their proliferation rate if the number of cells approaches *A*. The CC apoptosis rate and the CB to CC differentiation rate are multiplied by $\left(1-S_{\left\{d,a\right\}}(\sigma_{rel_{i}})\right)$ for reasons explained above. Plasma cell differentiation depends on the absolute affinity *σ_i_* to reduce their production at earlier stages of the GCR. During the simulation we calculate the differential equations for periods of 6 h (the duration of one CB division). After each division we impose SHM and update the population of subclones as described above. For each non-lethal SHM, a new subclone and an additional set of four ODEs are created. The CB cell count for new subclones is set to one, while the corresponding cell counts for the CCs, memory cells, and plasma cells are set to zero. The CB cell count of the parent subclone is reduced by one. If the sum of CC and CB counts for subclone *i* is less than 0.1 cell, we remove the subclone and corresponding equations from the system. Since SHM is a stochastic process that affects the subclone population and their (relative) affinities, we repeated the simulations 15 times with the same initial conditions (three founder B cells with initial affinities 0.1, 0.3, and 0.5).

#### 2.2.5 Model Parameters

Parameter values for proliferation, differentiation, and apoptosis were obtained from literature (Table 1). Parameters *A* and *h* were chosen to limit the maximum size of the GC. Values for parameters *k, n, s, r*, and affinity shift were acquired by trail and error aiming to produce a typical GC response with a peak of at least 10,000 cells during the first phase of the GCR. There are very limited (quantitative) data describing the GC response. We are not aware of any data obtained from human samples describing the dynamics of GC volume (number of cells) during the GCR. Consequently, the precise timing and magnitude of the maximum GC response, its decay, the biological variation of this response across samples and organisms, and the factors affecting this response remain to be established. The canonical GC response has, for example, been observed by tracking follicle center volume as fraction of total splenic volume in mice (41) or as fraction of the total volume of the GC in rat (44), which may be used as GC cell count substitutes. These volumes showed a peak during the first phase of the GC. Such measurements have been used previously to validate a GC model (20). However, other studies showed that there might not exist a typical GC in terms of size (45), and that GCs in a single immune response might not be synchronized (8). The lack of precise quantitative data, current uncertainties in GC dynamics, and our decision not the model GC termination limits the possibilities and value of a compute-intensive parameter inference strategy to obtain values for the aforementioned parameters. However, instead of our trial and error approach, Approximate Bayesian Computation algorithms (46), MEANS (47), or other methods may be used to fit parameters on complex stochastic models such as ours.

### 2.3 Identification of Expanded Subclones

To determine a threshold that identifies expanded subclones we follow an approach that is similar to the method we applied in our previous repertoire sequencing studies, e.g., Ref. (3, 10). First, a histogram of counts *c* (cell counts for simulated data and read counts for experimental data) for all (un)expanded subclones is constructed to reflect their cell/read count frequencies *F*(*c*) (Figure S3 in Supplementary Material). In general, subclones with low counts (e.g., *c* = 1) occur much more frequently (high *F*) than subclones with high count (e.g., *c* = 100). Next, we define *T* as lowest count *c* for which *F*(*c*) = 0. That is, no subclones with *c* cells/reads are observed. We assume that *F*(*c* ≥ *T*) = 0 for the underlying but unknown null distribution of unexpanded subclones. We define subclones with *c* > *T* (*F*(*c*) ≥ 1) to be expanded. That is, subclones observed with cell/read counts *c* > *T* are larger than expected based on the distribution of unexpanded subclones. The threshold *T* is stringent but could be relaxed by defining the threshold *T* as the lowest count *c* for which *F*(*T*) < *p*, with *p* ≥ 1.

The expansion threshold *T* was estimated for each individual simulation. We assumed that repertoire sequencing experiments measure mainly CCs since CBs do not, or at very low levels, express BCRs. Consequently, for the simulated data we determine threshold *T* from CC cell counts only. CC cell counts were taken from the last time point of the simulation.

### 2.4 Comparison of Simulated and Experimental Data

We qualitatively compare subclone cell counts from our simulations to read counts from a single sample repertoire sequencing experiment. Since our computational model does not explicitly represents the BCR as a nucleotide (or protein) sequence we do not consider multiple (back) mutations occurring at previously mutated positions. Consequently, the number of different mutations and, hence, subclones in our simulation is slightly overestimated.

Each unique nucleotide read obtained from repertoire sequencing (RNAseq) can be considered as a unique subclone representing a set of mutations acquired during affinity maturation. Statistics calculated for these subclones can be compared to statistics calculated for the nucleotide-level subclones generated in our simulations. Alternatively, we can define subclones measured in the sample at the peptide level as having unique combination of V and J segments (determined by alignment) together with a unique CDR3. The peptide level definition allows to compare the statistics from the experimental data to the peptide-level simulations. In contrast to subclones analyzed at the nucleotide-level, this definition considers any mutations in the CDR3 for the experimental data and any affinity changing mutation in CDR1,2,3 for the simulated data.

Repertoire sequencing experiments performed on tissue (e.g., lymph node) generally results in a set of subclones from multiple GCs and, most likely, from different Ag responses, while in our simulation we generate subclones from a single GCR initiated by three founder clones. We account for this by selecting subclones corresponding to three lineages from sample LN25. We first map all reads against reference sequences extracted from the IMGT database to determine their V and J segments. Subsequently, combinations of V and J are counted, and reads corresponding to the three most abundant V–J combinations (V3.7-J4, V3.74-J4, and V3.23-J4) are selected. The resulting three groups of reads still comprise subclones from multiple lineages. Therefore, we subsequently aligned all pairs of reads within each V–J group to determine the number of nucleotide differences (mutations) between them. Each pair of reads with two or fewer differences is connected to form clusters of subclones that are assumed to belong to the same lineage. Finally, the largest cluster (lineage) for each V–J combination was selected. The same procedure was followed at the peptide level. Since these three clusters did not include the most abundant subclone, we also selected the second largest cluster from the V3.7-J4 subclones. The results of this procedure are shown in Table 2. Note that the number of differences between pairs of not connected reads within a cluster (lineage) may be larger than 2. These clusters of reads could in principle be subjected to further phylogenetic analysis to determine a lineage tree establishing their relationships (48).

**Table 2:** **Selected B-cell subclones from sample LN25**.

Subclone	Total		Largest cluster (lineage)	
	Subclones	Reads	Subclones	Reads
V3.7-J4 (nt)	171	334	84	232
V3.74-J4 (nt)	125	249	23	56
V3.23-J4 (nt)	37	60	5	12

Total (nt)	333	643	112	300

V3.7-J4 (pep)	89	606	19	36
V3.74-J4 (pep)	97	519	9	14
V3.23-J4 (pep)	76	193	7	12

Total (pep)	262	1,318	35	62

			**Second largest cluster (lineage)**	

V3.7-J4 (pep)	89	606	9	417

*V and J nomenclature follow IGMT (23, 34). Subclones are defined as unique nucleotide sequences (nt) or as peptides (pep) with unique V and J assignment and a unique CDR3 sequence. For each V–J family, the number of subclones and corresponding number of sequence reads are shown. The selected clusters for the given V–J segments correspond to the largest cluster of subclones having ≤2 differences at nucleotide or peptide level. For V3.7-J4, the second largest cluster, which contains the most abundant subclone, is also included*.

## 3 Results

**Figure 5:**
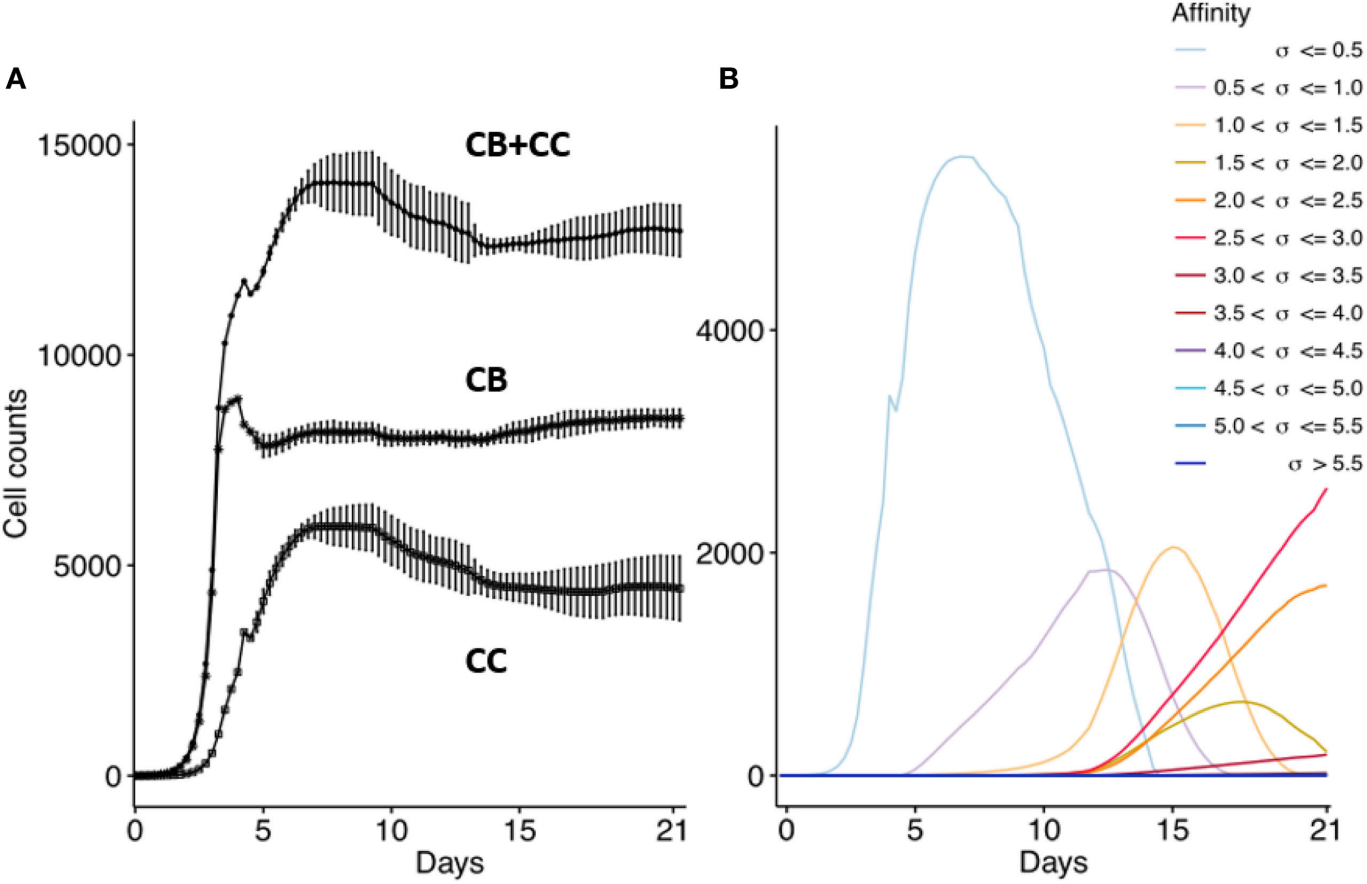
**Overall GC dynamics emerging from the model**. CB and CC with cell counts >0 are plotted. **(A)** Dynamics of CB and CC cell counts during the GCR. Top curve shows the total cell count. Each point represents the average cell count of 15 simulations at time intervals of 6 h (1 CB division). The vertical lines denote the SDs. **(B)** Evolution of absolute affinities during the GCR. Each colored line corresponds to an affinity class for which we summed the cell counts of the corresponding subclones.

First, we confirm that the computational model produces the dynamics of a typical GC response that currently is not very well defined as discussed in the method section. We performed 15 repeated simulations with subclones defined at the nucleotide level. In agreement with previous work, the GC response peaks around day 8 (Figure 5A) (41, 44, 45). The size of the GC reaches approximately 14,000 cells, which is in agreement with estimations from histological sections of two GCs (49). The CB to CC ratio (not shown) after day 8 remains between 1.4 and 2.0 and is in agreement with data obtained from intravital microscopy (50). The maximum number of SHMs in subclones emerging from our simulation ranges from 4 (day 10) to 11 (day 21) and is in good agreement with the 9 somatic mutations found in a single Ab after affinity maturation (51); with the 8 to 18 mutations found in an analysis of BCR sequences obtained from cells from GC sections derived from human lymph nodes (49); and with the 4 to 9 mutations observed in B cells from single GCs obtained from mice lymph nodes (52). Monoclonal expansion of the 3 founder cells results in many low-affinity subclones at the initial GC stage, but gradually higher-affinity clones start to appear and outcompete lower affinity subclones. As expected from affinity maturation, and in agreement with other computational models [e.g., Ref. (17, 20)], the subclone population evolves to higher affinities (Figure 5B). The drop in the CB cell count after day 4 is caused by the initiation of SHM and the subsequent differentiation to CCs that may go into apoptosis. Since we do not model GC shutdown, the cell counts remain relatively stable after 14 days. These results show that our computational model adequately captures the dynamics of a typical GCR.

### 3.1 Subclonal Diversity

**Figure 6:**
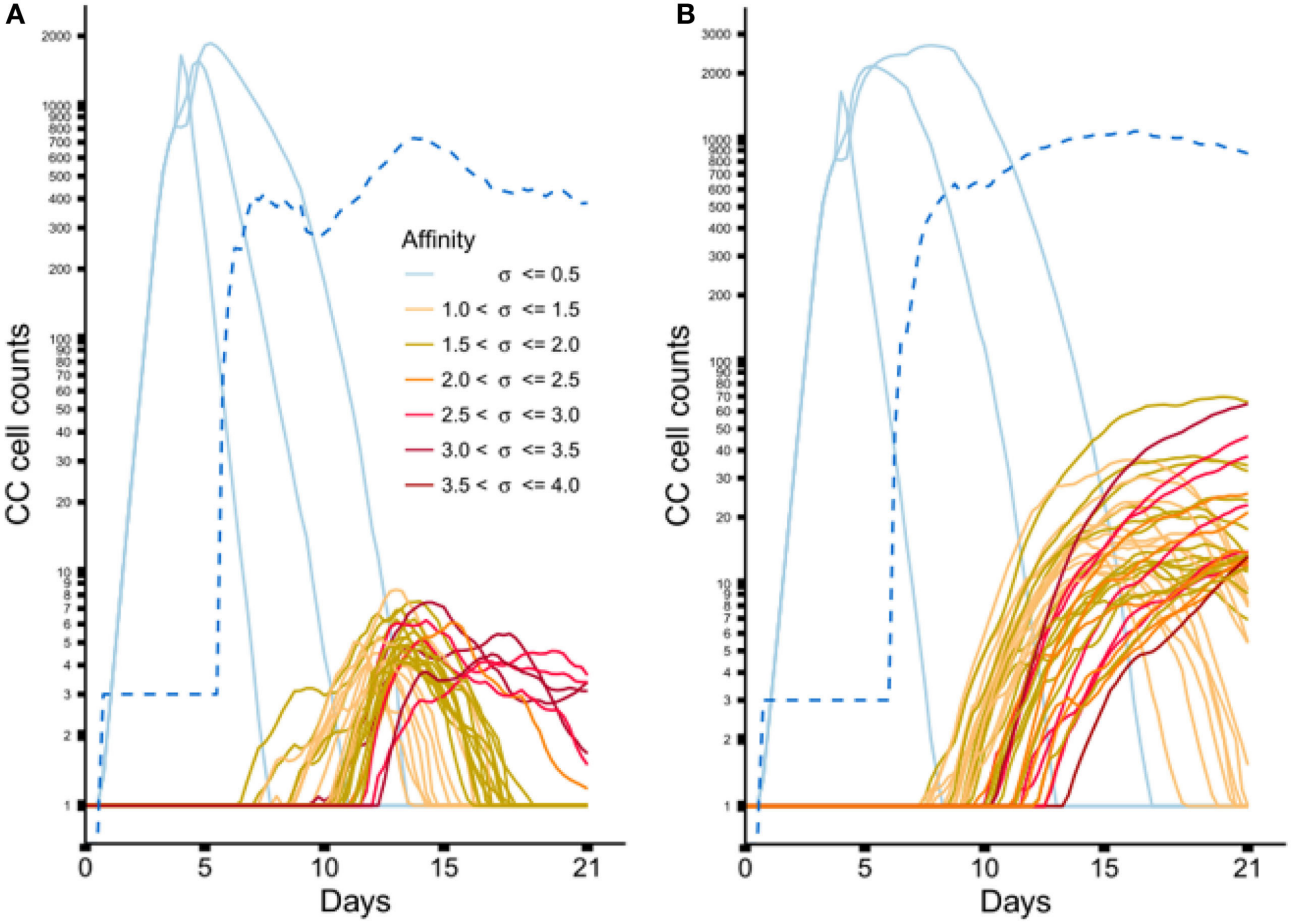
**Dynamics of individual subclones from a representative simulations**. **(A)** Subclones defined at the nucleotide level. **(B)** Subclones defined at the peptide level. Only subclones with **(A)** CC cell counts ≥4 and **(B)** CC cell counts ≥11 at any time point are shown. During the course of the GCR, new subclones of higher affinity emerge (indicated by the coloring scheme). The light blue lines represent the 3 founder subclones of low affinity. The dotted blue line shows the number of unique subclones.

Figure 6 shows the dynamics of individual subclones during the GCR at the nucleotide and peptide level. Initially, 3 founder clones expand monoclonally until day 4 after which SHM is initiated and new subclones with higher affinities start to be produced. The three low-affinity founder subclones reach high cell counts since, during monoclonal expansion, no lethal SHM occurs and *S*_{_*_d,a_*_}_ assumes a large value (0.9) resulting in a very low rate of CB differentiation and CC apoptosis. New (higher-affinity) subclones realize much lower cell counts because they start as single proliferating cells but are also reduced in count due to new mutation events and apoptosis as a result of competition with higher-affinity subclones. Interestingly, although the population of subclones evolves to higher affinities (Figure 5B) there is neither a single nor a small set of subclones that dominates this population during the later stages of the GCR. In fact, the number of unique subclones (Figure 6A) remains around 550 during the second half of the GCR.

From sample LN25 we identified 112 nucleotide-level defined subclones (i.e., unique sequence reads) corresponding to 300 reads in the three largest lineages (Table 2). Since multiple sequence reads may originate from a single B-cell it is not possible to scale these numbers to 14,000 GC cells but obviously 300 reads do not represent this many GC cells. Therefore, these 112 subclones underestimate the true number of subclones in a single GC. Although this number does not provide a validation for the 550 subclones observed in our simulations, it does show that the diversity of subclones in the experiment and the simulations is high. Using multiphoton microscopy and sequencing, it was recently shown that efficient affinity maturation can occur without homogenizing selection, and that loss of clonal diversity during the GCR varies widely from one GC to the other (52). Note that when comparing Figure 6A (nucleotide level) to Figure 6B (peptide level), the overall dynamic behavior is similar but the cell counts of higher-affinity peptide-level subclones are about five times larger. An increase in cell count is expected since, in this scenario, neutral and synonymous somatic mutations do not result in new subclones and, hence, no reduction of cell counts occurs. The number of unique subclones is still in the same order of magnitude as the previous simulation but counterintuitively increased compared to previous situation since a decrease is expected due to the fewer mutations imposed on these subclones. The observed increase is, however, a result of plotting and summing only the subclones with CC cell counts ≥1. Including cell counts <1 shows that the number of subclones indeed decreases (data not shown).

### 3.2 Subclonal Expansion

**Figure 7:**
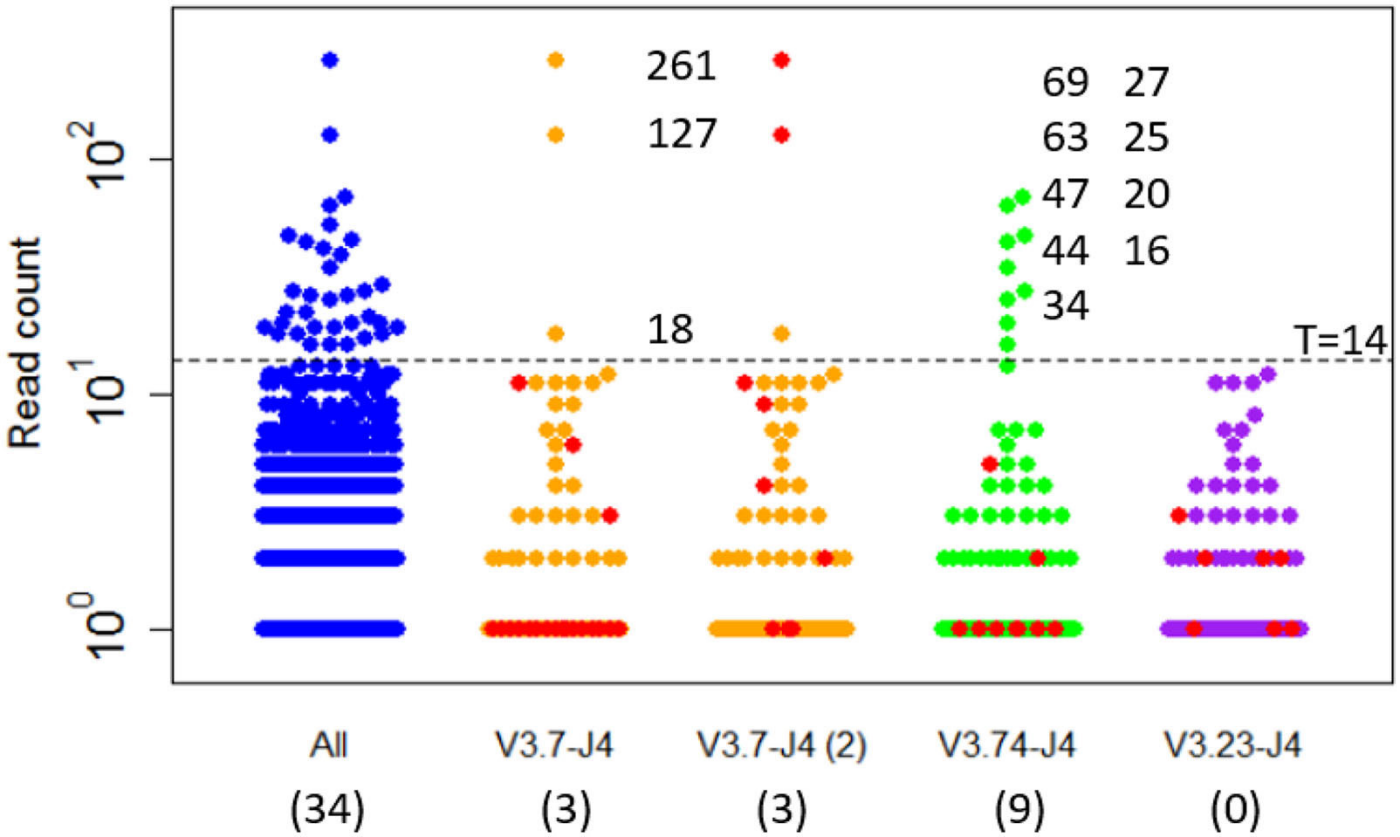
**Subclones measured in a lymph node sample (LN25) from a healthy individual**. The blue points show the read counts for all 4,454 subclones measured in this sample (34 expanded subclones). The expansion threshold (*T* = 14) is determined from the all LN25 subclones and indicated by the dashed line. Subclones of the three most abundant V–J combinations are shown in orange, green, and purple. The red dots indicate the subclones of the largest clusters and, for V3.7-J4, also the second largest cluster. Read counts of the expanded subclones are shown. The numbers in the parenthesis show the number of expanded subclones in the selected V–J subsets.

Expanded subclones are derived from experimental data on the basis of their peptide-level definition and relative abundance. Basically, this definition neglects any mutation in the V and J region and any synonymous mutations in the CDR3. We identified expanded subclones from the experimental data (Figure 7). First, the expansion threshold was determined using all subclones from the LN25 sample resulting in 34 expanded subclones. Using this threshold (*T* = 14), a total of 3 and 9 subclones from the V3.7-J4 and V3.23-J4 subclones, respectively, are expanded. For each V–J family, Figure 7 also shows the subclones corresponding to the largest cluster (read counts ranging from 1 to 11), and for V3.7-J4, the subclones corresponding to the second largest cluster (read counts ranging from 1 to 261). This shows that subclones within a B-cell lineage may exhibit a wide range of read counts, which is in agreement with our simulated data. It also shows that the most abundant subclones do not necessarily belong to the largest cluster within a V–J family.

The clonal size (number of reads of a subclone divided by total number of reads) of the expanded LN25 subclones varies from 0.2 to 3.4%. Together, these represent 0.8% (34 out of 4,454) of all subclones. This is similar to the amount of expansion found in one of our previous studies where clonal sizes ≥0.5% were found to represent expanded subclones representing 0.3 and 1.9% of the subclones in peripheral blood and synovial tissue of RA patients, respectively (10). Since our computational model does not explicitly consider V and J segments, and because we cannot distinguish CDR3 from CDR1 and CDR2 mutations, we cannot group subclones resulting from our simulation in a way similar to the experimental data. However, by neglecting neutral and silent FWR/CDR mutations we can simulate subclones at the peptide level. The resulting subclones differ only in their CDR regions. The expanded peptide-level subclones in our simulation represent clonal sizes ranging from 0.3 to 8.7% representing 0.3 to 1.0% of the subclones. This degree of expansion is in the same order of magnitude as expansion observed in our experimental data.

### 3.3 BCR Affinity of (Un)Expanded Subclones

**Figure 8:**
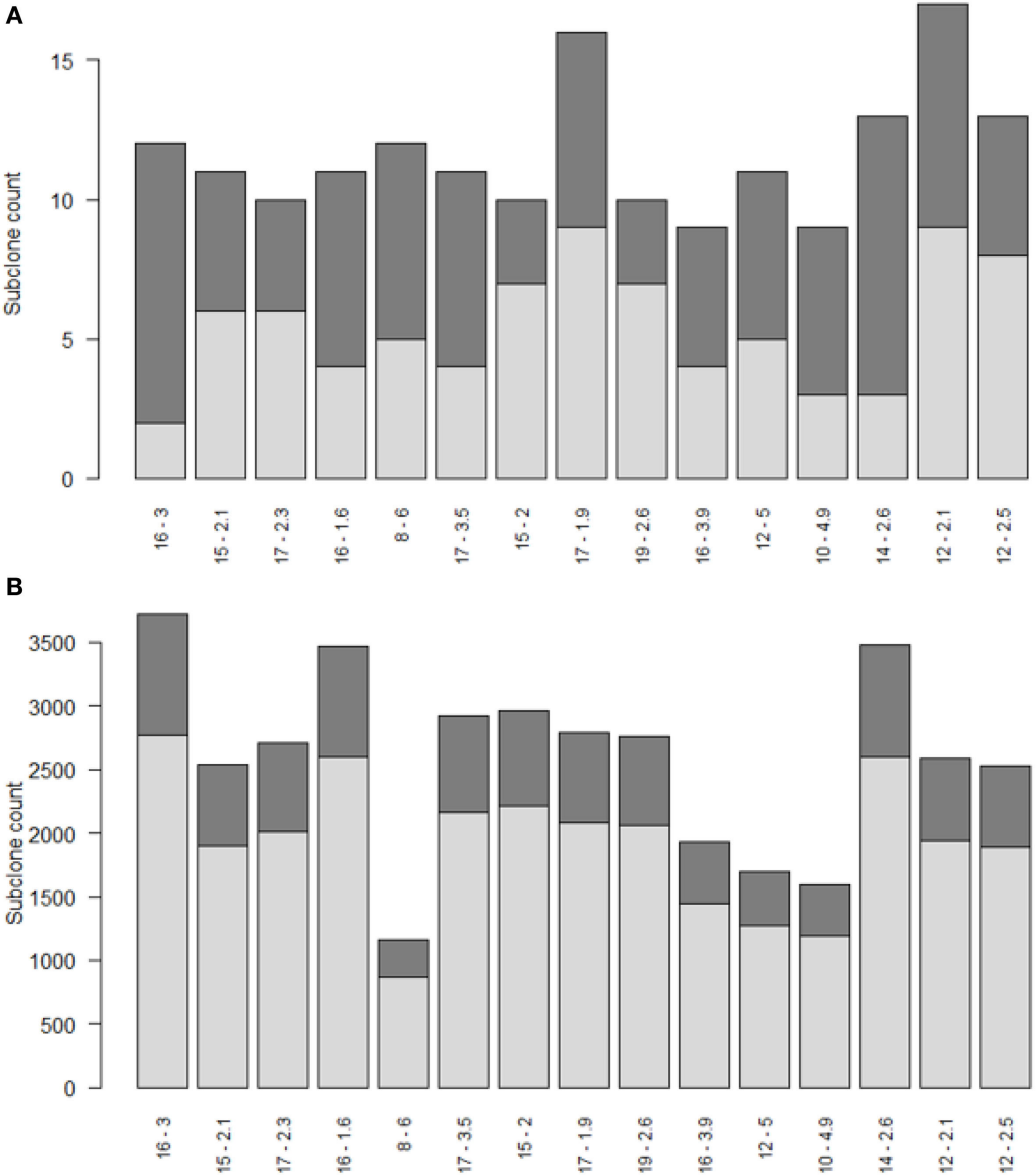
**Numbers of high (dark gray) and low (light gray) affinity subclones among expanded (A) and unexpanded (B) subclones in 15 simulations (x-axis)**. Subclones were defined at the peptide level. There are many more unexpanded subclones compared to expanded subclones. Subclones with CC cell counts >0 were included. The numbers at the x-axis denote the thresholds for expansion (*T*) and absolute affinity (75th percentile).

**Figure 9:**
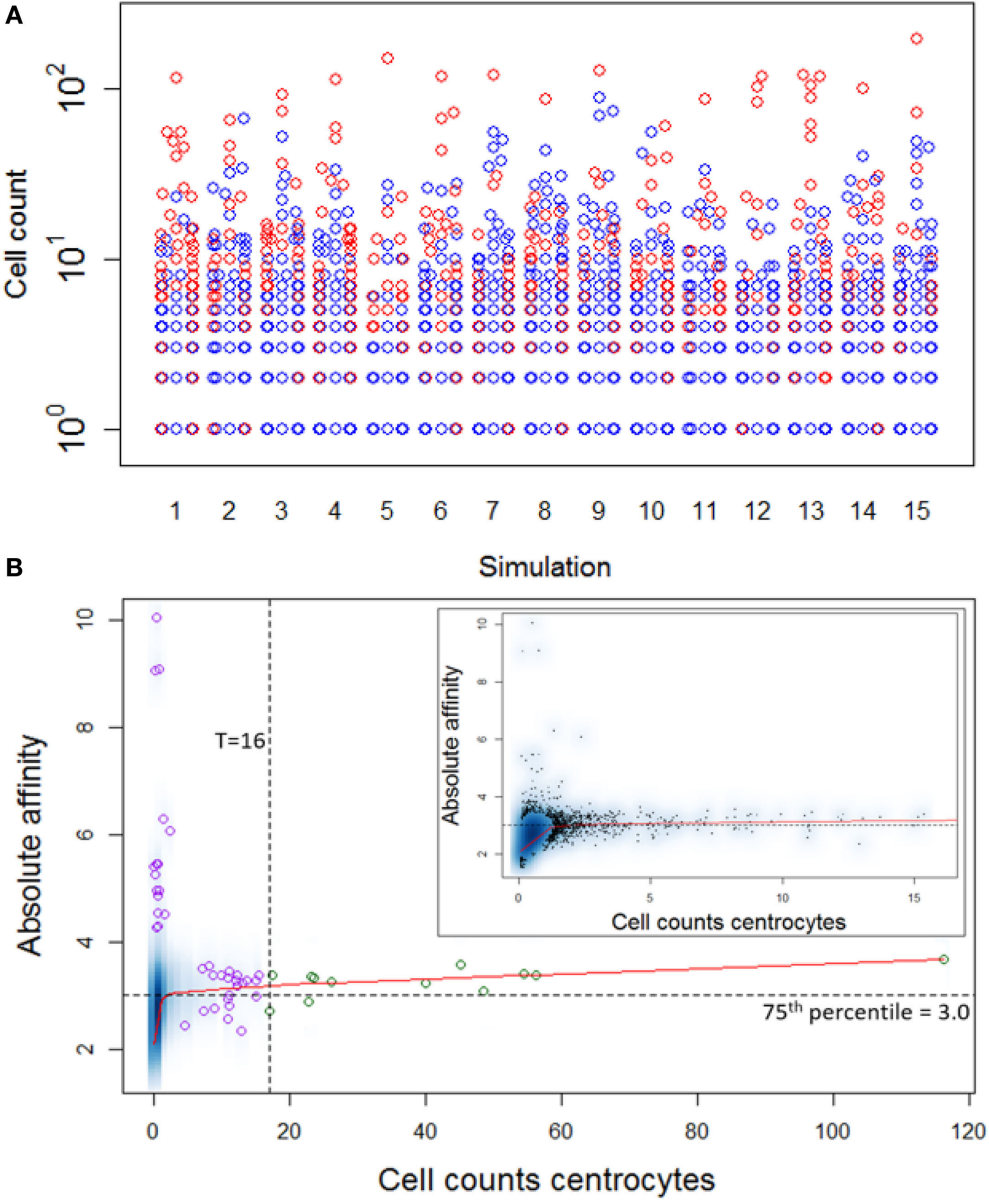
**(A)** Distribution of high-affinity subclones (red) among all subclones for 15 simulations. **(B)** Density plot of CC cell counts and absolute affinity for simulation 1. Inset shows only the low abundant subclones. Data points show a selection of subclones imposed on the density plot. Green points denote the expanded subclones. Purple points indicate a selection of low abundant subclones. The red line shows a lowess regression to indicate the overall relation between abundance and affinity.

Repertoire sequencing provides only information about the relative abundance of B-cell subclones in a sample. In contrast, our computational model provides also information about the (relative) affinity of each subclone, which we use to gain insight in the affinity distributions among expanded and unexpanded subclones. High absolute affinity was defined by setting a threshold at the 75th percentile of absolute affinities of all subclones produced during the course of the GCR (range 1.53–10.6; 75th percentile is 3.00). Figure 8 shows the number of high- and low-affinity subclones among (un)expanded subclones for each of the 15 simulations. In these simulations we defined the subclones at the peptide level. In these 15 simulations, the percentage of low-affinity subclones among high-abundant subclones varies from 17 to 70%. The percentage of high-affinity subclones among the low abundant subclones is about 25% in each of the simulations. In 14 out of 15 simulations, the affinity of most abundant subclones belongs to the highest 25% of affinities (Figure 9A), but the most abundant subclones never correspond to the highest affinity subclone (Figure 9B). Figure 9B shows that the affinity tends to increase with subclone abundance (spearman rank correlation is 0.6) but that the largest affinities correspond to the low abundant subclones. Increasing the affinity threshold to 95% results in more low-affinity subclones among the expanded subclones (data not shown).

Although the affinity distribution depends on the expansion and affinity thresholds, the results demonstrate that lower affinity cells will be among the expanded subclones and *vice versa*. However, in a repertoire sequencing experiment one might not detect the very low abundant (high-affinity) subclones. The high-affinity cells in the unexpanded fraction are either new subclones that have undergone significant affinity improvement but did not yet have sufficient time to proliferate or are high-affinity subclones previously expanded but now being outcompeted by new subclones.

## 4 Discussion

The identification of autoreactive B cells is important for understanding the pathogenesis of autoimmune diseases and developing therapies that target specific B cells to improve clinical outcome. However, for many autoimmune disorders the Ags are unknown, which makes screening approaches challenging. Repertoire sequencing strategies have been developed as an alternative Ag-agnostic approach to identify autoreactive B cells while relying on the assumption that expanded B cells measured in blood or tissue are involved in the pathogenesis of the disease. B-cell subclones identified by sequencing can be cloned and functionally characterized and used to identity the autoantigen. In previous work we demonstrated that expanded clones identified by repertoire sequencing of synovium samples from RA patients point to putative autoreactive B cells (10). This potentially provides the opportunity to develop novel therapeutic approaches targeting these cells.

(Deep) Repertoire sequencing is successfully used for the identification of antigen-specific B cells involved in immune disorders or infection. Since clonal expansion is a general feature of an immune response, the selection of highly abundant subclones to identify the Ag-specific B cells has been suggested and used by several groups (10, 12–15). Selected subclones can subsequently be characterized or can facilitate the identification of the Ag (11, 53). However, repertoire sequencing itself provides no information about affinity. Therefore, we developed a computational model to investigate the relation between subclone abundance and affinity. Although our computational model was not expected to provide precise quantitative results, we showed that the fraction of low-affinity cells among expanded subclones and the fraction of high-affinity subclones among unexpanded B cells are substantial (Figure 8). There exists a moderate positive correlation between subclone abundance and affinity, but we also showed that the highest affinity subclones are of very low abundance (Figure 9). We performed a sensitivity analysis to assess the robustness of the model for changes in model parameters (see Supplementary Material). This analysis showed that changes in parameter values have mostly small to moderate effects on the output of the model but do not change the main results of the simulations. Therefore, based on our simulations, we conclude that selection of highly abundant subclones may not necessarily lead to the high(est) affinity B cells. We realize that this conclusion will need further experimental validation by simultaneous measurement of abundance and affinity although this will remain difficult for clinical samples. Evolving experimental technologies and approaches may make this less labor intensive in the future. Using a tractable immunization mouse model and a well-defined Ag might be a first step toward validation. In this case, a single cell strategy is required to sequence both the heavy and light Ig chains. Subsequently, the Igs must be cloned and expressed followed by measuring antibody–antigen binding kinetics using surface plasmon resonance (54).

However, to support the results from the simulations we analyzed a lymph node sample (LN25) to determine (i) the variability of subclone frequencies within a lineage, (ii) the number of expanded subclones, and (iii) the subclonal diversity. Although each of these analyses supported the simulation, the LN25 sample could have contained plasma cells (PCs) that express as much more RNA than GC B cells (55) thereby distorting the relationship between sequence read counts and cellular abundances. We could not verify this experimentally since no sample was left available. In addition, we did not account for possible sequencing and PCR errors using molecular barcodes or other methods (56–60). Such errors may result in (low abundant) artificial subclones that potentially affect the interpretation of the data analysis. PCs and sequencing/PCR errors can affect the variation subclone frequencies that we observe in the data, but it is unlikely that all variability is explained by these artifacts as it is an intrinsic characteristic of affinity maturation. The number of expanded subclones can be inflated by PCs, but it is unlikely that all subclones above our threshold are PCs and, hence, the percentages of expanded clones determined from the data and simulations would probably stay comparable if PCs would be removed. Sequencing/PCR errors artificially increase subclonal diversity but naively extrapolating the number of subclones observed in the selected LN25 VJ families to 14,000 GC cells in the simulation demonstrates that removing these errors would in fact bring the observed variability closer to the simulation results. Moreover, it is unlikely that the creation of these artificial low abundant subclones largely effect the frequencies of the expanded subclones from which they could have originated. Thus, we expect that sample LN25 after accounting for PCs and/or sequencing/PCR errors, would still support the simulation results. Moreover, note that we do not use the sample to directly verify the relationship between cell count and affinity, which is solely derived from the simulations. However, to further validate our simulations we selected and analyzed a public BCR repertoire data set obtained from a cervical lymph node from a chronic multiple sclerosis patient (61). In this sample no CD188+ PCs were observed. Results from this analysis are similar as those obtained from the LN25 sample and also support our simulation results (see Supplementary Material).

In addition to merely selecting highly abundant subclones from B-cell repertoires, there exist alternative selection strategies that can be used to identify the Ag-specific B cells (6). For example, it has been shown that representative Abs selected from clonal families, reconstructed by phylogenetic analysis, neutralize influenza more effectively than “singleton” Abs that use heavy-chain V(D)J and/or light-chain VJ gene segments that are not used in any other Ab in the repertoire (5). This study showed that Abs from clonal families have significantly higher affinities than did singleton antibodies. Such strategy could be combined with subclone abundance. In previous work we have shown that the identification of pathogenic subclones in RA benefits from the selection of high-abundant subclones that are present in multiple joints within a patient (10, 22). It would be interesting to determine the affinity of these overlapping subclones in comparison to high-abundant non-overlapping clones. Marcatili and coworkers (62) used BCR repertoires from a large number of CLL patients to cluster the receptors into groups with similar sequence properties that potentially can be used for prognostics. Alternatively, one can compare repertoires across patients to identify consistent Ab sequence features (63). One might be tempted to identify high-affinity clones by selecting the clone with the highest number of somatic mutations since multiple rounds of proliferation, SHM, and selection increases the overall affinity of the GC B-cell population, but such correlation between affinity and number of mutations is not observed in our simulation results (data not shown). In addition, mutation and affinity measurements the study of Tan and coworkers did also not reveal such correlation (5).

Surprisingly, our model shows that the number of unique subclones in a single GC remains remarkably constant throughout the GCR and does not evolve to a single or few high-affinity dominating subclones although the affinity of the population as a whole increases as has been shown in previous studies (17, 20). Moreover, the cell counts of individual subclones remain very low. Adding additional mechanistic detail (e.g., GC shutdown) is unlikely to change this observation. Moreover, this observation is in agreement with repertoire sequencing data and also seems in agreement with a recent study that showed that many clones may mature in parallel, and sporadic clonal bursts generates many SHM variants of a clone (52).

The parameters used in the model (Table 1) and mutation probabilities in the decision tree (Figure 2) are not all based on recent experiments and data. Moreover, we have taken parameters and other information (e.g., the typical GC response) mostly from mouse studies since human data are often not available. It might therefore be necessary to use more up-to-date experimental approaches and/or (public) data to revisit these parameters and probabilities in order to establish them with more precision, under a variety of different conditions, and for different organisms. For example, the immunogenetics community made large progress in establishing comprehensive immunoglobulin V-gene databases. Recent work based on these data showed that there exist large differences between different mouse strains, and that mouse repertoire is more germline-focused than the human repertoire suggesting that affinity maturation is less important for mouse than it is for human (64, 65). Therefore, a typical GC response for human might be different from mouse. It also suggests that our mutation probabilities that were based on limited mouse data generated prior to 1998 (18) need to be updated as part of future work.

Our model can be improved in several ways. Given the current results it would be interesting to investigate if our results hold with more detailed GC models since with the current model it is very difficult to control the amount of expansion by changing the sigmoid functions without distorting the overall GC dynamics (although this might happen also *in vivo*). It would be interesting to investigate what exactly controls selection pressures and how this affects subclonal expansion and the BCR affinity distribution. Nevertheless, as we have shown, the current magnitude of expansion observed from the model is in the same order of magnitude as observed in experimental data from LN25. However, the amount of expansion shown in sample M5 (Supplementary Material) can currently not be reproduced with the model. To allow a better comparison to the experimental data we plan to include an explicit representation of the BCR as a nucleotide sequence in our future model. This would allow to distinguish between the different CDR regions, to account for multiple (back) mutations at identical positions, and to more precisely specify subclones at both the nucleotide and protein level. In analogy to Ref. (7, 66), this would allow to explore the clonal composition and subclonal dynamics in a system where the BCR sequence with the highest Ag affinity is known and can be reached in few (key) mutations such as in the response against (4-hydroxy-3-nitrophenyl)acetyl. However, in general, the incorporation of realistic affinities in GC models will remain a challenge until these really can be measured on a large scale. Another interesting extension would include the egress of B cells to investigate the (sub)clonal composition in blood and to compare this to repertoire sequencing data obtained from blood samples. Finally, we did not model GC shutdown since its mechanism is unknown, although mechanisms have been hypothesized such as Ab feedback (67). It is difficult to predict how GC shutdown would affect the results presented in this paper since this would depend on the timing that such mechanism would affect the different cell types and how it would differentiate between subclones with different abundancy and affinity.

## Author Contributions

NV and AK designed the study. PR, AK, JG, and PK defined the model. PR performed the simulations. PR and AK analyzed the results from the simulations. P-PT developed the protocol for acquiring LN samples and provided the sample used in this study. RE conducted the repertoire sequencing experiment. PK, MD, BS, AK, and NV analyzed the experimental data. PR and AK wrote the manuscript. All authors critically read, contributed, and approved the manuscript.

## Conflict of Interest Statement

The authors declare that the research was conducted in the absence of any commercial or financial relationships that could be construed as a potential conflict of interest.
