# Supplementary material for: Computational Model Reveals Limited Correlation between Germinal Center B-Cell Subclone Abundancy and Affinity: Implications for Repertoire Sequencing
Source: Front Immunol. 2017 Mar 6;8:221. doi: 10.3389/fimmu.2017.00221 (PMC5337809; doi:10.3389/fimmu.2017.00221)
Supplement: Supplementary file 1 [file Data_Sheet_1.ZIP › data sheet 1/Supplementary Information/SensitivityAnalysis_v1.pptx]

## Slide 1
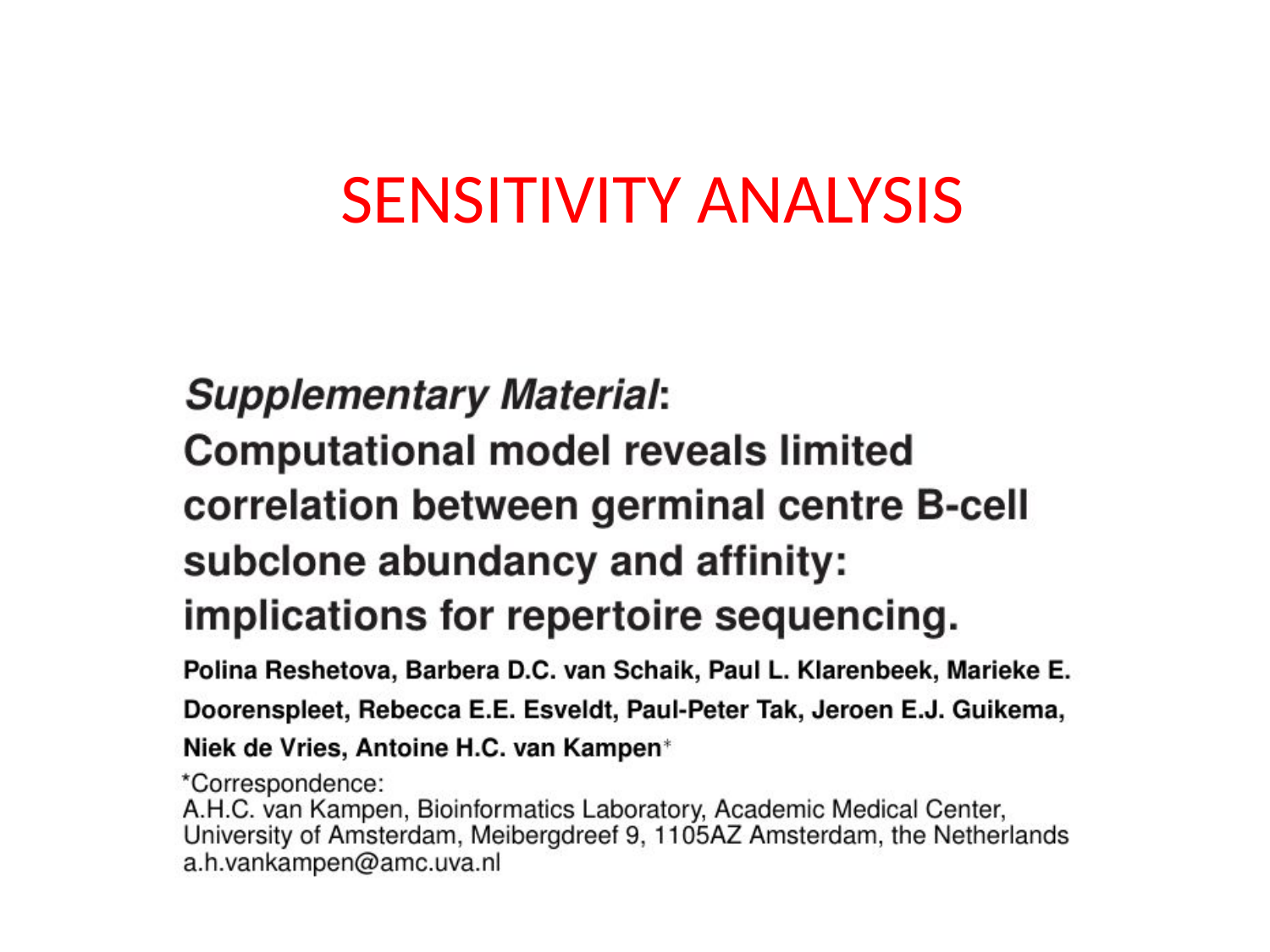

SENSITIVITY ANALYSIS

## Slide 2
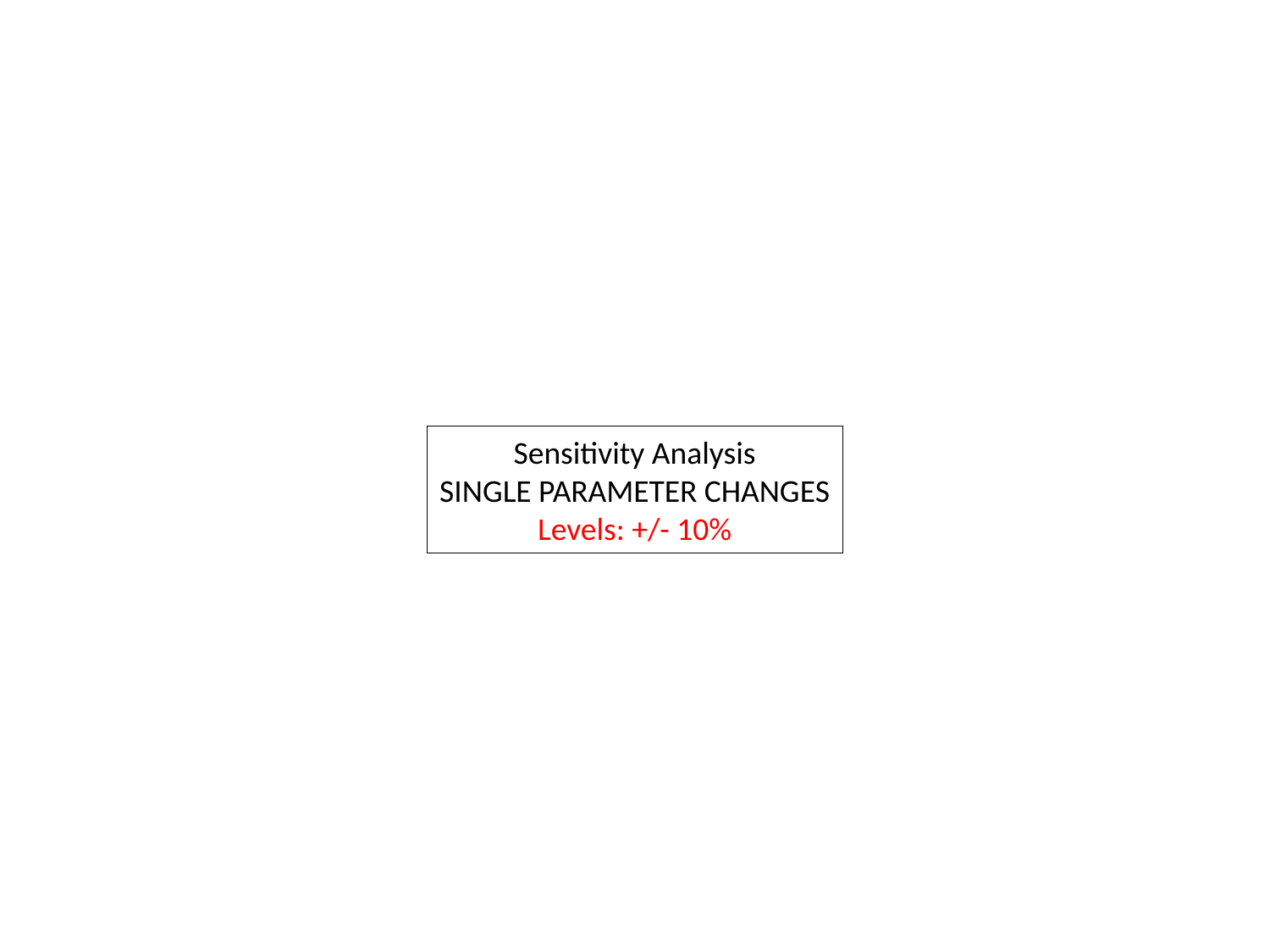

Sensitivity Analysis
SINGLE PARAMETER CHANGES
Levels: +/- 10%

## Slide 3
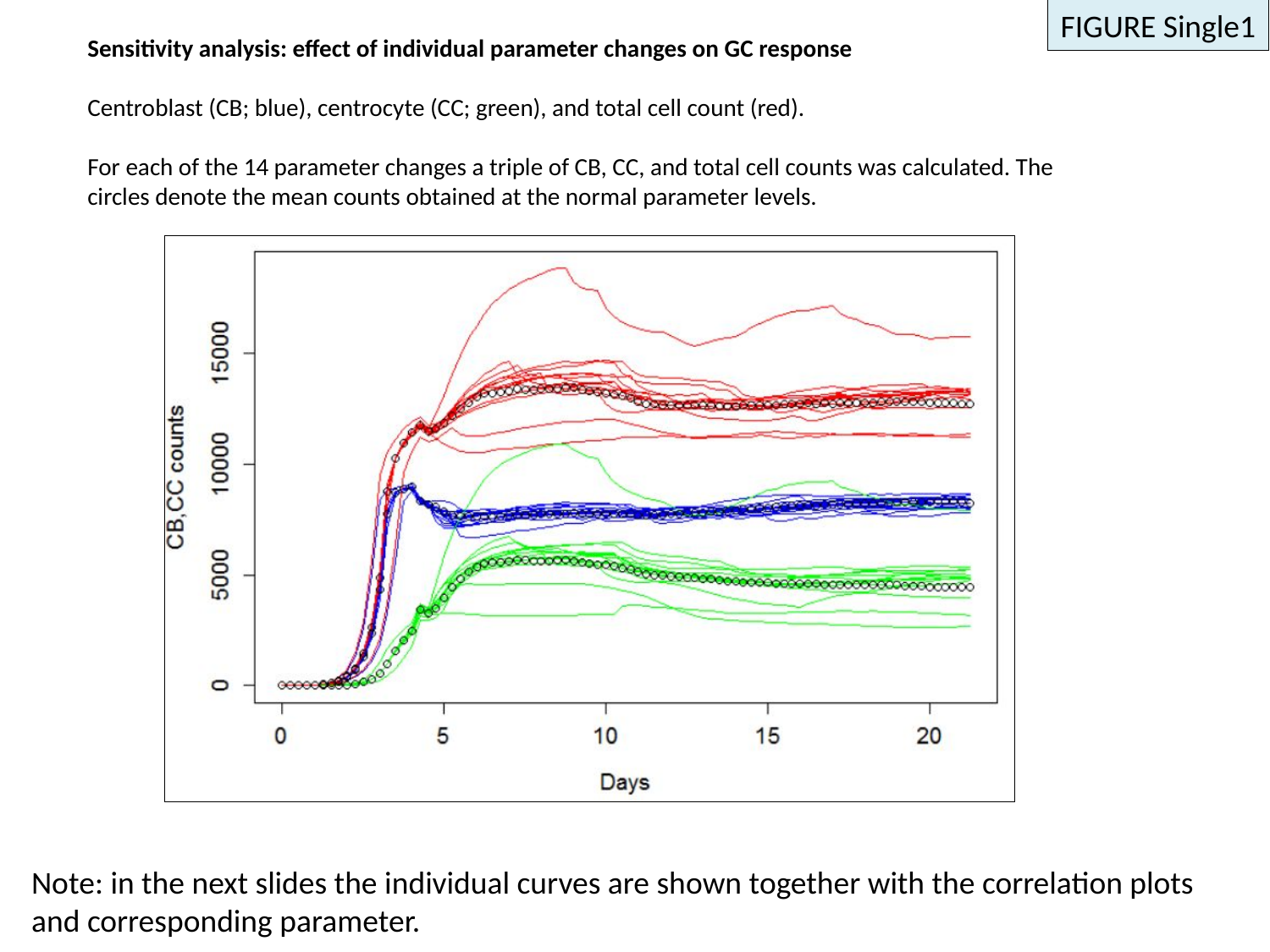

FIGURE Single1
Sensitivity analysis: effect of individual parameter changes on GC response
Centroblast (CB; blue), centrocyte (CC; green), and total cell count (red).
For each of the 14 parameter changes a triple of CB, CC, and total cell counts was calculated. The
circles denote the mean counts obtained at the normal parameter levels.
Note: in the next slides the individual curves are shown together with the correlation plots
and corresponding parameter.

## Slide 4
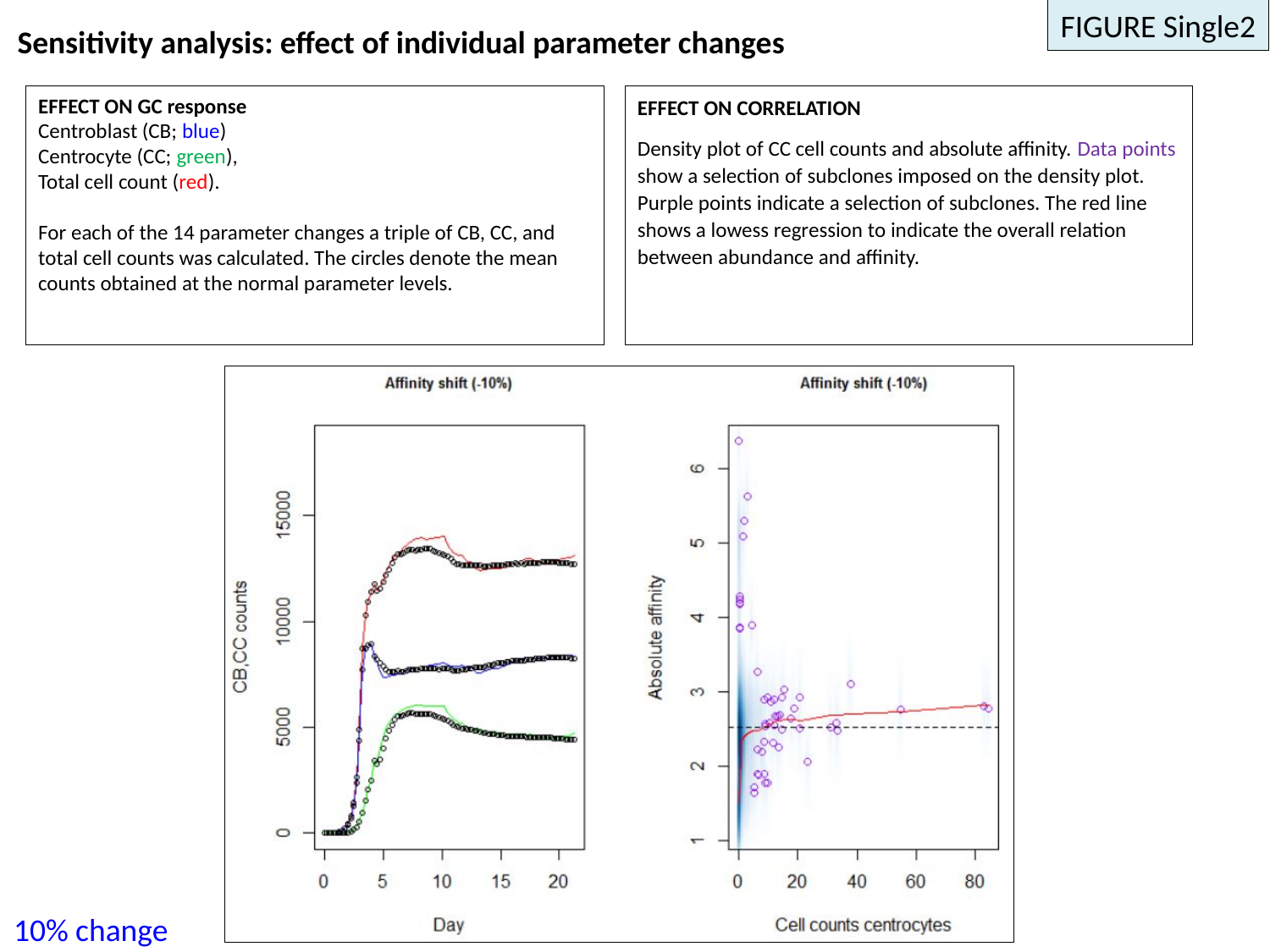

FIGURE Single2
Sensitivity analysis: effect of individual parameter changes
EFFECT ON CORRELATION
Density plot of CC cell counts and absolute affinity. Data points show a selection of subclones imposed on the density plot. Purple points indicate a selection of subclones. The red line shows a lowess regression to indicate the overall relation between abundance and affinity.
EFFECT ON GC response
Centroblast (CB; blue)
Centrocyte (CC; green),
Total cell count (red).
For each of the 14 parameter changes a triple of CB, CC, and total cell counts was calculated. The circles denote the mean counts obtained at the normal parameter levels.
10% change

## Slide 5
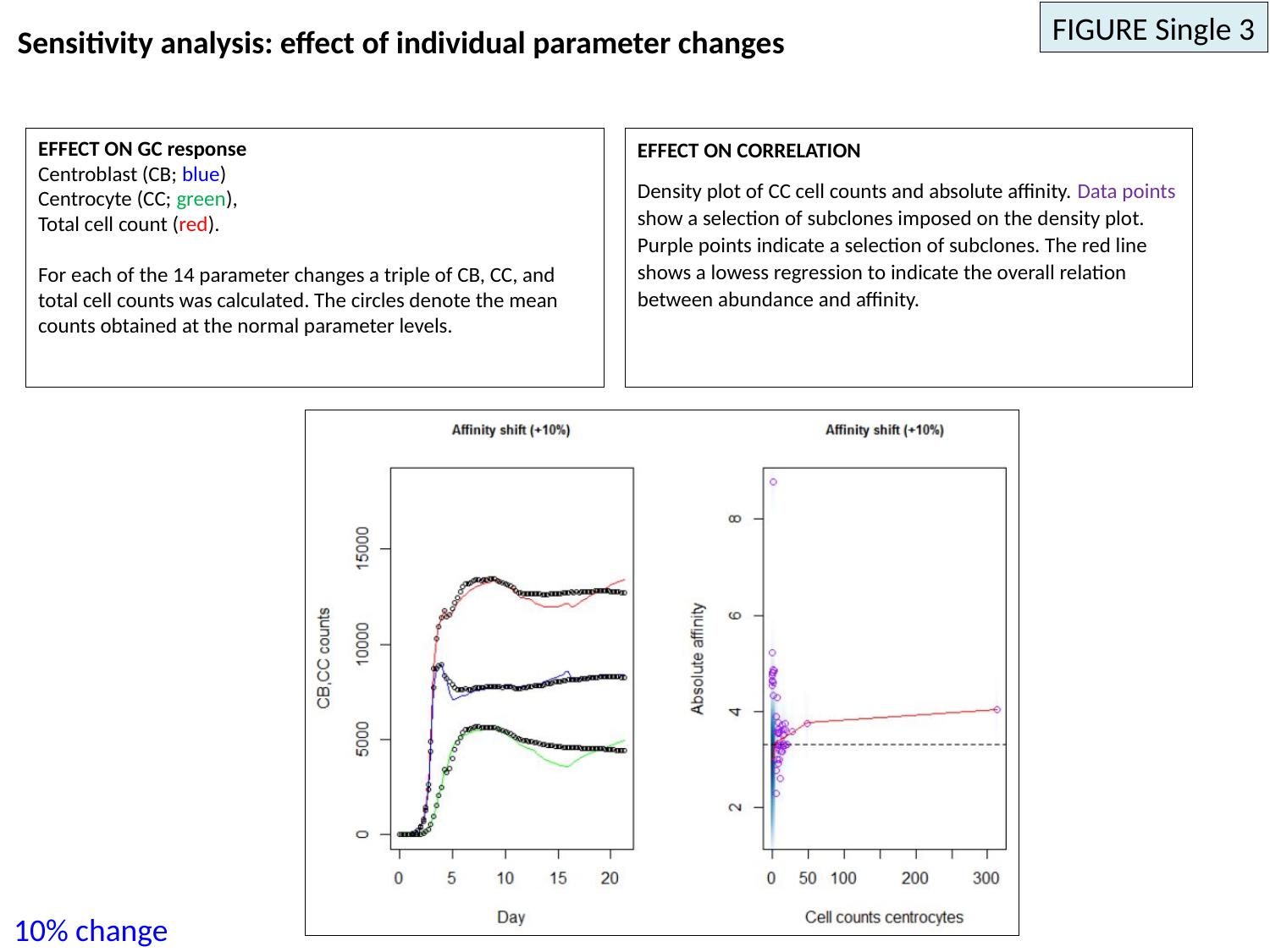

FIGURE Single 3
Sensitivity analysis: effect of individual parameter changes
EFFECT ON CORRELATION
Density plot of CC cell counts and absolute affinity. Data points show a selection of subclones imposed on the density plot. Purple points indicate a selection of subclones. The red line shows a lowess regression to indicate the overall relation between abundance and affinity.
EFFECT ON GC response
Centroblast (CB; blue)
Centrocyte (CC; green),
Total cell count (red).
For each of the 14 parameter changes a triple of CB, CC, and total cell counts was calculated. The circles denote the mean counts obtained at the normal parameter levels.
10% change

## Slide 6
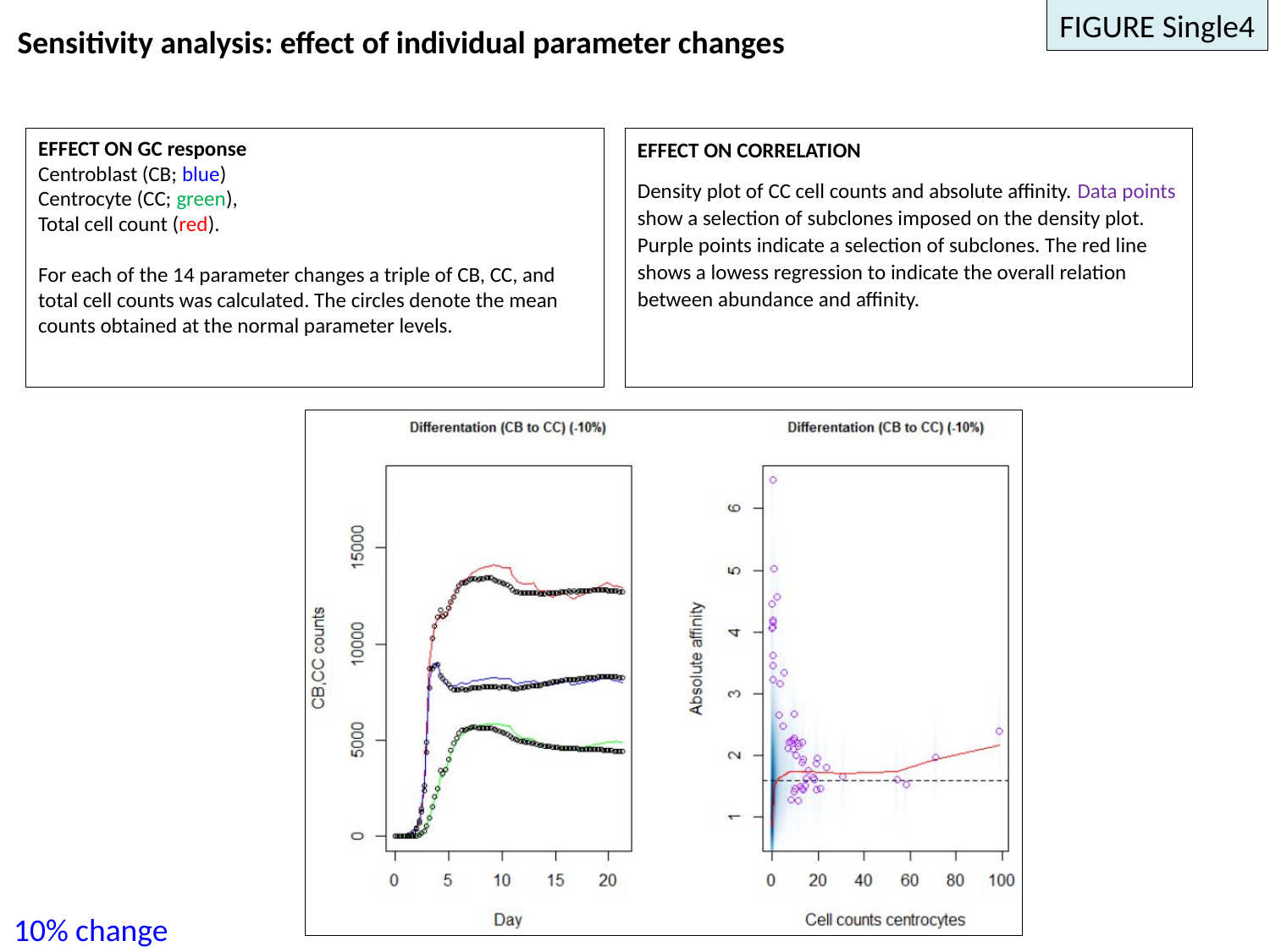

FIGURE Single4
Sensitivity analysis: effect of individual parameter changes
EFFECT ON CORRELATION
Density plot of CC cell counts and absolute affinity. Data points show a selection of subclones imposed on the density plot. Purple points indicate a selection of subclones. The red line shows a lowess regression to indicate the overall relation between abundance and affinity.
EFFECT ON GC response
Centroblast (CB; blue)
Centrocyte (CC; green),
Total cell count (red).
For each of the 14 parameter changes a triple of CB, CC, and total cell counts was calculated. The circles denote the mean counts obtained at the normal parameter levels.
10% change

## Slide 7
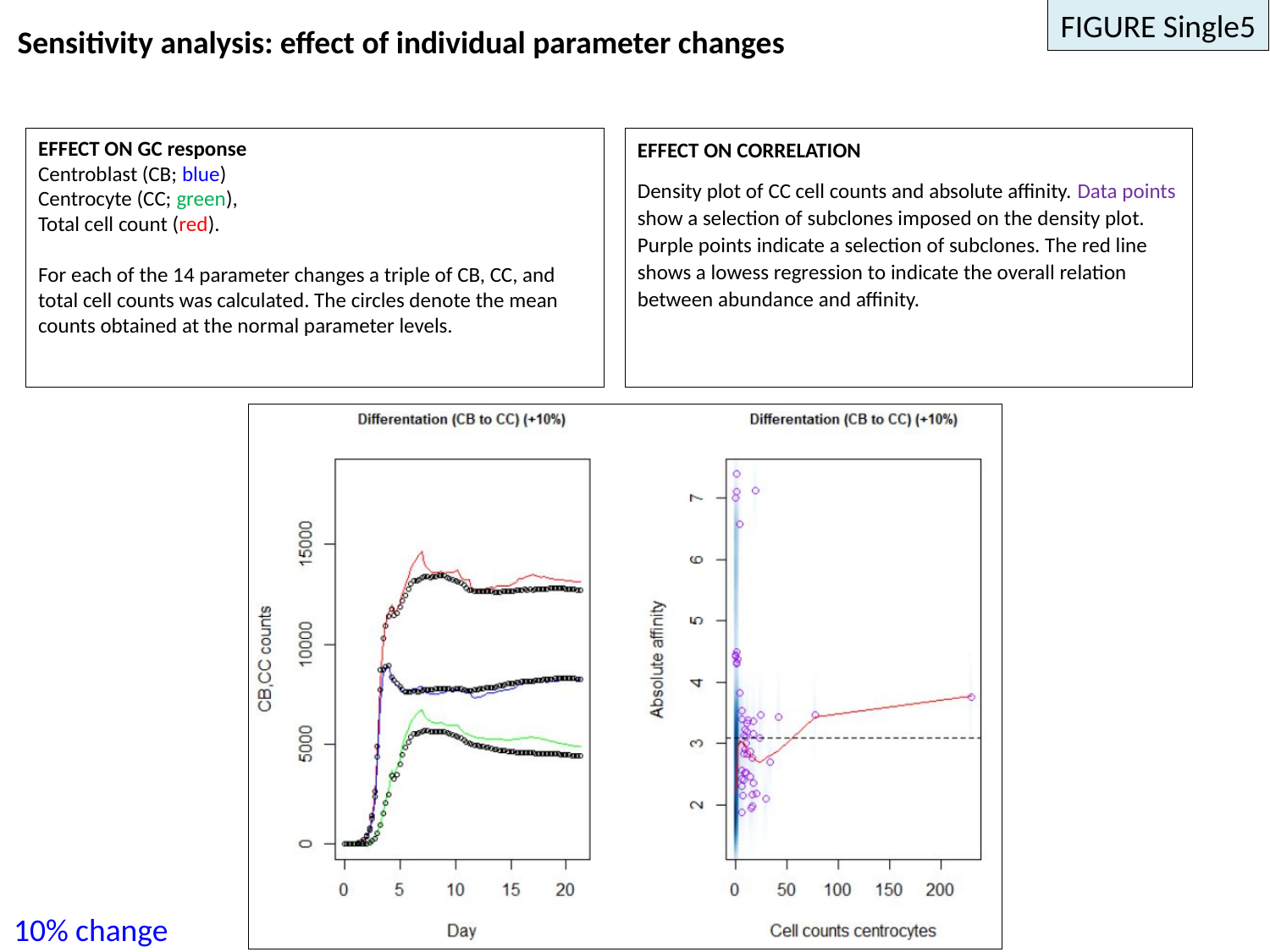

FIGURE Single5
Sensitivity analysis: effect of individual parameter changes
EFFECT ON CORRELATION
Density plot of CC cell counts and absolute affinity. Data points show a selection of subclones imposed on the density plot. Purple points indicate a selection of subclones. The red line shows a lowess regression to indicate the overall relation between abundance and affinity.
EFFECT ON GC response
Centroblast (CB; blue)
Centrocyte (CC; green),
Total cell count (red).
For each of the 14 parameter changes a triple of CB, CC, and total cell counts was calculated. The circles denote the mean counts obtained at the normal parameter levels.
10% change

## Slide 8
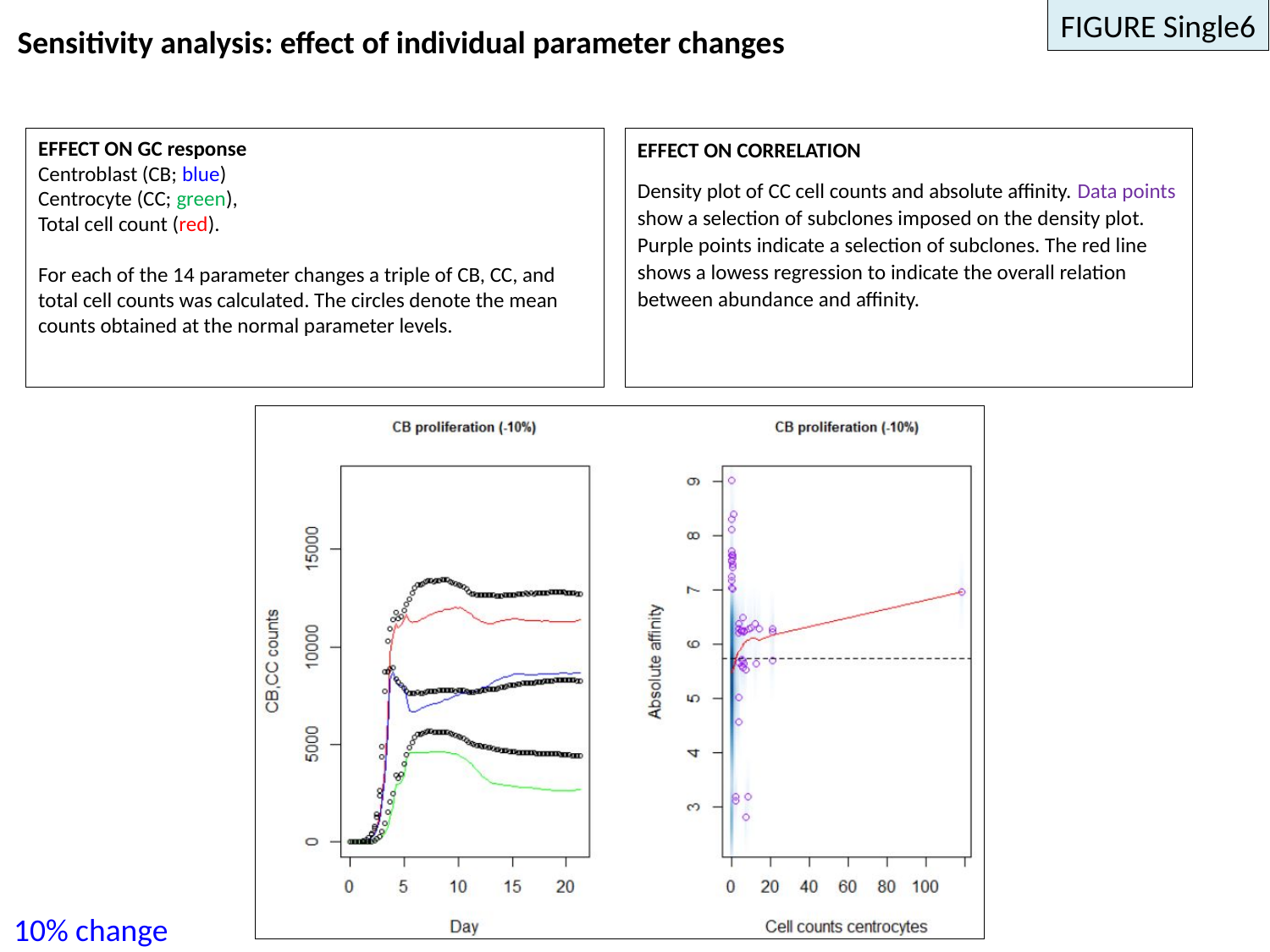

FIGURE Single6
Sensitivity analysis: effect of individual parameter changes
EFFECT ON CORRELATION
Density plot of CC cell counts and absolute affinity. Data points show a selection of subclones imposed on the density plot. Purple points indicate a selection of subclones. The red line shows a lowess regression to indicate the overall relation between abundance and affinity.
EFFECT ON GC response
Centroblast (CB; blue)
Centrocyte (CC; green),
Total cell count (red).
For each of the 14 parameter changes a triple of CB, CC, and total cell counts was calculated. The circles denote the mean counts obtained at the normal parameter levels.
10% change

## Slide 9
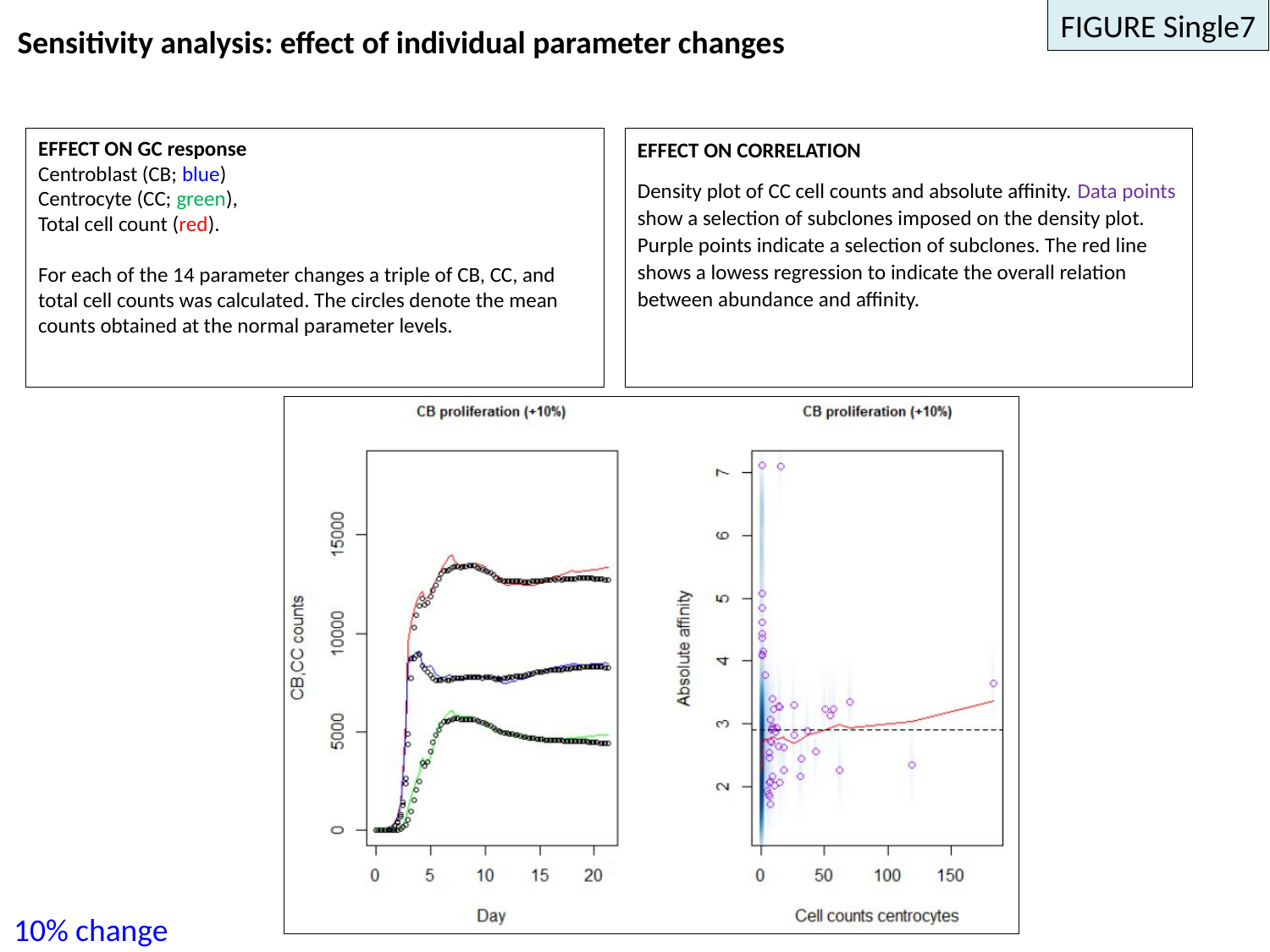

FIGURE Single7
Sensitivity analysis: effect of individual parameter changes
EFFECT ON CORRELATION
Density plot of CC cell counts and absolute affinity. Data points show a selection of subclones imposed on the density plot. Purple points indicate a selection of subclones. The red line shows a lowess regression to indicate the overall relation between abundance and affinity.
EFFECT ON GC response
Centroblast (CB; blue)
Centrocyte (CC; green),
Total cell count (red).
For each of the 14 parameter changes a triple of CB, CC, and total cell counts was calculated. The circles denote the mean counts obtained at the normal parameter levels.
10% change

## Slide 10
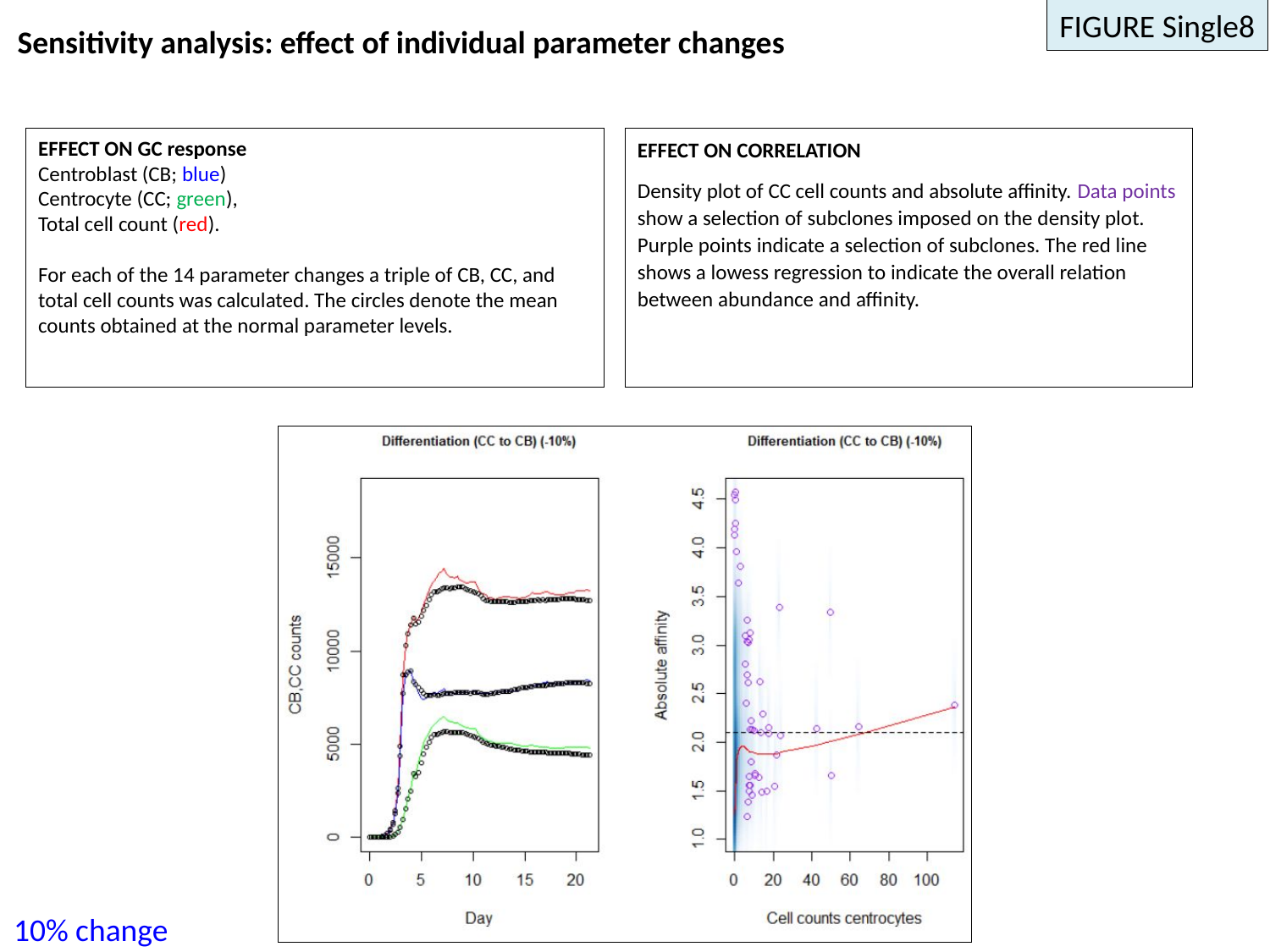

FIGURE Single8
Sensitivity analysis: effect of individual parameter changes
EFFECT ON CORRELATION
Density plot of CC cell counts and absolute affinity. Data points show a selection of subclones imposed on the density plot. Purple points indicate a selection of subclones. The red line shows a lowess regression to indicate the overall relation between abundance and affinity.
EFFECT ON GC response
Centroblast (CB; blue)
Centrocyte (CC; green),
Total cell count (red).
For each of the 14 parameter changes a triple of CB, CC, and total cell counts was calculated. The circles denote the mean counts obtained at the normal parameter levels.
10% change

## Slide 11
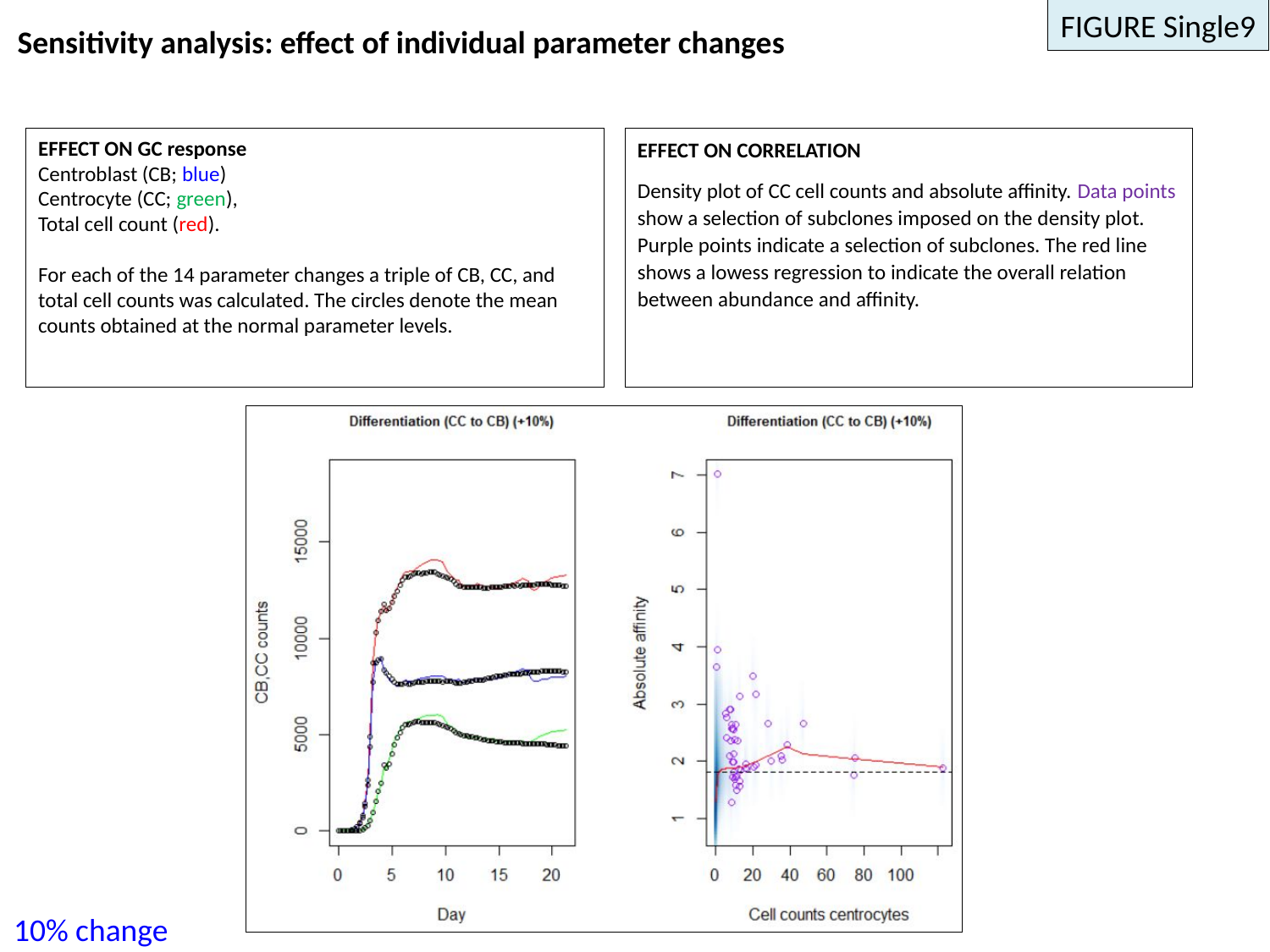

FIGURE Single9
Sensitivity analysis: effect of individual parameter changes
EFFECT ON CORRELATION
Density plot of CC cell counts and absolute affinity. Data points show a selection of subclones imposed on the density plot. Purple points indicate a selection of subclones. The red line shows a lowess regression to indicate the overall relation between abundance and affinity.
EFFECT ON GC response
Centroblast (CB; blue)
Centrocyte (CC; green),
Total cell count (red).
For each of the 14 parameter changes a triple of CB, CC, and total cell counts was calculated. The circles denote the mean counts obtained at the normal parameter levels.
10% change

## Slide 12
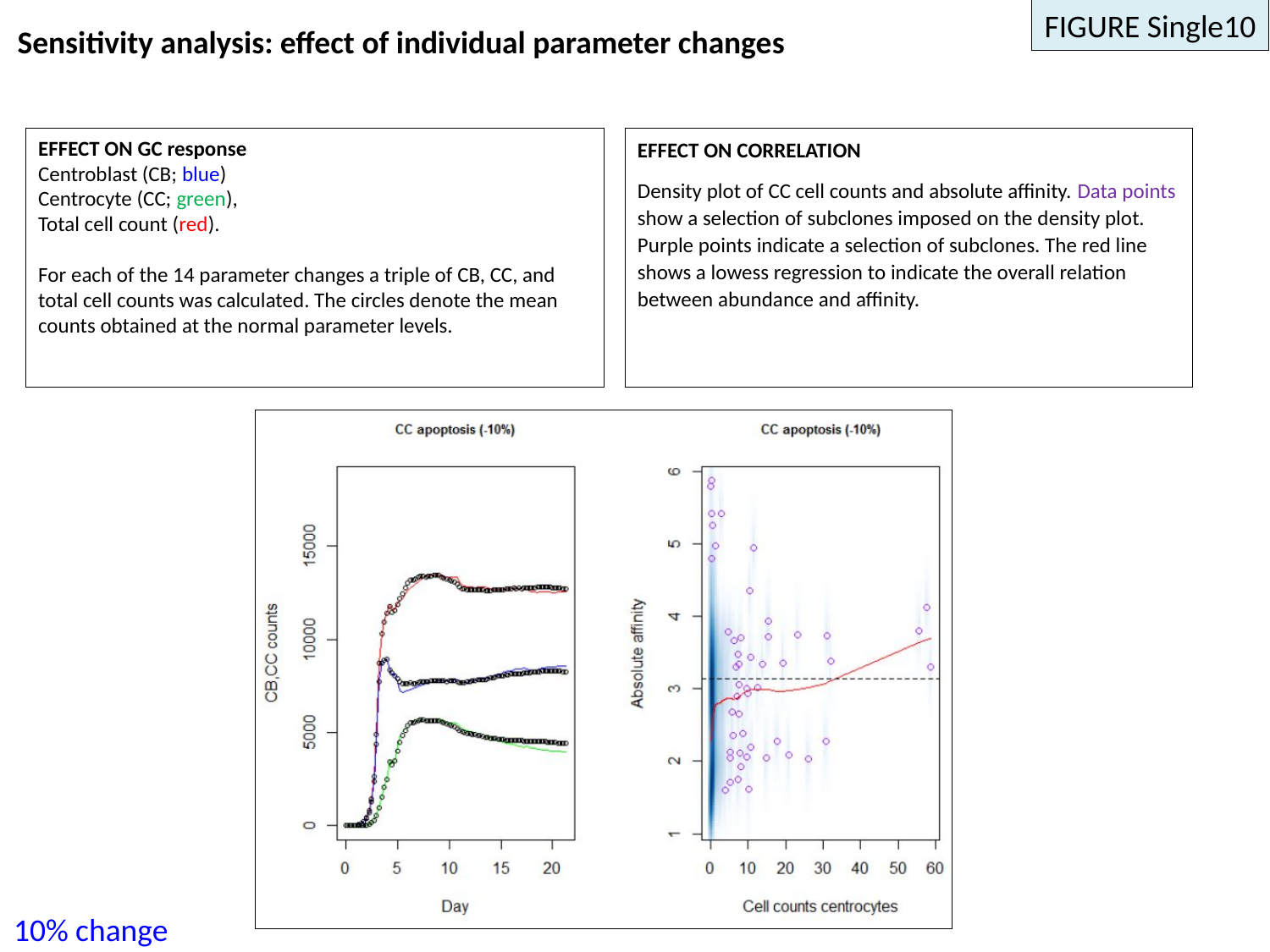

FIGURE Single10
Sensitivity analysis: effect of individual parameter changes
EFFECT ON CORRELATION
Density plot of CC cell counts and absolute affinity. Data points show a selection of subclones imposed on the density plot. Purple points indicate a selection of subclones. The red line shows a lowess regression to indicate the overall relation between abundance and affinity.
EFFECT ON GC response
Centroblast (CB; blue)
Centrocyte (CC; green),
Total cell count (red).
For each of the 14 parameter changes a triple of CB, CC, and total cell counts was calculated. The circles denote the mean counts obtained at the normal parameter levels.
10% change

## Slide 13
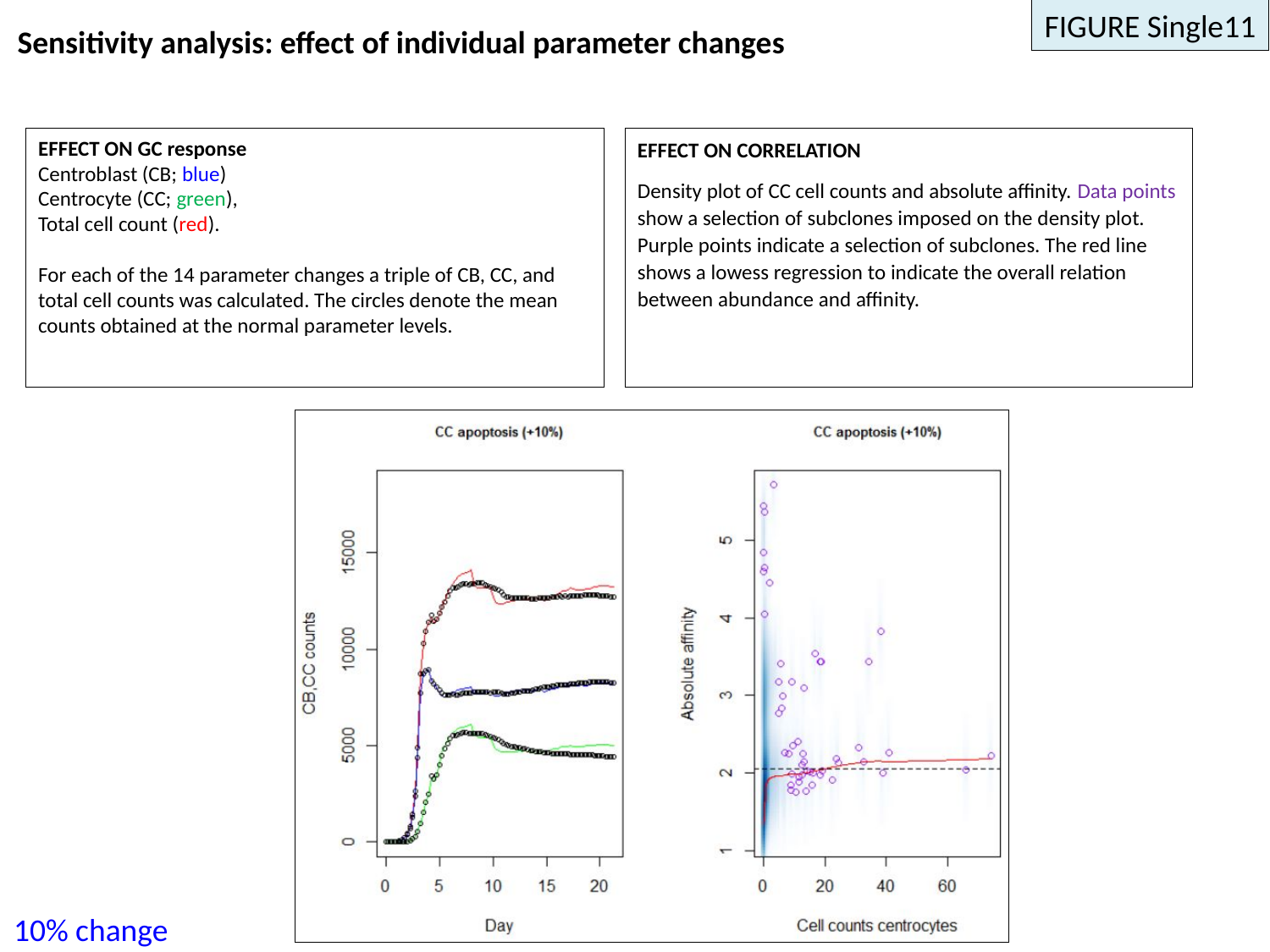

FIGURE Single11
Sensitivity analysis: effect of individual parameter changes
EFFECT ON CORRELATION
Density plot of CC cell counts and absolute affinity. Data points show a selection of subclones imposed on the density plot. Purple points indicate a selection of subclones. The red line shows a lowess regression to indicate the overall relation between abundance and affinity.
EFFECT ON GC response
Centroblast (CB; blue)
Centrocyte (CC; green),
Total cell count (red).
For each of the 14 parameter changes a triple of CB, CC, and total cell counts was calculated. The circles denote the mean counts obtained at the normal parameter levels.
10% change

## Slide 14
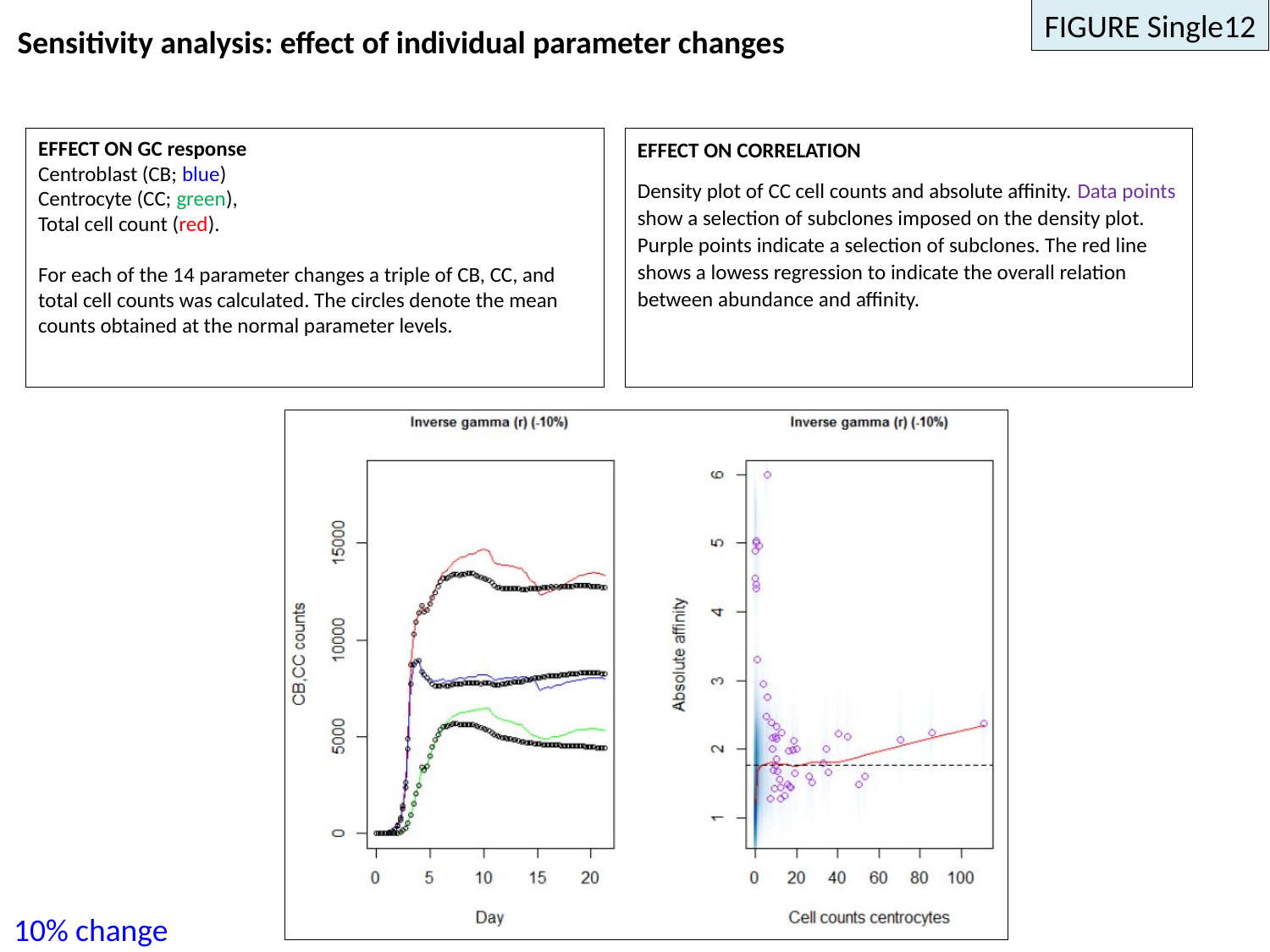

FIGURE Single12
Sensitivity analysis: effect of individual parameter changes
EFFECT ON CORRELATION
Density plot of CC cell counts and absolute affinity. Data points show a selection of subclones imposed on the density plot. Purple points indicate a selection of subclones. The red line shows a lowess regression to indicate the overall relation between abundance and affinity.
EFFECT ON GC response
Centroblast (CB; blue)
Centrocyte (CC; green),
Total cell count (red).
For each of the 14 parameter changes a triple of CB, CC, and total cell counts was calculated. The circles denote the mean counts obtained at the normal parameter levels.
10% change

## Slide 15
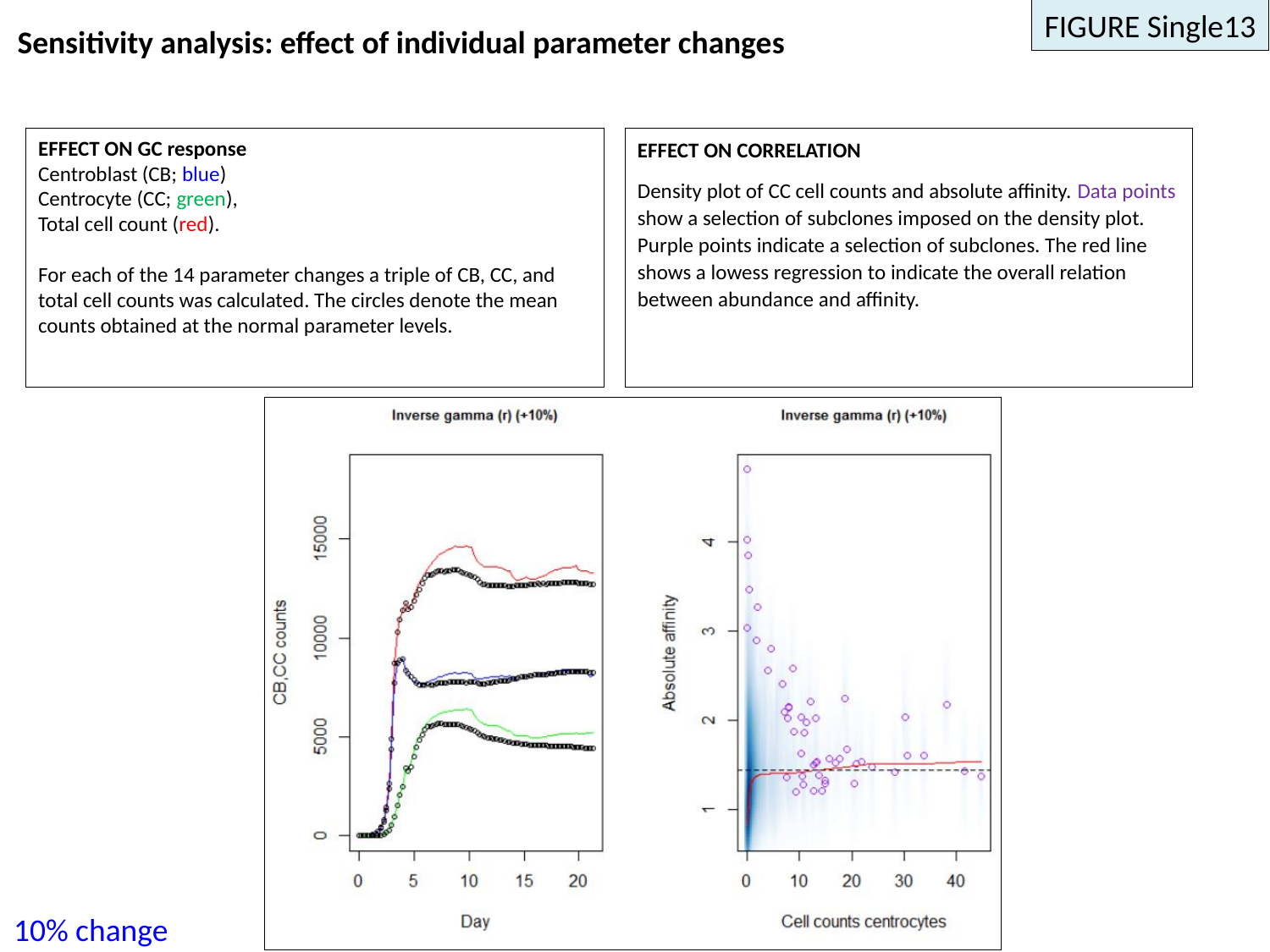

FIGURE Single13
Sensitivity analysis: effect of individual parameter changes
EFFECT ON CORRELATION
Density plot of CC cell counts and absolute affinity. Data points show a selection of subclones imposed on the density plot. Purple points indicate a selection of subclones. The red line shows a lowess regression to indicate the overall relation between abundance and affinity.
EFFECT ON GC response
Centroblast (CB; blue)
Centrocyte (CC; green),
Total cell count (red).
For each of the 14 parameter changes a triple of CB, CC, and total cell counts was calculated. The circles denote the mean counts obtained at the normal parameter levels.
10% change

## Slide 16
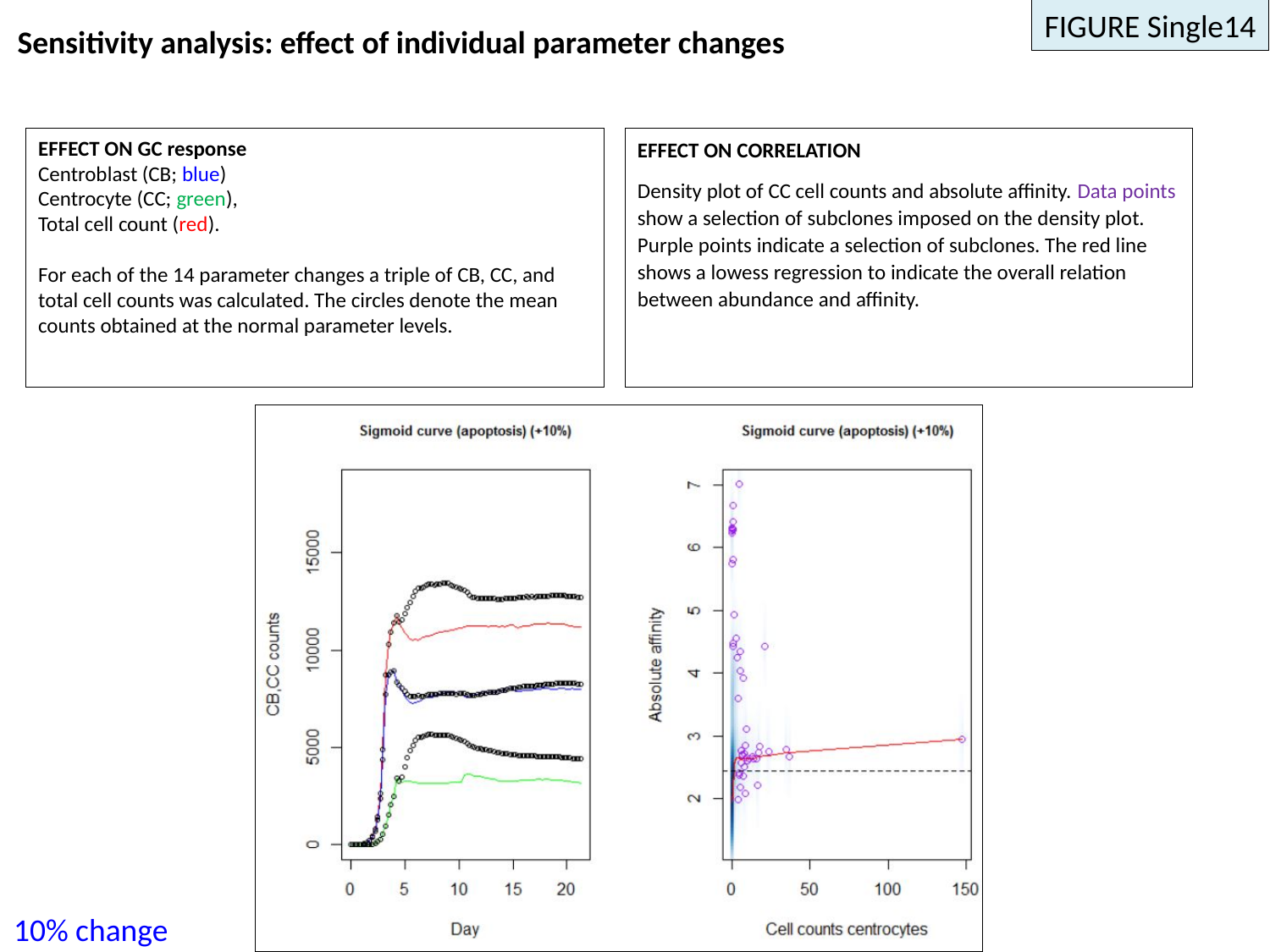

FIGURE Single14
Sensitivity analysis: effect of individual parameter changes
EFFECT ON CORRELATION
Density plot of CC cell counts and absolute affinity. Data points show a selection of subclones imposed on the density plot. Purple points indicate a selection of subclones. The red line shows a lowess regression to indicate the overall relation between abundance and affinity.
EFFECT ON GC response
Centroblast (CB; blue)
Centrocyte (CC; green),
Total cell count (red).
For each of the 14 parameter changes a triple of CB, CC, and total cell counts was calculated. The circles denote the mean counts obtained at the normal parameter levels.
10% change

## Slide 17
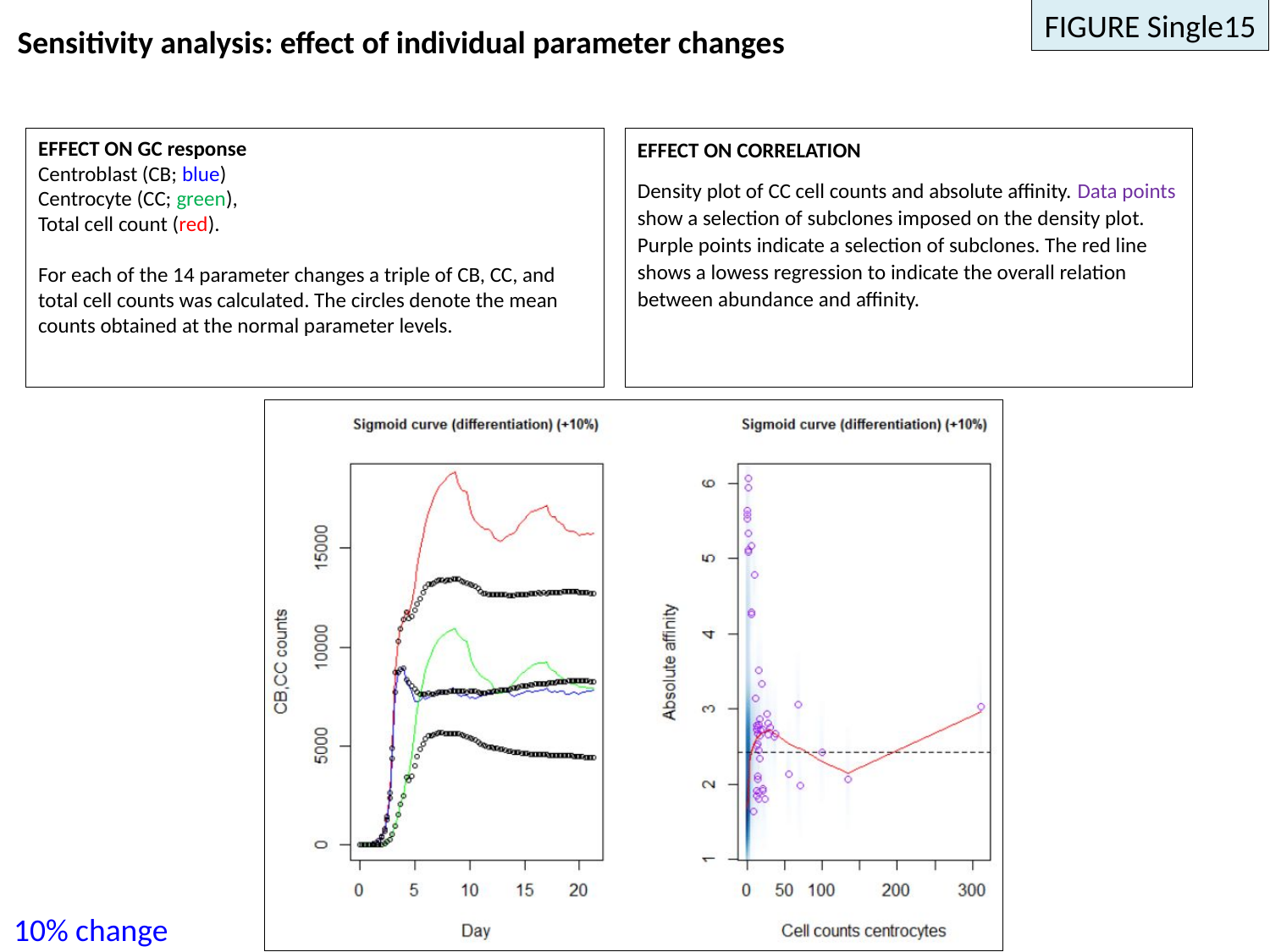

FIGURE Single15
Sensitivity analysis: effect of individual parameter changes
EFFECT ON CORRELATION
Density plot of CC cell counts and absolute affinity. Data points show a selection of subclones imposed on the density plot. Purple points indicate a selection of subclones. The red line shows a lowess regression to indicate the overall relation between abundance and affinity.
EFFECT ON GC response
Centroblast (CB; blue)
Centrocyte (CC; green),
Total cell count (red).
For each of the 14 parameter changes a triple of CB, CC, and total cell counts was calculated. The circles denote the mean counts obtained at the normal parameter levels.
10% change

## Slide 18
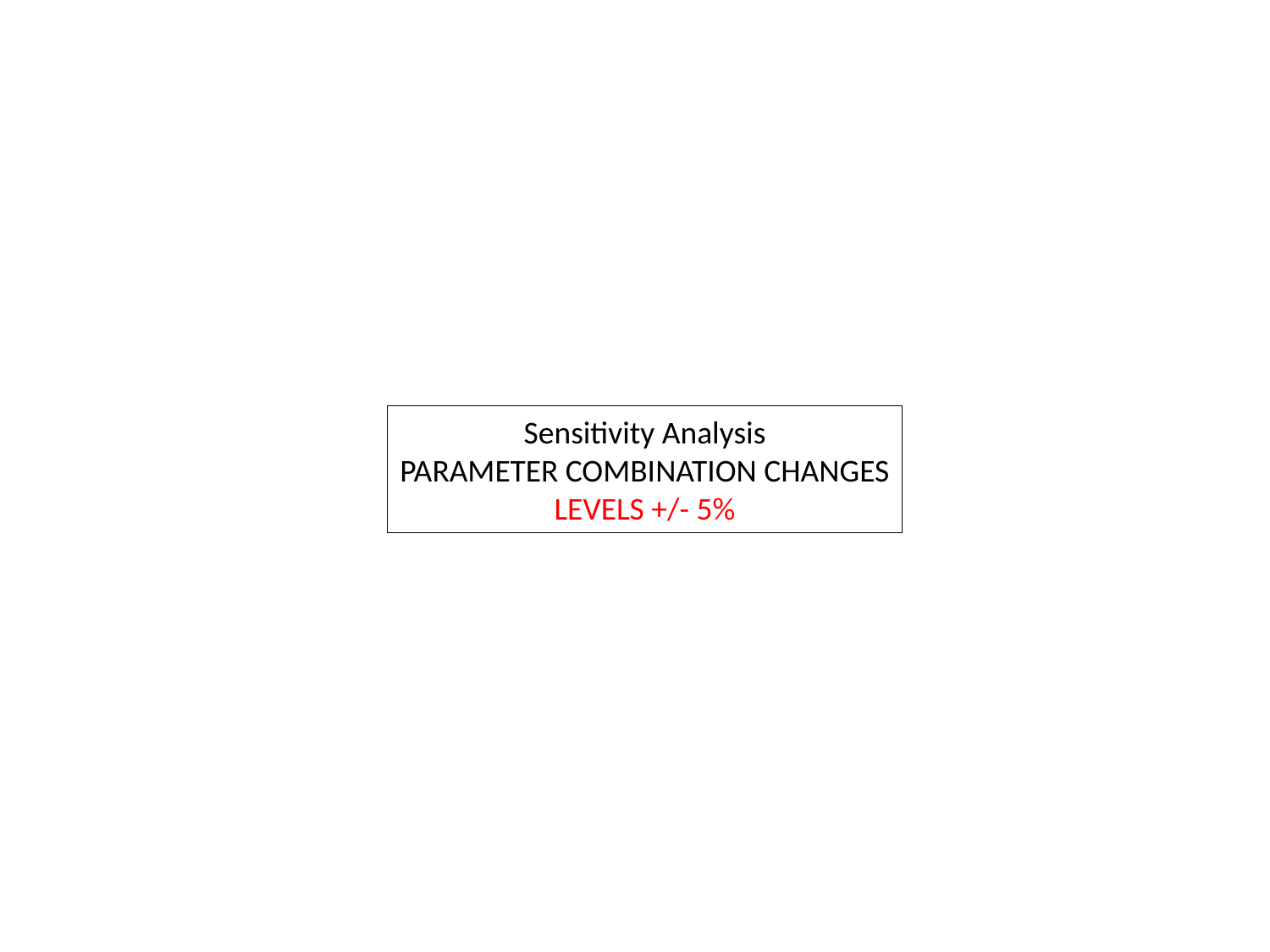

Sensitivity Analysis
PARAMETER COMBINATION CHANGES
LEVELS +/- 5%

## Slide 19
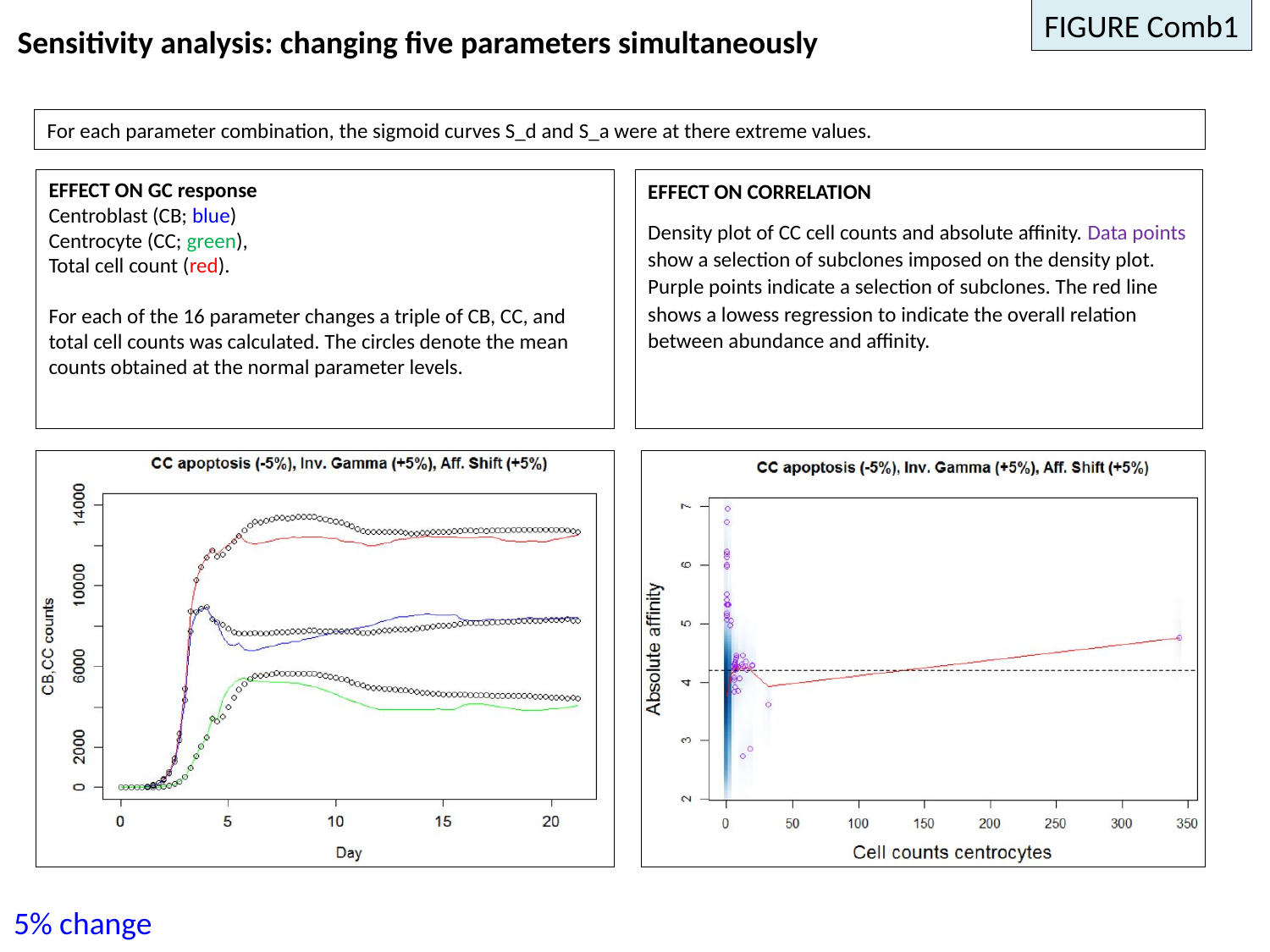

FIGURE Comb1
Sensitivity analysis: changing five parameters simultaneously
For each parameter combination, the sigmoid curves S_d and S_a were at there extreme values.
EFFECT ON CORRELATION
Density plot of CC cell counts and absolute affinity. Data points show a selection of subclones imposed on the density plot. Purple points indicate a selection of subclones. The red line shows a lowess regression to indicate the overall relation between abundance and affinity.
EFFECT ON GC response
Centroblast (CB; blue)
Centrocyte (CC; green),
Total cell count (red).
For each of the 16 parameter changes a triple of CB, CC, and total cell counts was calculated. The circles denote the mean counts obtained at the normal parameter levels.
5% change

## Slide 20
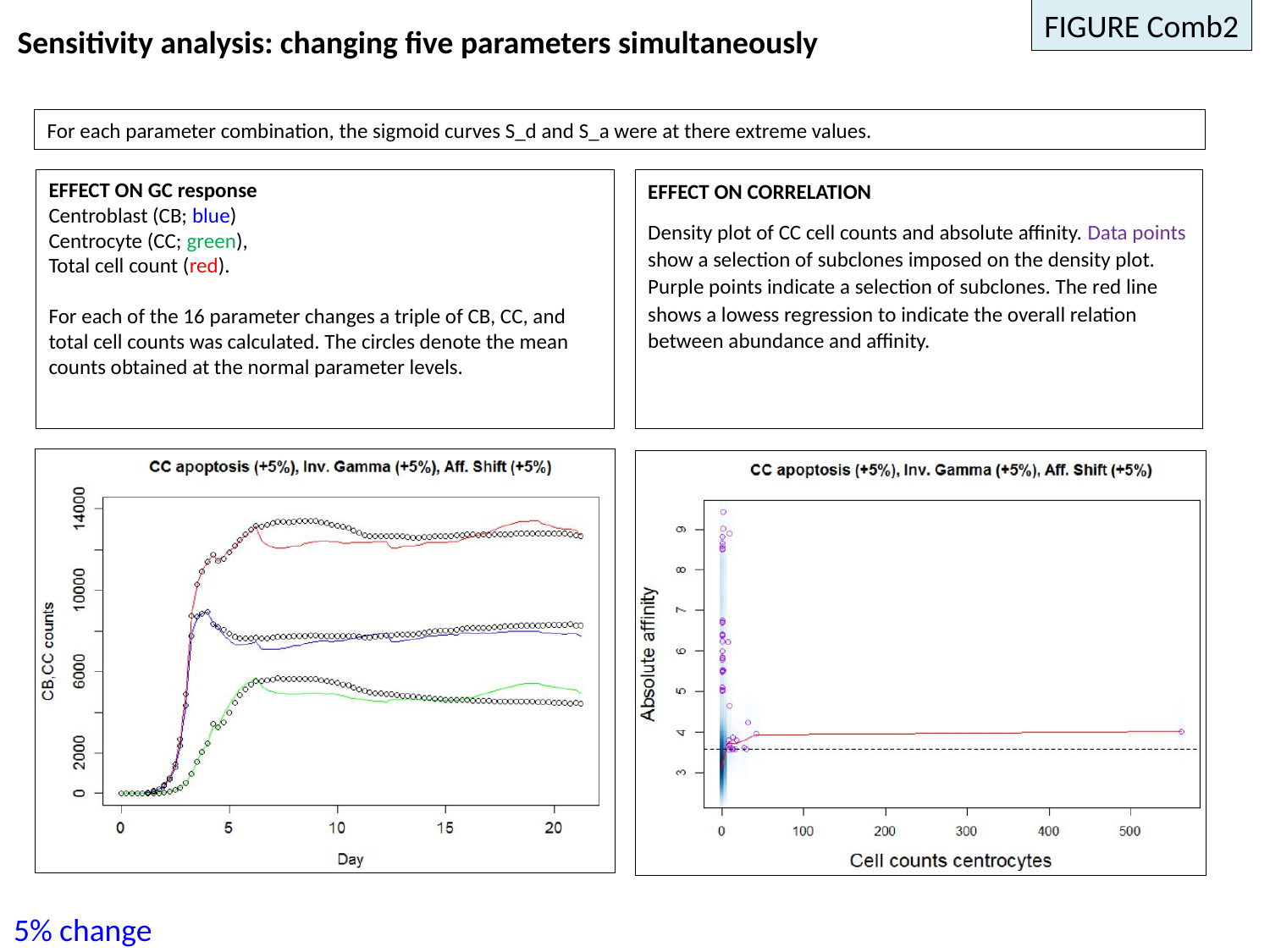

FIGURE Comb2
Sensitivity analysis: changing five parameters simultaneously
For each parameter combination, the sigmoid curves S_d and S_a were at there extreme values.
EFFECT ON CORRELATION
Density plot of CC cell counts and absolute affinity. Data points show a selection of subclones imposed on the density plot. Purple points indicate a selection of subclones. The red line shows a lowess regression to indicate the overall relation between abundance and affinity.
EFFECT ON GC response
Centroblast (CB; blue)
Centrocyte (CC; green),
Total cell count (red).
For each of the 16 parameter changes a triple of CB, CC, and total cell counts was calculated. The circles denote the mean counts obtained at the normal parameter levels.
5% change

## Slide 21
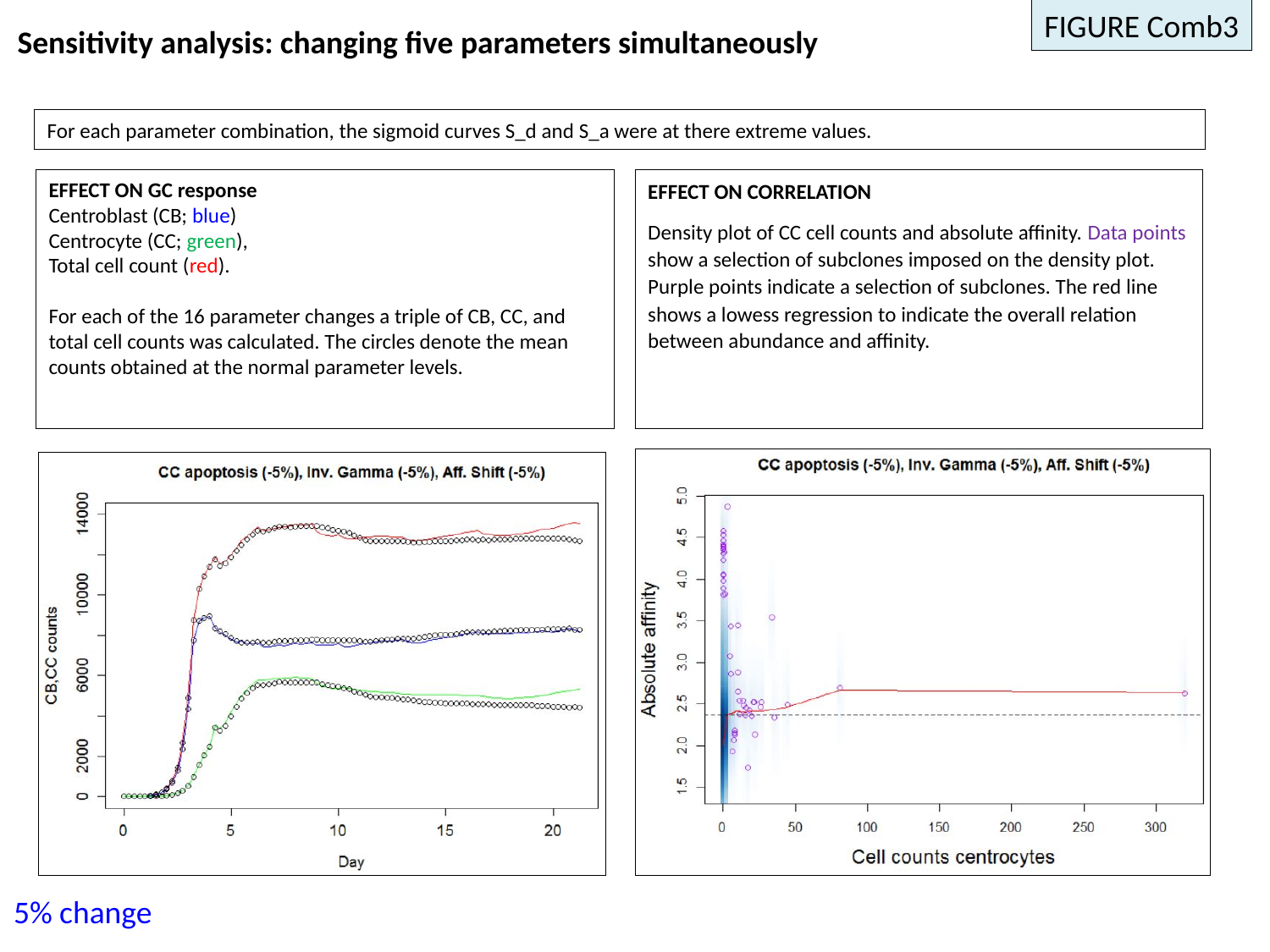

FIGURE Comb3
Sensitivity analysis: changing five parameters simultaneously
For each parameter combination, the sigmoid curves S_d and S_a were at there extreme values.
EFFECT ON CORRELATION
Density plot of CC cell counts and absolute affinity. Data points show a selection of subclones imposed on the density plot. Purple points indicate a selection of subclones. The red line shows a lowess regression to indicate the overall relation between abundance and affinity.
EFFECT ON GC response
Centroblast (CB; blue)
Centrocyte (CC; green),
Total cell count (red).
For each of the 16 parameter changes a triple of CB, CC, and total cell counts was calculated. The circles denote the mean counts obtained at the normal parameter levels.
5% change

## Slide 22
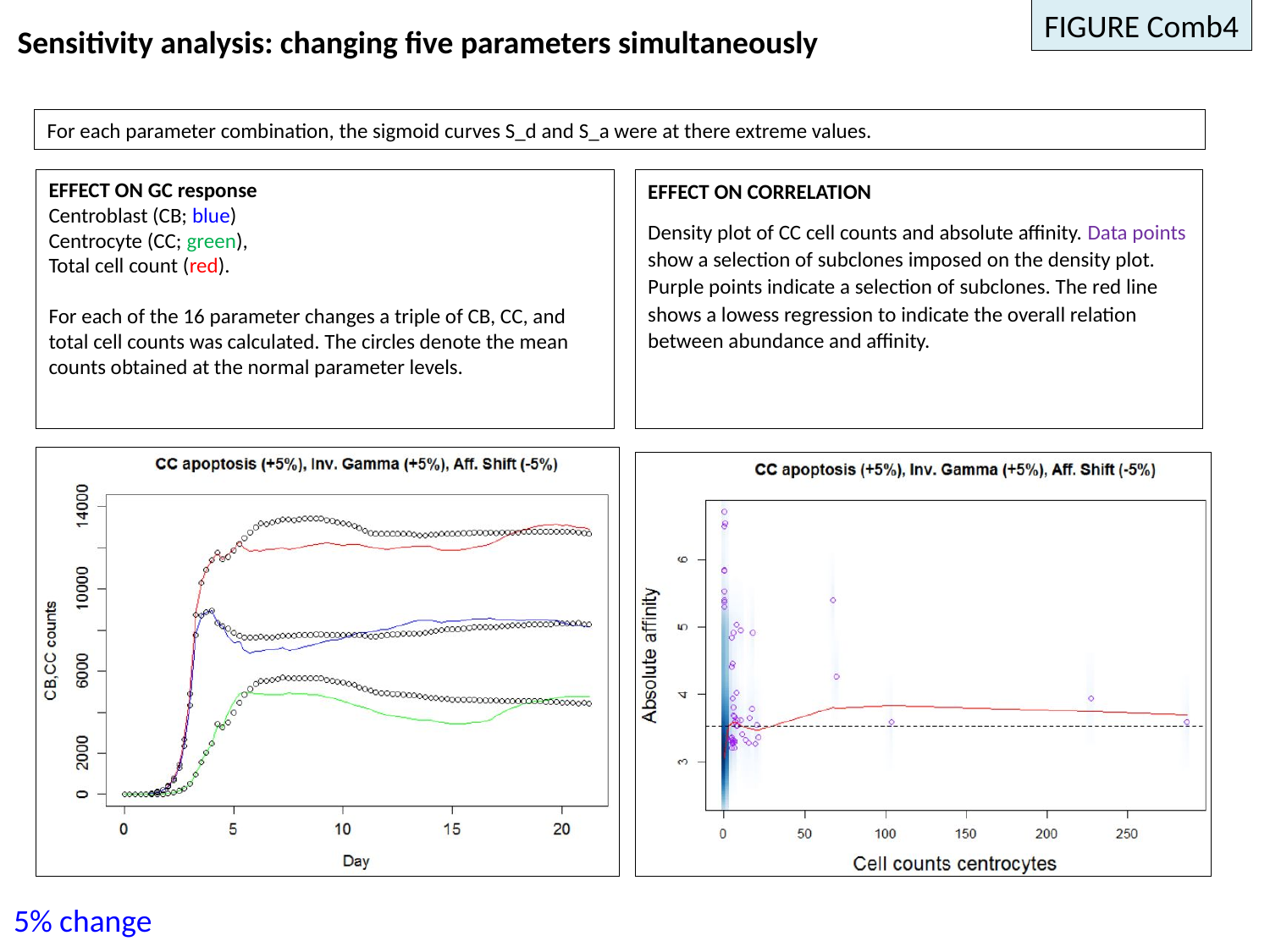

FIGURE Comb4
Sensitivity analysis: changing five parameters simultaneously
For each parameter combination, the sigmoid curves S_d and S_a were at there extreme values.
EFFECT ON CORRELATION
Density plot of CC cell counts and absolute affinity. Data points show a selection of subclones imposed on the density plot. Purple points indicate a selection of subclones. The red line shows a lowess regression to indicate the overall relation between abundance and affinity.
EFFECT ON GC response
Centroblast (CB; blue)
Centrocyte (CC; green),
Total cell count (red).
For each of the 16 parameter changes a triple of CB, CC, and total cell counts was calculated. The circles denote the mean counts obtained at the normal parameter levels.
5% change

## Slide 23
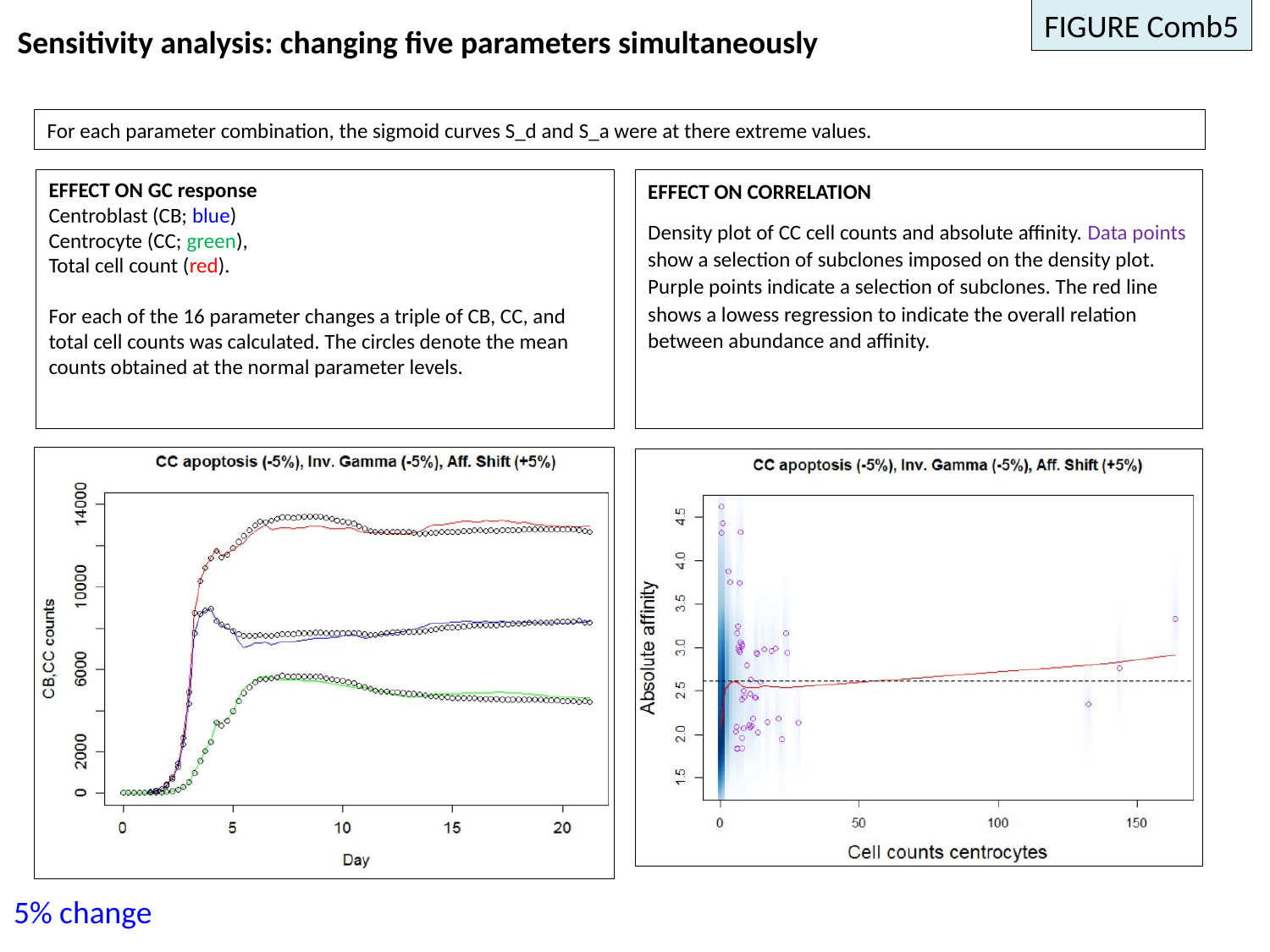

FIGURE Comb5
Sensitivity analysis: changing five parameters simultaneously
For each parameter combination, the sigmoid curves S_d and S_a were at there extreme values.
EFFECT ON CORRELATION
Density plot of CC cell counts and absolute affinity. Data points show a selection of subclones imposed on the density plot. Purple points indicate a selection of subclones. The red line shows a lowess regression to indicate the overall relation between abundance and affinity.
EFFECT ON GC response
Centroblast (CB; blue)
Centrocyte (CC; green),
Total cell count (red).
For each of the 16 parameter changes a triple of CB, CC, and total cell counts was calculated. The circles denote the mean counts obtained at the normal parameter levels.
5% change

## Slide 24
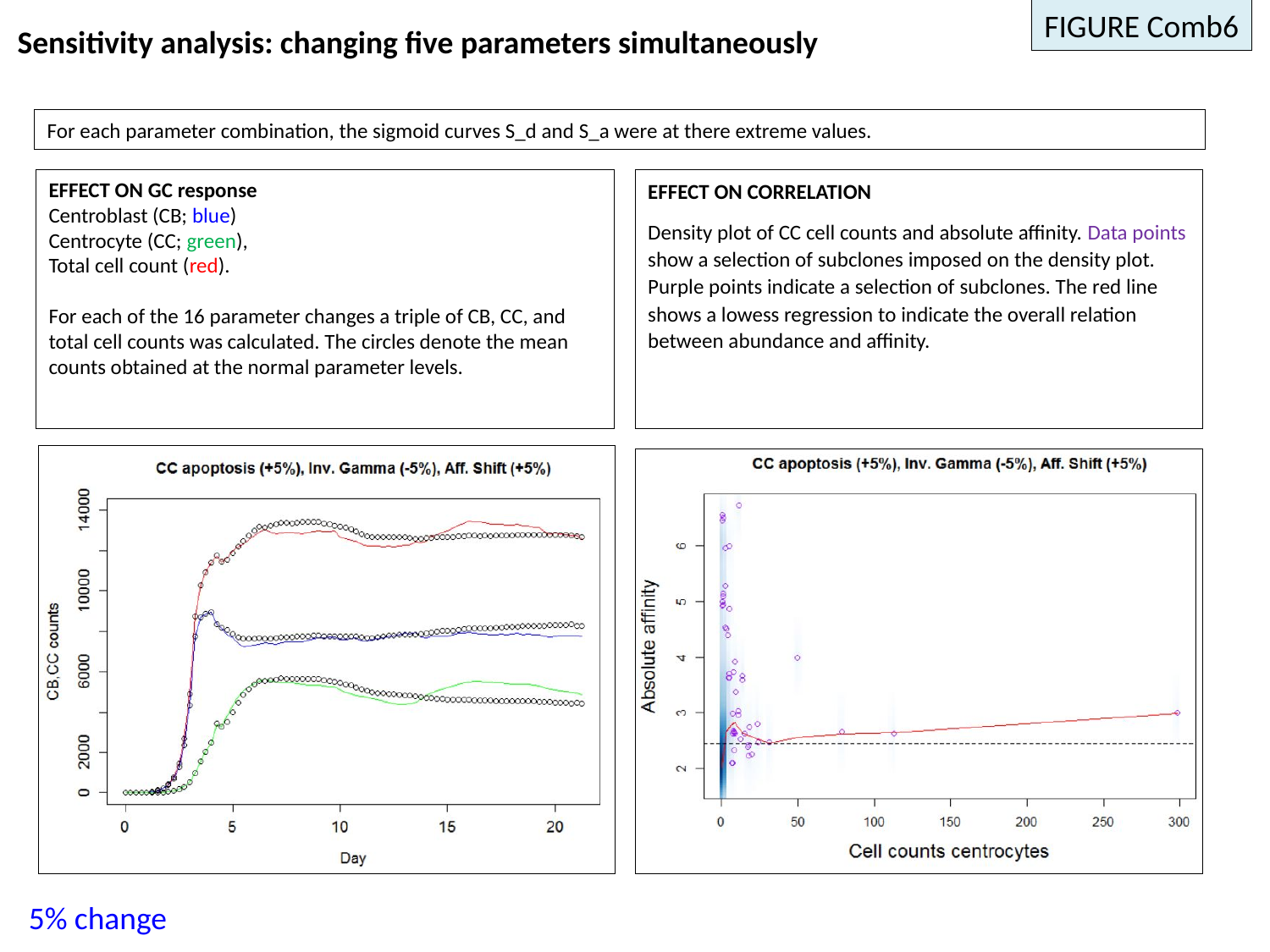

FIGURE Comb6
Sensitivity analysis: changing five parameters simultaneously
For each parameter combination, the sigmoid curves S_d and S_a were at there extreme values.
EFFECT ON CORRELATION
Density plot of CC cell counts and absolute affinity. Data points show a selection of subclones imposed on the density plot. Purple points indicate a selection of subclones. The red line shows a lowess regression to indicate the overall relation between abundance and affinity.
EFFECT ON GC response
Centroblast (CB; blue)
Centrocyte (CC; green),
Total cell count (red).
For each of the 16 parameter changes a triple of CB, CC, and total cell counts was calculated. The circles denote the mean counts obtained at the normal parameter levels.
5% change

## Slide 25
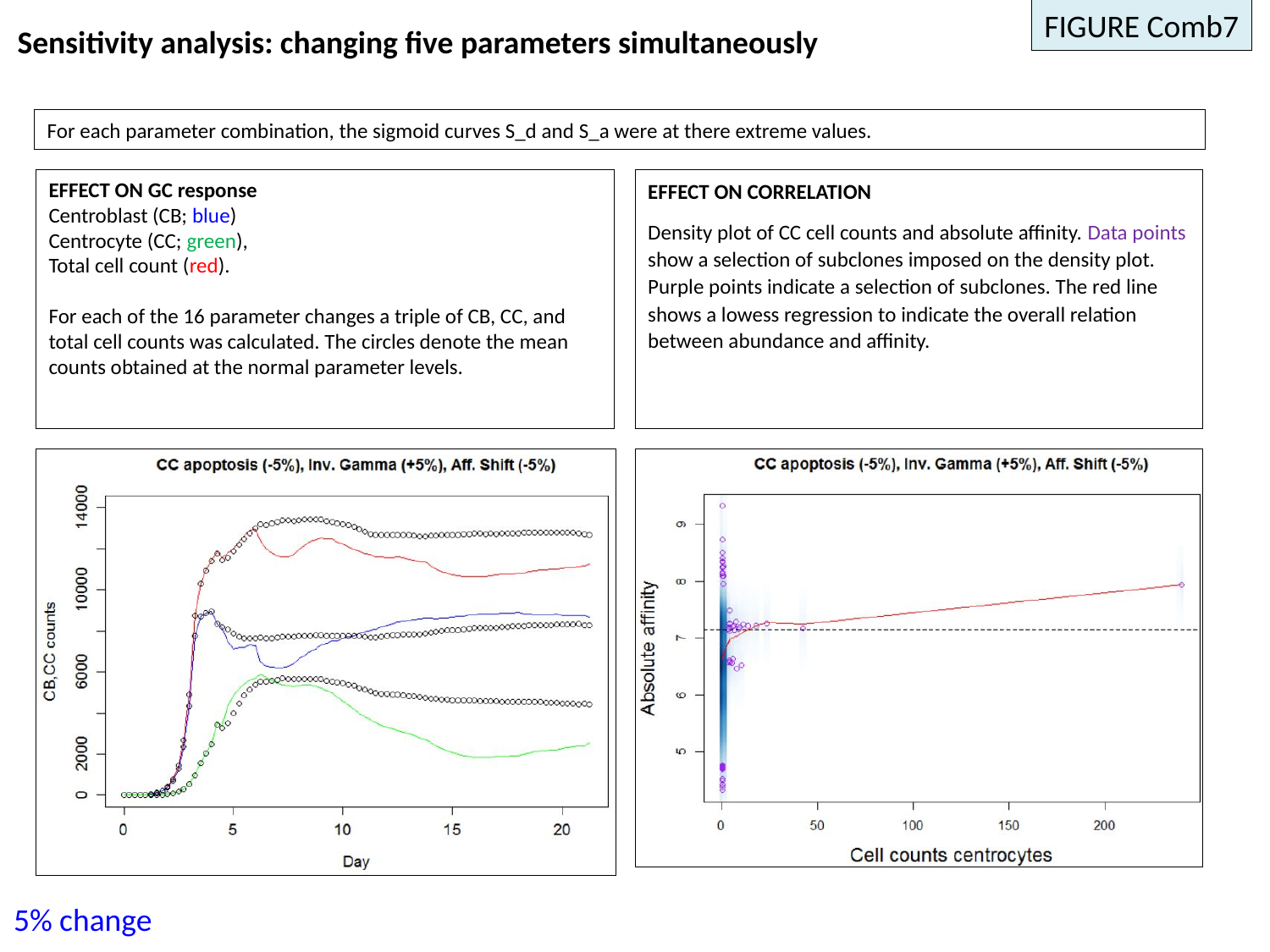

FIGURE Comb7
Sensitivity analysis: changing five parameters simultaneously
For each parameter combination, the sigmoid curves S_d and S_a were at there extreme values.
EFFECT ON CORRELATION
Density plot of CC cell counts and absolute affinity. Data points show a selection of subclones imposed on the density plot. Purple points indicate a selection of subclones. The red line shows a lowess regression to indicate the overall relation between abundance and affinity.
EFFECT ON GC response
Centroblast (CB; blue)
Centrocyte (CC; green),
Total cell count (red).
For each of the 16 parameter changes a triple of CB, CC, and total cell counts was calculated. The circles denote the mean counts obtained at the normal parameter levels.
5% change

## Slide 26
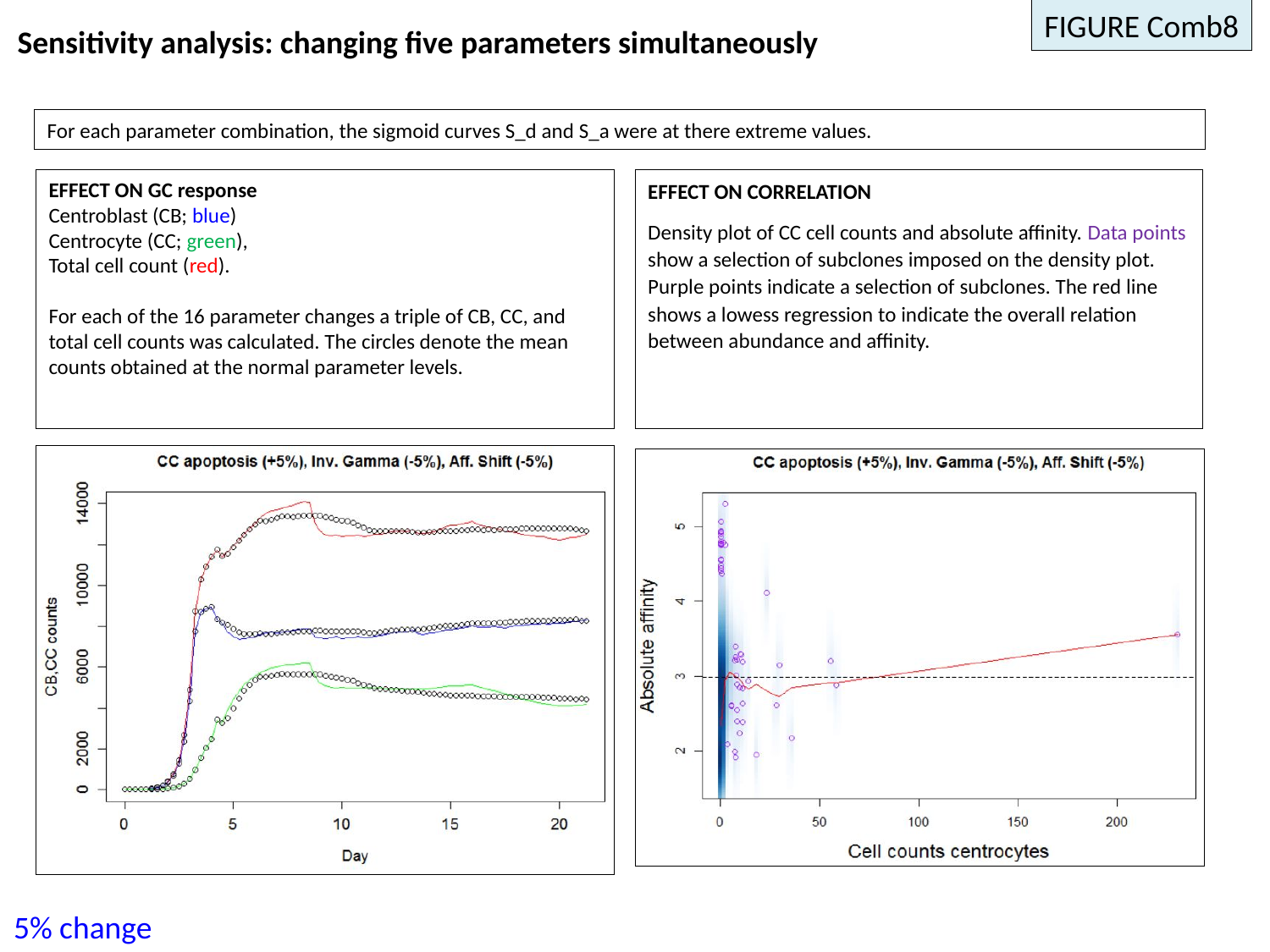

FIGURE Comb8
Sensitivity analysis: changing five parameters simultaneously
For each parameter combination, the sigmoid curves S_d and S_a were at there extreme values.
EFFECT ON CORRELATION
Density plot of CC cell counts and absolute affinity. Data points show a selection of subclones imposed on the density plot. Purple points indicate a selection of subclones. The red line shows a lowess regression to indicate the overall relation between abundance and affinity.
EFFECT ON GC response
Centroblast (CB; blue)
Centrocyte (CC; green),
Total cell count (red).
For each of the 16 parameter changes a triple of CB, CC, and total cell counts was calculated. The circles denote the mean counts obtained at the normal parameter levels.
5% change

## Slide 27
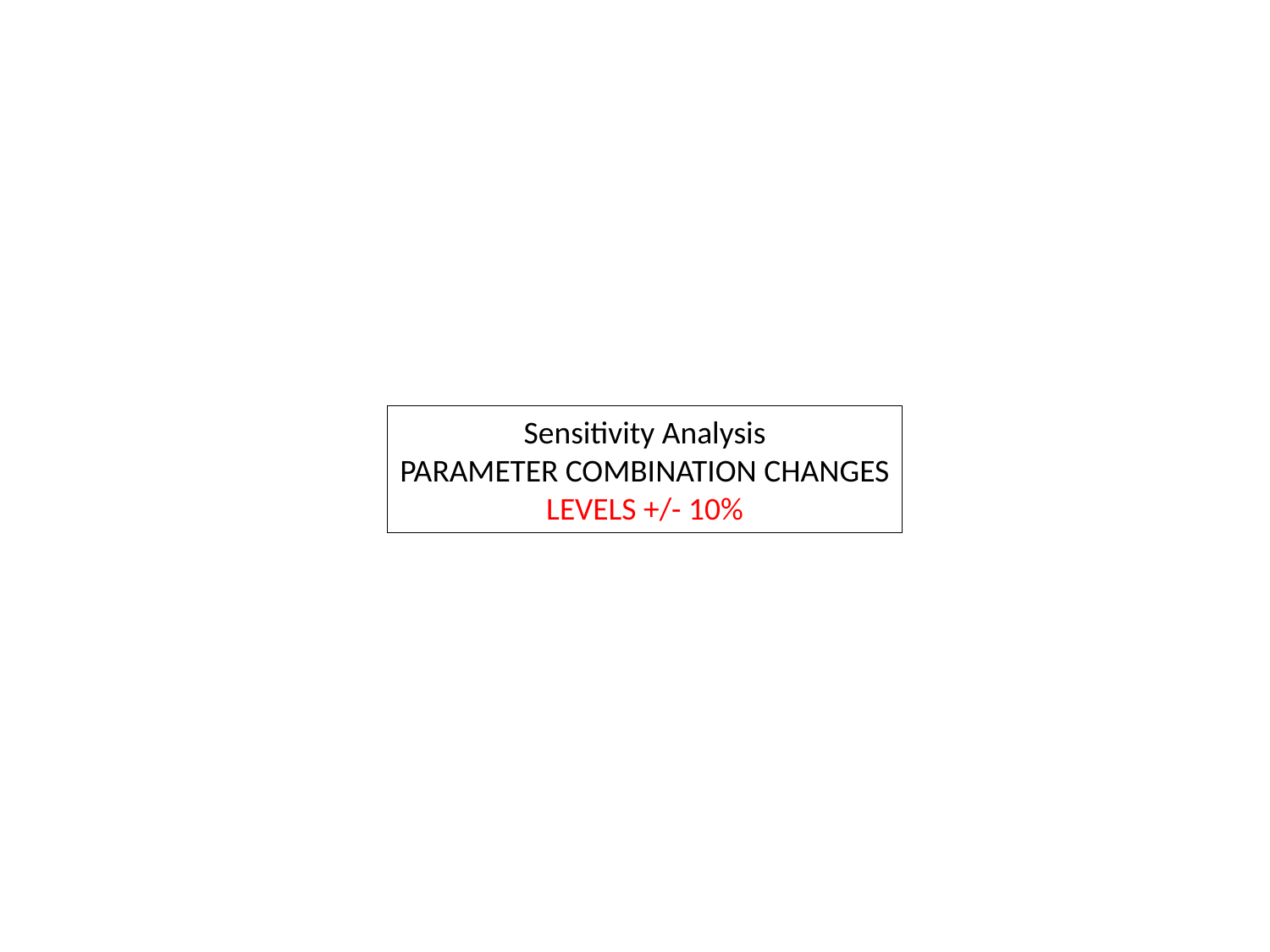

Sensitivity Analysis
PARAMETER COMBINATION CHANGES
LEVELS +/- 10%

## Slide 28
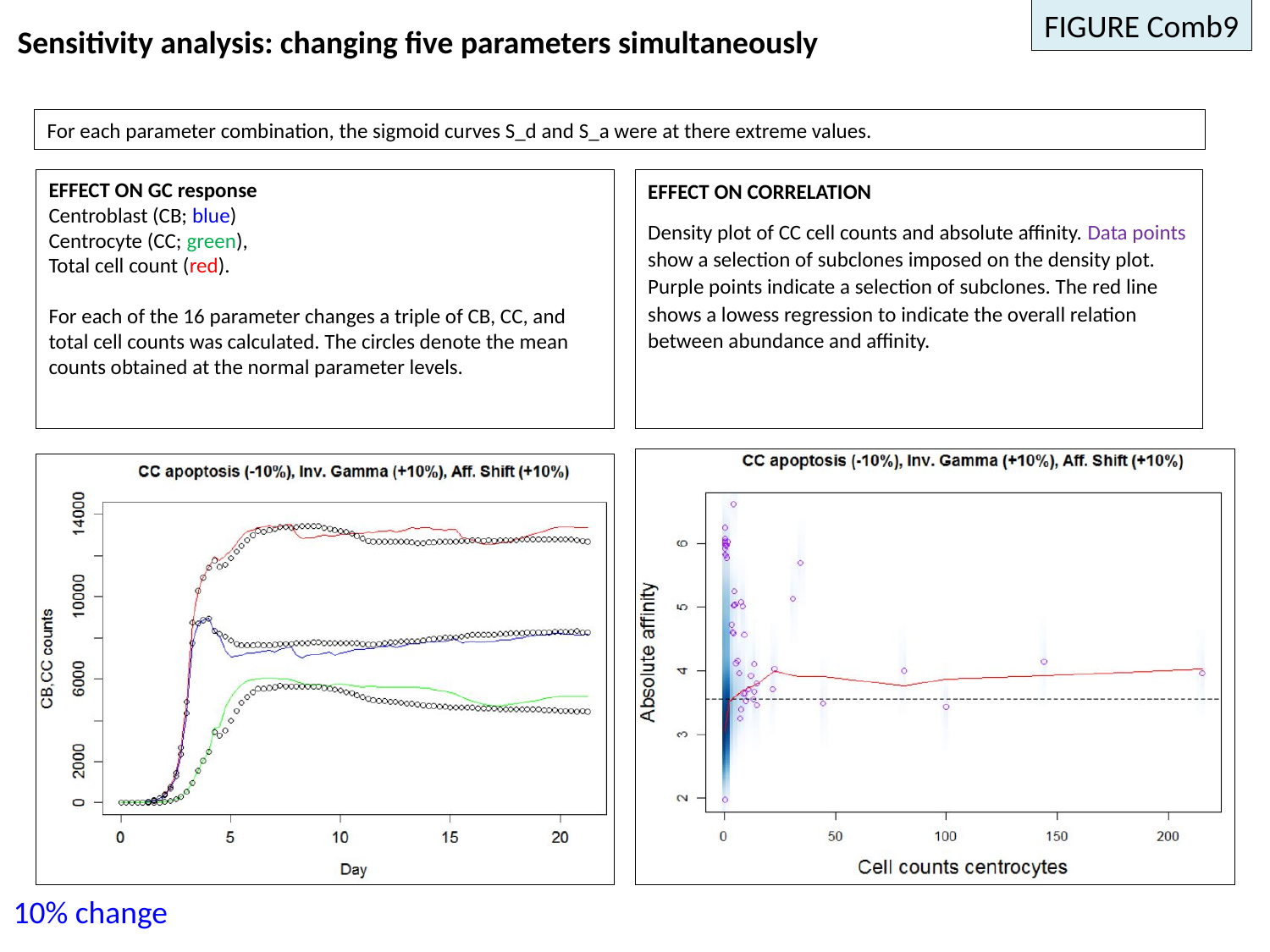

FIGURE Comb9
Sensitivity analysis: changing five parameters simultaneously
For each parameter combination, the sigmoid curves S_d and S_a were at there extreme values.
EFFECT ON CORRELATION
Density plot of CC cell counts and absolute affinity. Data points show a selection of subclones imposed on the density plot. Purple points indicate a selection of subclones. The red line shows a lowess regression to indicate the overall relation between abundance and affinity.
EFFECT ON GC response
Centroblast (CB; blue)
Centrocyte (CC; green),
Total cell count (red).
For each of the 16 parameter changes a triple of CB, CC, and total cell counts was calculated. The circles denote the mean counts obtained at the normal parameter levels.
10% change

## Slide 29
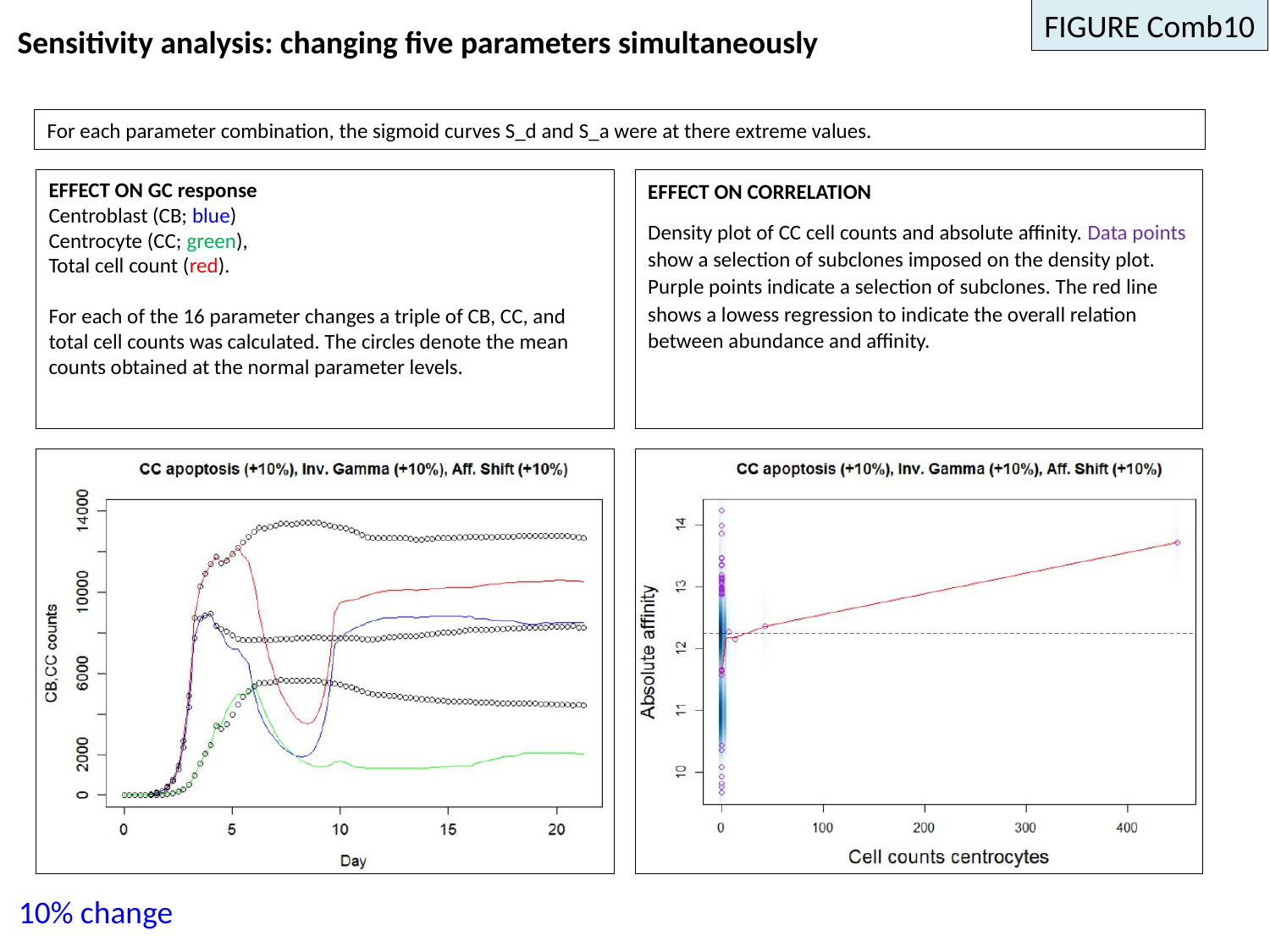

FIGURE Comb10
Sensitivity analysis: changing five parameters simultaneously
For each parameter combination, the sigmoid curves S_d and S_a were at there extreme values.
EFFECT ON CORRELATION
Density plot of CC cell counts and absolute affinity. Data points show a selection of subclones imposed on the density plot. Purple points indicate a selection of subclones. The red line shows a lowess regression to indicate the overall relation between abundance and affinity.
EFFECT ON GC response
Centroblast (CB; blue)
Centrocyte (CC; green),
Total cell count (red).
For each of the 16 parameter changes a triple of CB, CC, and total cell counts was calculated. The circles denote the mean counts obtained at the normal parameter levels.
10% change

## Slide 30
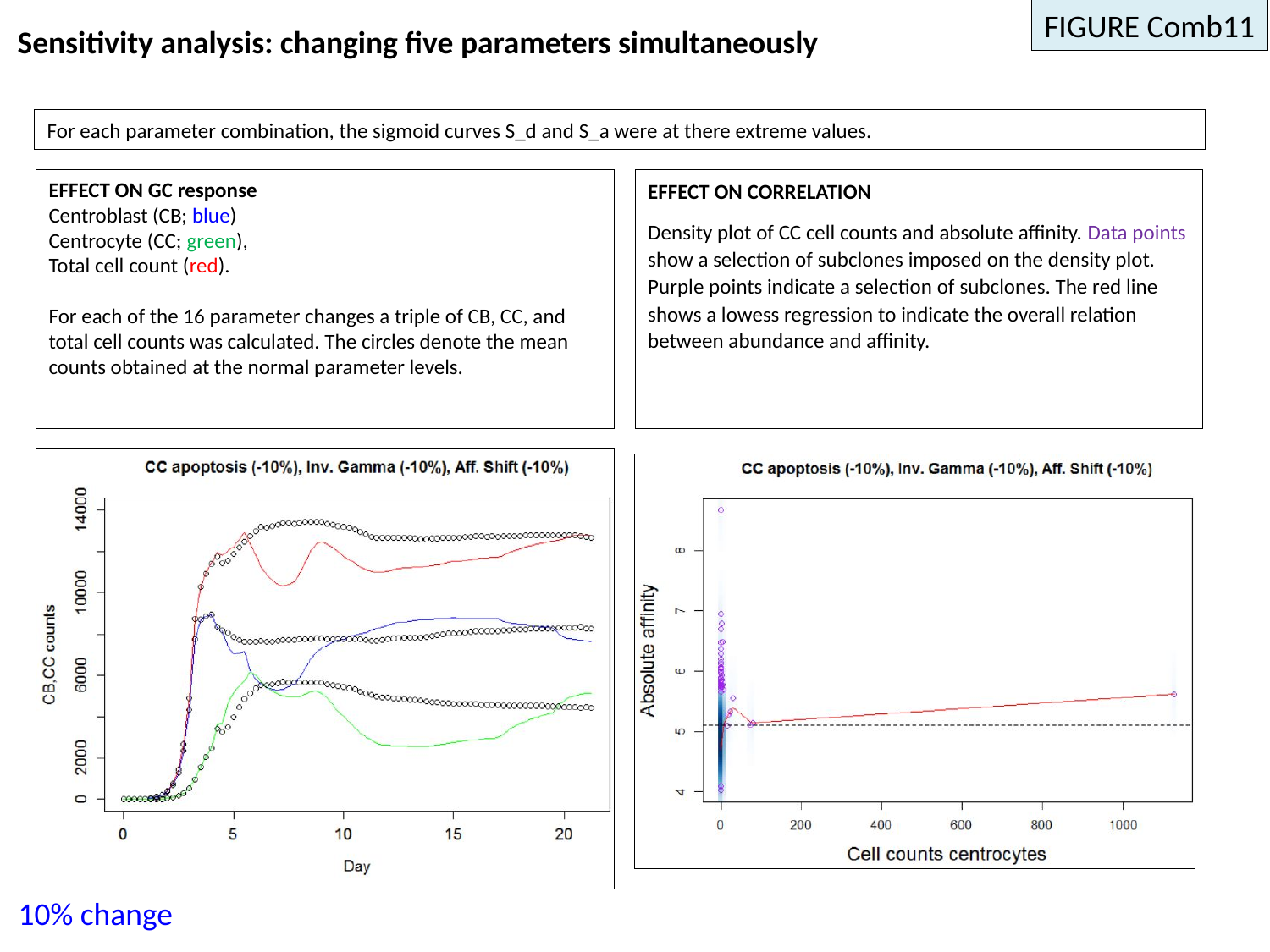

FIGURE Comb11
Sensitivity analysis: changing five parameters simultaneously
For each parameter combination, the sigmoid curves S_d and S_a were at there extreme values.
EFFECT ON CORRELATION
Density plot of CC cell counts and absolute affinity. Data points show a selection of subclones imposed on the density plot. Purple points indicate a selection of subclones. The red line shows a lowess regression to indicate the overall relation between abundance and affinity.
EFFECT ON GC response
Centroblast (CB; blue)
Centrocyte (CC; green),
Total cell count (red).
For each of the 16 parameter changes a triple of CB, CC, and total cell counts was calculated. The circles denote the mean counts obtained at the normal parameter levels.
10% change

## Slide 31
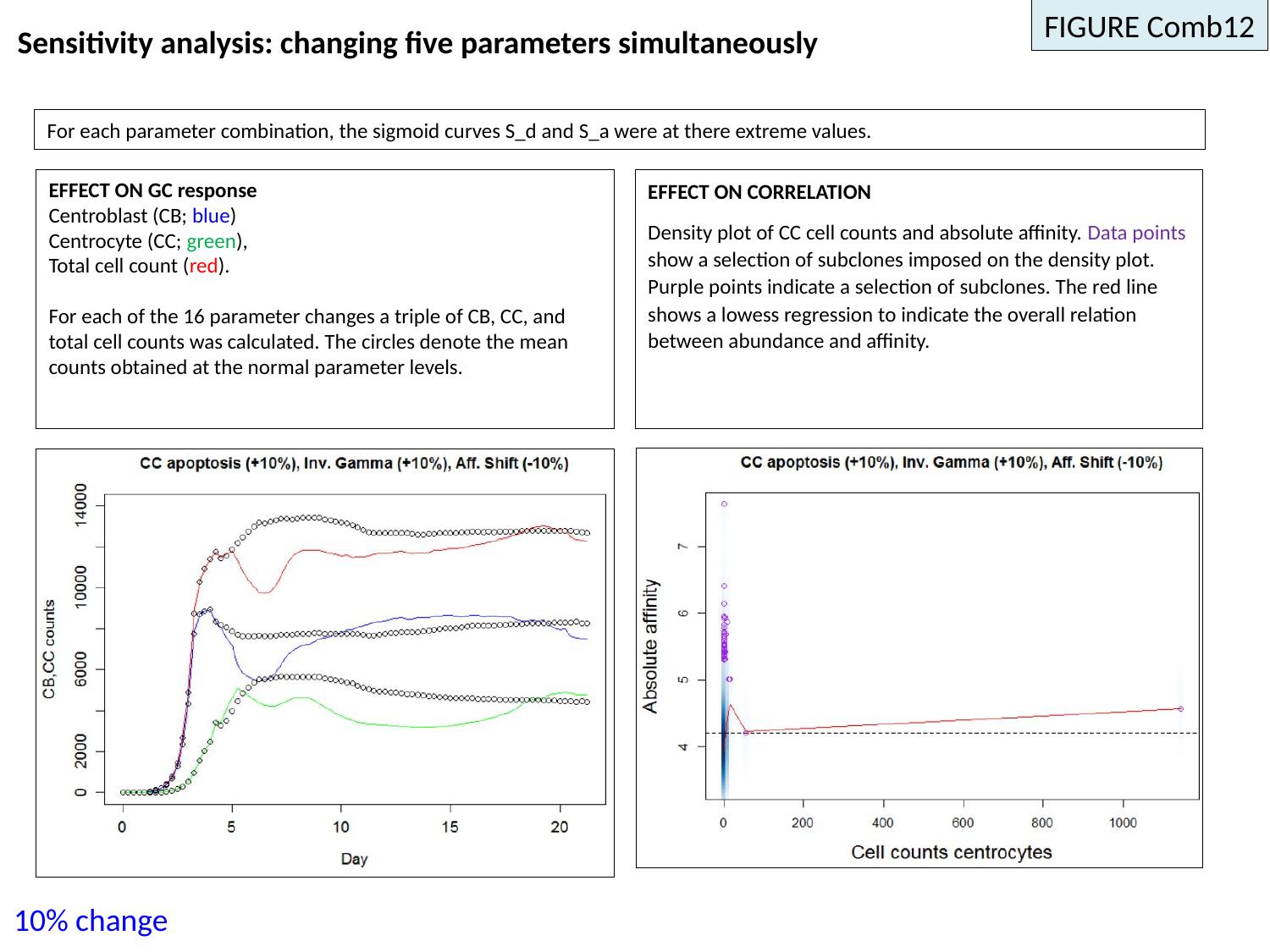

FIGURE Comb12
Sensitivity analysis: changing five parameters simultaneously
For each parameter combination, the sigmoid curves S_d and S_a were at there extreme values.
EFFECT ON CORRELATION
Density plot of CC cell counts and absolute affinity. Data points show a selection of subclones imposed on the density plot. Purple points indicate a selection of subclones. The red line shows a lowess regression to indicate the overall relation between abundance and affinity.
EFFECT ON GC response
Centroblast (CB; blue)
Centrocyte (CC; green),
Total cell count (red).
For each of the 16 parameter changes a triple of CB, CC, and total cell counts was calculated. The circles denote the mean counts obtained at the normal parameter levels.
10% change

## Slide 32
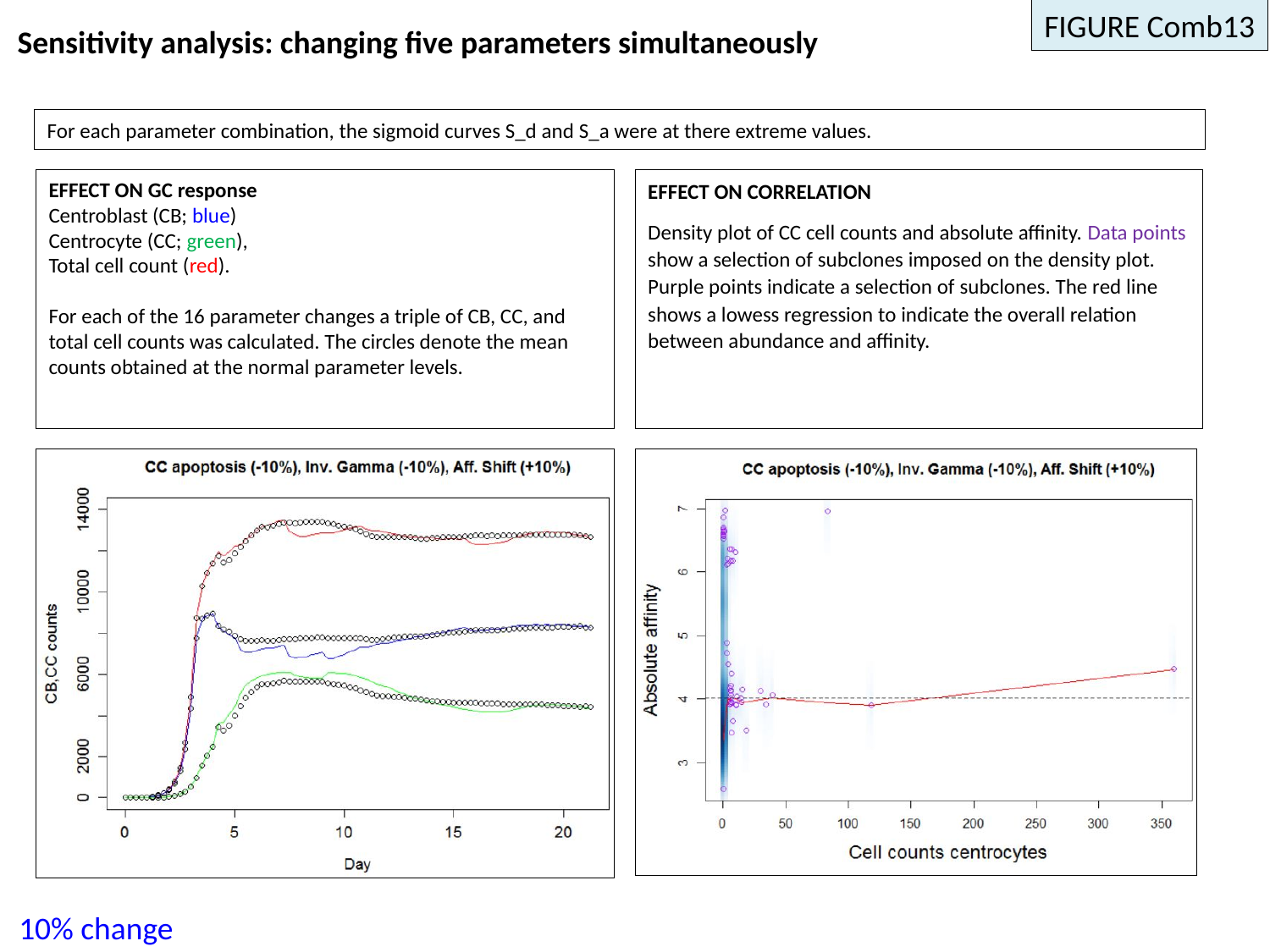

FIGURE Comb13
Sensitivity analysis: changing five parameters simultaneously
For each parameter combination, the sigmoid curves S_d and S_a were at there extreme values.
EFFECT ON CORRELATION
Density plot of CC cell counts and absolute affinity. Data points show a selection of subclones imposed on the density plot. Purple points indicate a selection of subclones. The red line shows a lowess regression to indicate the overall relation between abundance and affinity.
EFFECT ON GC response
Centroblast (CB; blue)
Centrocyte (CC; green),
Total cell count (red).
For each of the 16 parameter changes a triple of CB, CC, and total cell counts was calculated. The circles denote the mean counts obtained at the normal parameter levels.
10% change

## Slide 33
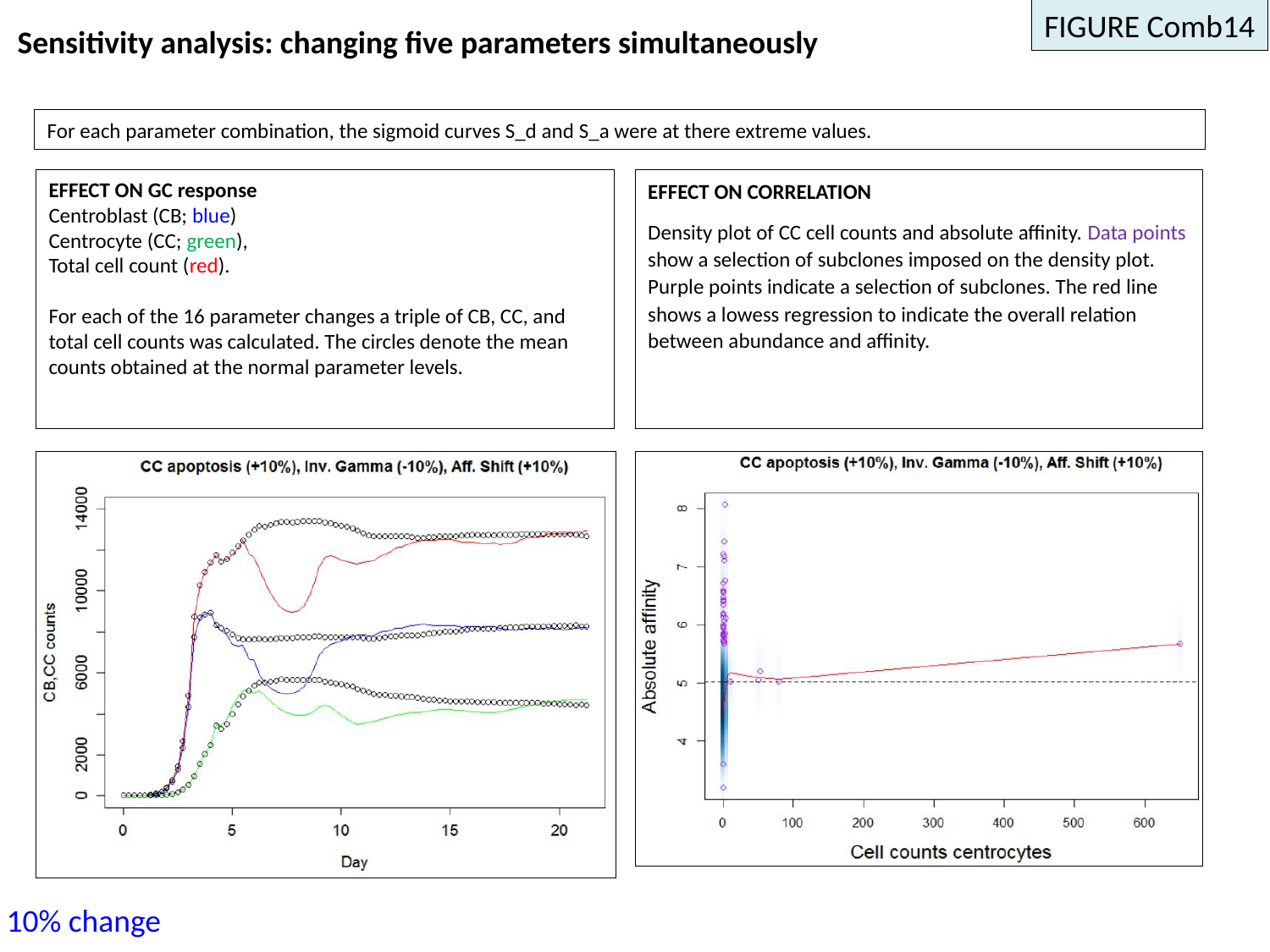

FIGURE Comb14
Sensitivity analysis: changing five parameters simultaneously
For each parameter combination, the sigmoid curves S_d and S_a were at there extreme values.
EFFECT ON CORRELATION
Density plot of CC cell counts and absolute affinity. Data points show a selection of subclones imposed on the density plot. Purple points indicate a selection of subclones. The red line shows a lowess regression to indicate the overall relation between abundance and affinity.
EFFECT ON GC response
Centroblast (CB; blue)
Centrocyte (CC; green),
Total cell count (red).
For each of the 16 parameter changes a triple of CB, CC, and total cell counts was calculated. The circles denote the mean counts obtained at the normal parameter levels.
10% change

## Slide 34
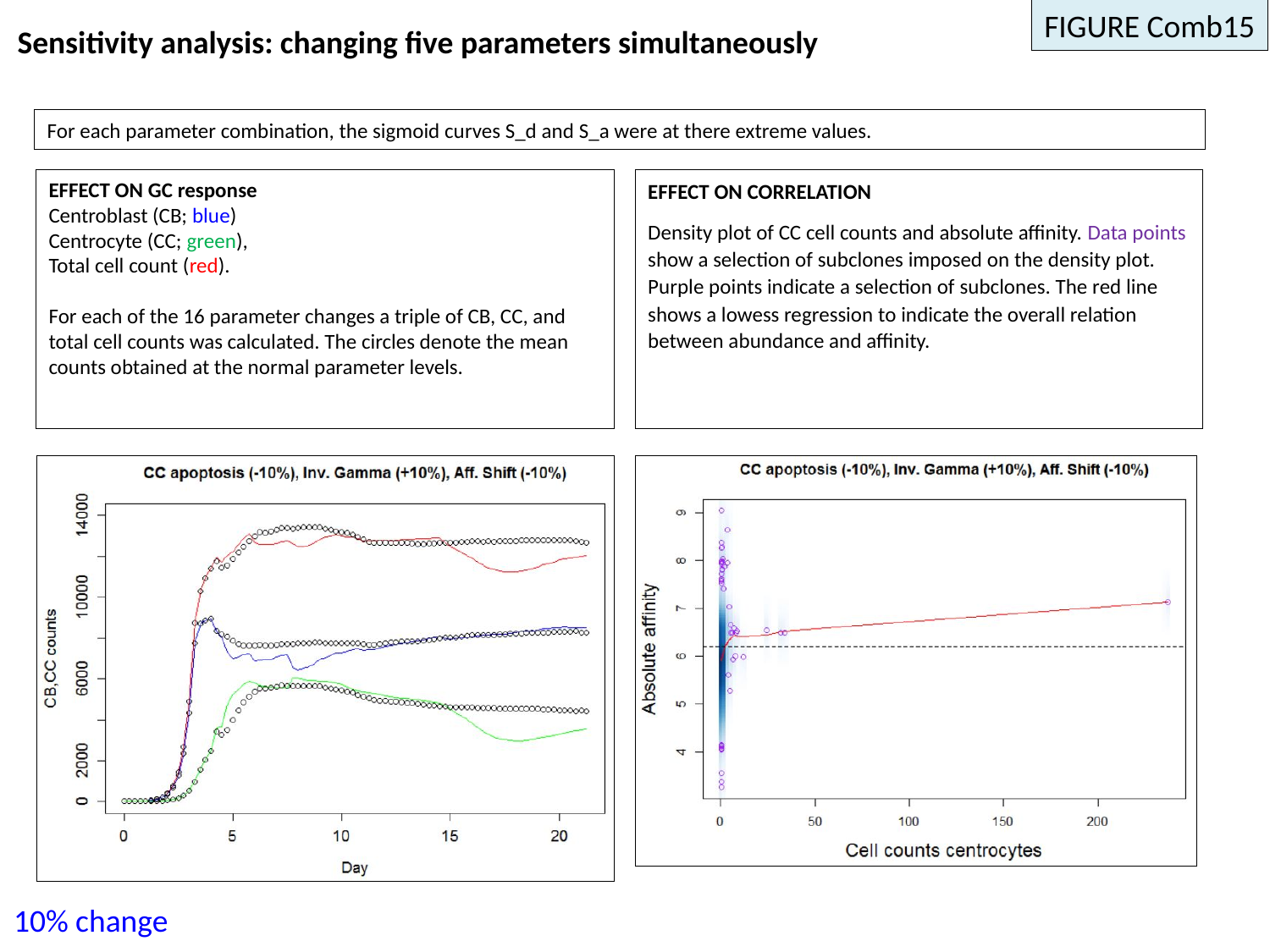

FIGURE Comb15
Sensitivity analysis: changing five parameters simultaneously
For each parameter combination, the sigmoid curves S_d and S_a were at there extreme values.
EFFECT ON CORRELATION
Density plot of CC cell counts and absolute affinity. Data points show a selection of subclones imposed on the density plot. Purple points indicate a selection of subclones. The red line shows a lowess regression to indicate the overall relation between abundance and affinity.
EFFECT ON GC response
Centroblast (CB; blue)
Centrocyte (CC; green),
Total cell count (red).
For each of the 16 parameter changes a triple of CB, CC, and total cell counts was calculated. The circles denote the mean counts obtained at the normal parameter levels.
10% change

## Slide 35
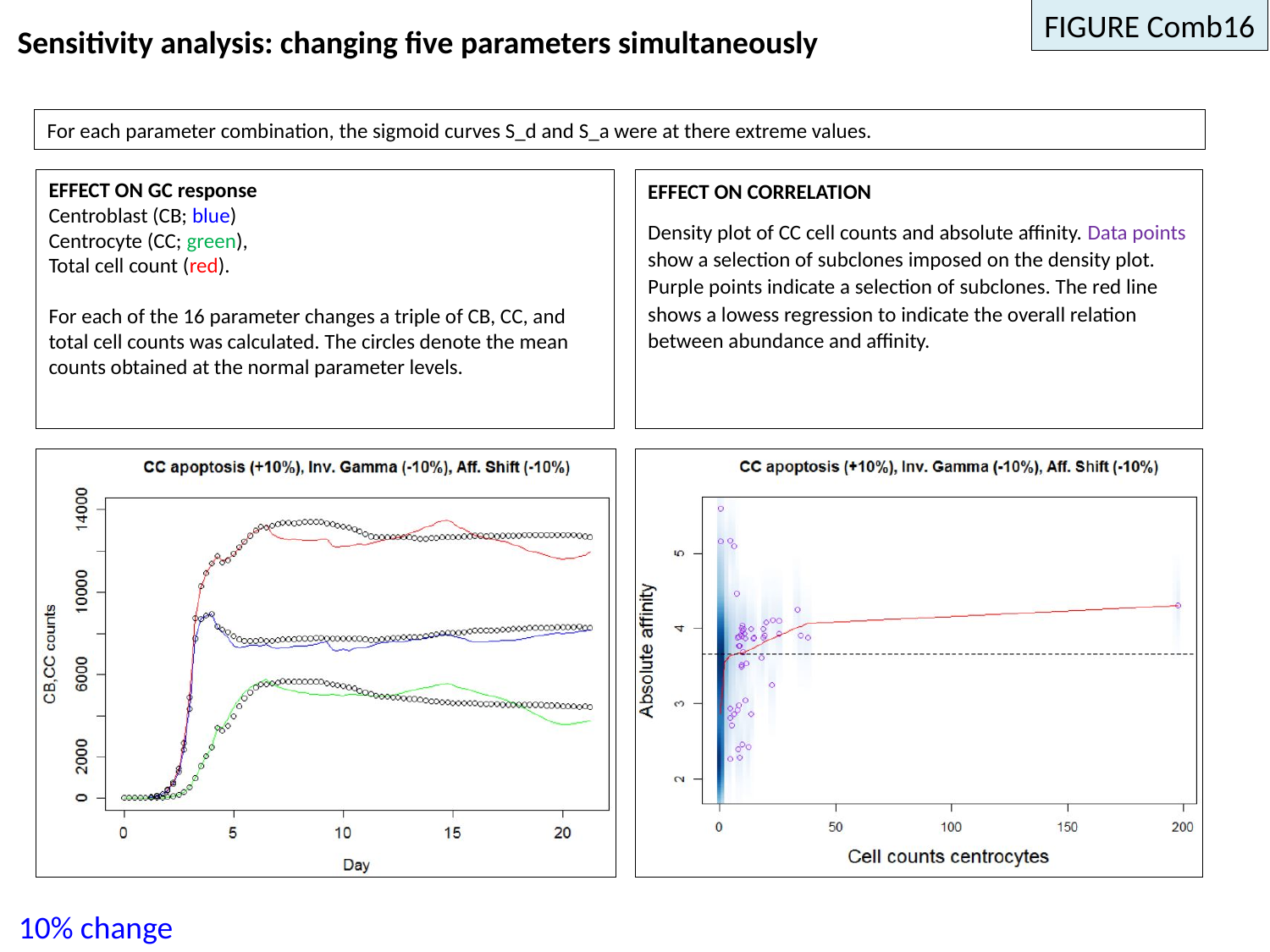

FIGURE Comb16
Sensitivity analysis: changing five parameters simultaneously
For each parameter combination, the sigmoid curves S_d and S_a were at there extreme values.
EFFECT ON CORRELATION
Density plot of CC cell counts and absolute affinity. Data points show a selection of subclones imposed on the density plot. Purple points indicate a selection of subclones. The red line shows a lowess regression to indicate the overall relation between abundance and affinity.
EFFECT ON GC response
Centroblast (CB; blue)
Centrocyte (CC; green),
Total cell count (red).
For each of the 16 parameter changes a triple of CB, CC, and total cell counts was calculated. The circles denote the mean counts obtained at the normal parameter levels.
10% change
