# Supplementary material for: Computational Model Reveals Limited Correlation between Germinal Center B-Cell Subclone Abundancy and Affinity: Implications for Repertoire Sequencing
Source: Front Immunol. 2017 Mar 6;8:221. doi: 10.3389/fimmu.2017.00221 (PMC5337809; doi:10.3389/fimmu.2017.00221)
Supplement: Supplementary file 1 [file Data_Sheet_1.ZIP › data sheet 1/Supplementary Information/VanKampen-SupplementaryInformation.pdf]

Supplementary Material: Computational model reveals limited correlation between germinal centre B-cell subclone abundancy and affinity: implications for repertoire sequencing.

Polina Reshetova, Barbera D.C. van Schaik, Paul L. Klarenbeek, Marieke E. Doorenspleet, Rebecca E.E. Esveldt, Paul-Peter Tak, Jeroen E.J. Guikema, Niek de Vries, Antoine H.C. van Kampen*

*Correspondence:
A.H.C. van Kampen, Bioinformatics Laboratory, Academic Medical Center, University of Amsterdam, Meibergdreef 9, 1105AZ Amsterdam, the Netherlands
a.h.vankampen@amc.uva.nl

1 CERVICAL LYMPH NODE BCR REPERTOIRE DATASET

Lymph node sample LN25 may have included plasma cells that contain much more RNA than GC B cells Kelley and Perry (1986) and, therefore, may distort the correlation between cellular abundance and read count. Therefore, we also analyzed the BCR repertoire obtained from a draining cervical lymph node sample from a patient with chronic multiple sclerosis (sample M5, lymph node B section 2) Stern et al. (2014). CD138+ plasma cells, which often accompany B cells in MS lesions, were not observed in this sample. Immunoglobulin (Ig) variable region heavy chain libraries were constructed from an autopsy sample. RNA was reverse transcribed into cDNA using gene-specific primers mapping to the constant region of the Ig heavy chain. Resultant cDNA was amplified by PCR in a multiplex reaction using primer sets for all IGHV (n=45) and constant regions (n=6). The data is available through NCBIs Bioproject database (http://www.ncbi.nlm.nih.gov/bioproject/248475). We analyzed the data as described in the method section in the main text. The data comprises 12,917 subclones corresponding to 327,806 sequence reads. Supplementary Figure S1 and Supplementary Table S1 show the results of the analysis.

The results obtained from this analysis are similar as those obtained for LN25 and also support the simulation results. First, the subclones (red dots) corresponding to a single B-cell lineage for each of V-J families show a large variation in frequency, which we also observe in LN25 and our simulated data. Secondly, the number of expanded subclones is 2.1%, which is comparable to the percentages we observed from LN25 (0.8%) and from the simulation results (0.3-1.0%). Thirdly, from Supplementary Table S1 we observe a larger number (280) of different subclones in the largest lineage compared to LN25 (35) which is likely due to the much higher sequencing depth for the M2 sample. The M2 diversity supports the subclonal diversity observed in our simulations. In summary, LN25 and M5 provide similar support for our model. The main difference between LN25 and M5 is the amount of expansion of the most expanded subclone, which is 77.1% in M5

and only a few percent in LN25 and our simulation results. However, as noted in the discussion section, presently our model is not capable of controlling different levels of expansion.

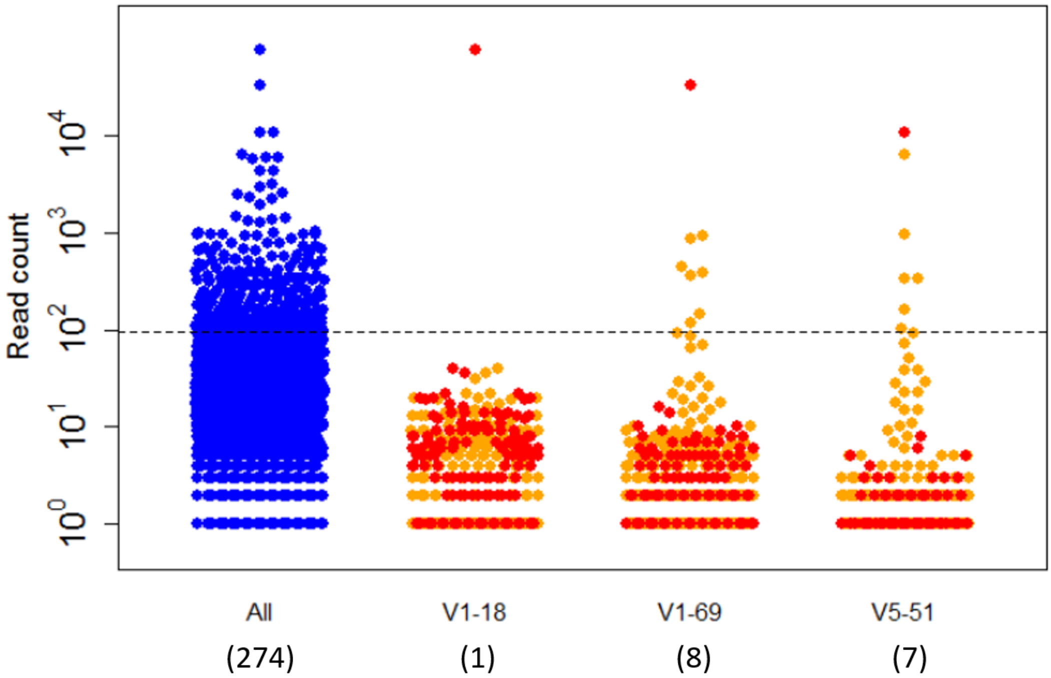

Figure S1. Subclones measured in a cervical lymph node sample (M5, Lymph node B, section 2) from a chronic multiple sclerosis patient. The blue points show the read counts for all 12,917 subclones measured in this sample (274 expanded subclones). The expansion threshold ($T = 94$) is determined from the all M5 subclones, and indicated by the dashed line. Subclones of the three most abundant V-J combinations are shown in orange. The red dots indicate the subclones corresponding to the largest lineages within each V-J family. The numbers in the parenthesis show the number of expanded subclones in the selected V-J subsets.

Supplementary Table 1. Selected B-cell subclones from sample M5, cervical lymph node B section 2

Subclone	Total		Largest cluster (lineage)	
	Subclones	Reads	Subclones	Reads
V1.18 J3 (pep)	139	74,903	125	74,830
V1.69 J5 (pep)	207	36,518	98	32,594
V5.51 J4 (pep)	173	19,457	57	10,553
**Total (pep)**	519	130,878	280	117,977

V and J nomenclature following IGMT Lefranc et al. (2003); Giudicelli et al. (2005). Subclones are defined as unique peptides (pep) with unique V and J assignment and a unique CDR3 sequence. For each V-J family the number of subclones and corresponding number of sequence reads are shown. The selected clusters for the given V-J segments correspond to the largest cluster of subclones having ≤ 2 *differences at nucleotide or peptide level.*

2 SENSITIVITY ANALYSIS

To determine the sensitivity of the model output for changes in parameters we performed a sensitivity analysis in which we changed a selection of individual parameters. In addition, we performed an sensitivity analysis in which we changed a combination of five selected parameters.

2.1 Single parameter changes

We varied a seleted number of individual parameters by +/-10% (Supplementary Table S2) resulting in 14 simulations. The parameters k and n defining the sigmoid curves S_a were also included in the sensitivity analysis. However, since these curves are already far to the left (see Figure 3 in main text), we changed both parameters such that these curves were only shifted to the right. For normal parameter values (k=0.06, n=1 for differentiation (S_d), and k=0.1, n=4 for apoptosis (S_a)) the relative affinities are 0.07 and 0.11 respectively at $S_{a,d} = 0.5$. We changed parameters k and n such that the curves shift to a relative affinity of 0.17 and 0.21 at $S_{a,d} = 0.5$. This corresponds to relative changes of about 50%. For the parameters of the inverse gamma distribution we only changed r, which controls the width of the peak. Differentiation and apoptosis rates of plasma and memory cells were not included in the analysis as these do not directly affect the model response. The capacity A was not changed as this parameter controls the maximum size of the germinal centre. The results of the analysis are shown in the supplementary PowerPoint file that includes all 14 figures of the GC response (CB, CC, total cells counts) and the corresponding figures showing the correlation between the CC cell count and affinity.

Supplementary Table 2. Sensitivity analysis changing 14 individual parameters

Parameter	Normal value	Variation
Proliferation rate (ρ_{CB})	4	$\pm$10%
Differentiation rate ($\eta_{CB\to CC}$)	6	$\pm$10%
Differentiation rate ($\eta_{CC\to CB}$)	1	$\pm$10%
Apoptosis rate (μ_{CC})	4	$\pm$10%
Affinity shift (σ)	0.1	$\pm$10%
Inverse gamma (r)	0.3	$\pm$10%
Sigmoid curve (S_d)	k=0.06 , n=1	k=0.16, n=1.5
Sigmoid curve (S_a)	k=0.1 , n=4	k=0.2, n=5

<u>Results</u>
The model turns out to be quite robust against changes in individual parameters. For most simulations, the overall GC response (CB, CC, and total cell counts) is close to the mean curves obtained from the 15 simulations presented in the main text with parameters at their normal levels. We observe that increasing the proliferation rate (Figure *Single7* in the PowerPoint file) does not have a large effect on the GC response which is likely caused by the capacity factor A that controls the maximum GC size. However, decreasing the proliferation rate (Figure *Single6*) decreases the overall cell count as expected. Parameter r of the inverse gamma distribution has a moderate effect on the overall cell count (Figures *Single12* and *Single13*). Changing the sigmoid curve S_a corresponding to CC apoptosis only affects the CC and total counts as expect (Figure *Single14*). However, the sigmoid curve S_d corresponding to CB differentiation, has a much larger

effect on the CC counts but, suprisingly, leaves the CB counts unaffected (Figure *Single15*). From the correlation plots (Figures *Single1* to *Single14*) it is observed that the maximum CC cell counts (x-axis) and affinities (y-axis) are moderately affected by changes of the parameters. For the simulation presented in the main text the maximum CC count was ˜120 and the maximum affinity value was ˜10 (see Figure 9B in the main text). In the 14 simulations of the sensitivity analysis these vary between ˜50 and ˜300 for CC counts, and between ˜4.5 to ˜9 for the affinity. However, in all simulations the trend between CC cell counts and affinity remains unchanged and, therefore, support the conclusions from this paper.

2.2 Changing five parameters simultaneously

To challenge the model even further, we performed a sensitivity analysis in which we changed five parameters simulteneously to two different levels (5% and 10%) (Supplementary Table S3) resulting in 16 simulations. The table includes the three parameters that are tested at ±5% or ±10% deviation from their normal value (given in Supplementary Table S2). The parameters (k, n) for the sigmoid curves S_d and S_a are always held at their extreme upper level during the simulations (Supplementary Table S2). The results of the analysis are shown in the supplementary PowerPoint file that includes all 16 figures of the GC response (CB, CC, total cells counts) and the corresponding figures showing the correlation between the CC cell count and affinity.

Supplementary Table 3. Sensitivity analysis changing combination of five parameters

**Run**	**Apoptosis rate (μ_{CC})**	**Affinity shift (σ)**	**Inverse gamma (r)**
1	$-level\%$	-level%	-level%
2	$-level\%$	-level%	+level%
3	$-level\%$	+level%	-level%
4	$-level\%$	+level%	+level%
5	$+level\%$	-level%	-level%
6	$+level\%$	-level%	+level%
7	$+level\%$	+level%	-level%
8	$+level\%$	+level%	+level%

level denotes the level of deviation (±5% or ±10%) from the normal parameter values. For each of these runs the sigmoid curves $S_{a,d}$ are kept at their extreme levels as specified in Supplementary Table S2).

Results (±5%)
Parameters value changes of ±5% have a moderate effect on the GC response (CB, CC, total cell counts) except for the change that involved apoptosis rate (-5%), inverse gamma distribution (+5%), and the affinity shift (-5%) (Figures *Comb1* to *Comb8*). From the corresponding correlation plots it is observed that the maximum CC cell counts (x-axis) and affinities (y-axis) are moderately affected by changes in the parameters. For the simulation presented in the main text the maximum CC count was ˜120 and the maximum affinity value was ˜10 (Figure 9B) main text). In the sensitivity analysis these vary between ˜150 and ˜500 for CC counts, and between ˜4.5 to ˜10 for the affinity. However, in all simulations the trend between CC cell counts and affinity remained unchanged and, therefore, support the conclusions from this paper.

Results ($\pm$10%)

Parameters value changes of $\pm$10% have a strong effect on GC response (CB, CC, total cell counts) for half of the simulations, as is shown in Figures *Comb10*, *Comb11*, *Comb12*, and *Comb14*. The corresponding correlation plots show also larger effects for the maximum CC cell counts (x-axis) and affinities (y-axis), which may now reach values > 1000 and > 14 respectively. However, in all simulations (Figures *Comb9* to *Comb16*), the trend between CC cell counts and affinity remains unchanged and, therefore, support the conclusions from this paper.

3 SUPPLEMENTARY FIGURES

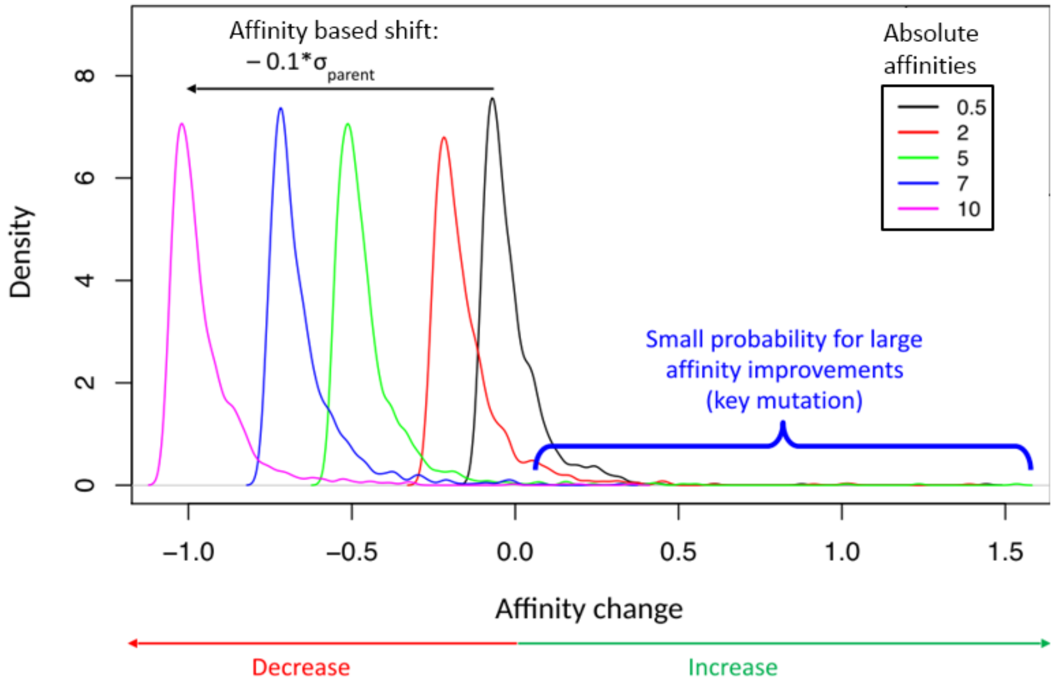

Figure S2. Distribution $f(\sigma)$ used to change affinity of mutated subclones. A mutation may decrease or increase the affinity of a B-cell. There is a small chance of making a large affinity improvements (representing key mutations). The distribution is shifted to the left with $0.1 * \sigma_{parent}$ for cells with higher affinities to decrease the chance for further improvements.

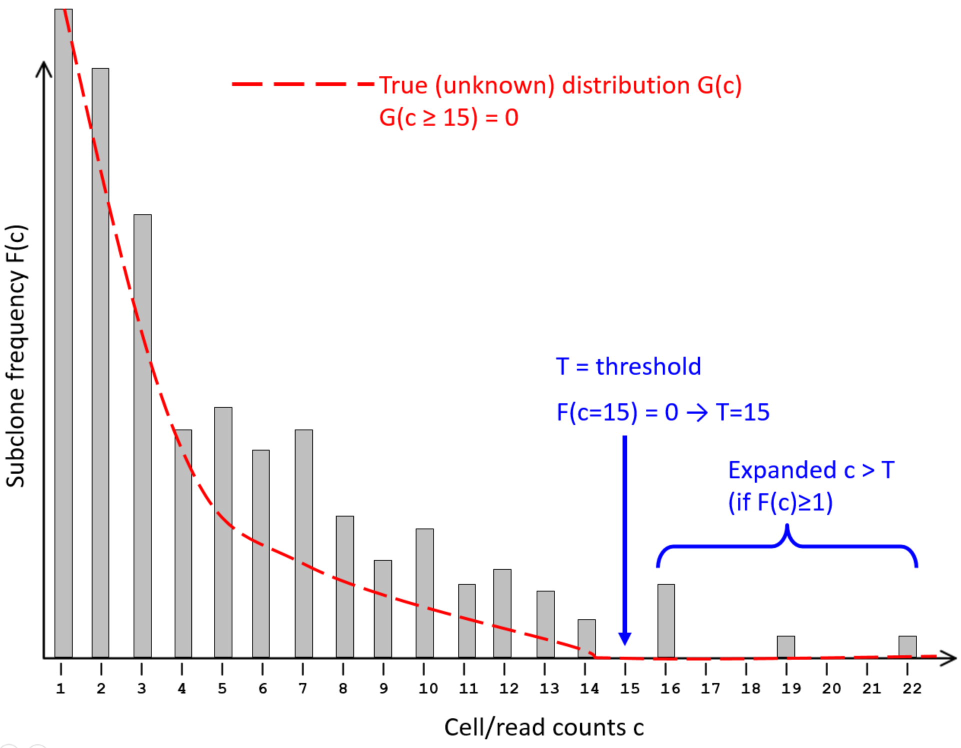

Figure S3. Determination of threshold for expanded subclones. See main text for further explanation.

REFERENCES

Kelley DE, Perry RP. Transcriptional and posttranscriptional control of immunoglobulin mRNA production during B lymphocyte development. *Nucleic Acids Res.* **14** (1986) 5431–5447.

Stern JN, Yaari G, Vander Heiden JA, Church G, Donahue WF, Hintzen RQ, et al. B cells populating the multiple sclerosis brain mature in the draining cervical lymph nodes. *Sci Transl Med* **6** (2014) 248ra107.

Lefranc M, Pommié C, Ruiz M, Giudicelli V, Foulquier E, Truong L, et al. IMGT unique numbering for immunoglobulin and T cell receptor variable domains and Ig superfamily V-like domains. *Dev. Comp. Immunol.* **27** (2003) 55–77.

Giudicelli V, Chaume D, Lefranc MP. IMGT/GENE-DB: a comprehensive database for human and mouse immunoglobulin and T cell receptor genes. *Nucleic acids research* **33** (2005) D256–D261. doi:10.1093/nar/gki010.
